# Genetic Variation in Reproductive Investment Across an Ephemerality Gradient in *Daphnia pulex*

**DOI:** 10.1093/molbev/msac121

**Published:** 2022-06-01

**Authors:** Karen B Barnard-Kubow, Dörthe Becker, Connor S Murray, Robert Porter, Grace Gutierrez, Priscilla Erickson, Joaquin C B Nunez, Erin Voss, Kushal Suryamohan, Aakrosh Ratan, Andrew Beckerman, Alan O Bergland

**Affiliations:** Department of Biology, University of Virginia, Charlottesville, VA, USA; Department of Biology, James Madison University, Harrisonburg, VA, USA; Department of Biology, University of Virginia, Charlottesville, VA, USA; School of Biosciences, Ecology and Evolutionary Biology, University of Sheffield, Sheffield, UK; Department of Biology, University of Marburg, Marburg, Germany; Department of Biology, University of Virginia, Charlottesville, VA, USA; Department of Biology, University of Virginia, Charlottesville, VA, USA; Department of Biology, University of Virginia, Charlottesville, VA, USA; Department of Biology, University of Virginia, Charlottesville, VA, USA; Department of Biology, University of Virginia, Charlottesville, VA, USA; Department of Biology, University of Virginia, Charlottesville, VA, USA; Department of Integrative Biology, UC Berkeley, Berkeley, CA, USA; MedGenome Inc., Foster City, CA, USA; Center for Public Health Genomics, University of Virginia, Charlottesville, VA, USA; Department of Public Health Sciences, University of Virginia, Charlottesville, VA, USA; School of Biosciences, Ecology and Evolutionary Biology, University of Sheffield, Sheffield, UK; Department of Biology, University of Virginia, Charlottesville, VA, USA

**Keywords:** facultative sex, *Daphnia*, male production, quantitative genetics, population genomics

## Abstract

Species across the tree of life can switch between asexual and sexual reproduction. In facultatively sexual species, the ability to switch between reproductive modes is often environmentally dependent and subject to local adaptation. However, the ecological and evolutionary factors that influence the maintenance and turnover of polymorphism associated with facultative sex remain unclear. We studied the ecological and evolutionary dynamics of reproductive investment in the facultatively sexual model species, *Daphnia pulex*. We found that patterns of clonal diversity, but not genetic diversity varied among ponds consistent with the predicted relationship between ephemerality and clonal structure. Reconstruction of a multi-year pedigree demonstrated the coexistence of clones that differ in their investment into male production. Mapping of quantitative variation in male production using lab-generated and field-collected individuals identified multiple putative quantitative trait loci (QTL) underlying this trait, and we identified a plausible candidate gene. The evolutionary history of these QTL suggests that they are relatively young, and male limitation in this system is a rapidly evolving trait. Our work highlights the dynamic nature of the genetic structure and composition of facultative sex across space and time and suggests that quantitative genetic variation in reproductive strategy can undergo rapid evolutionary turnover.

## Introduction

The ability to oscillate between sexual and asexual reproduction is widely regarded as an optimal strategy that enables species to capitalize on the benefits of asexual reproduction, whereas also mitigating its detrimental effects (Meirmans et al. 2012; Hartfield 2016). Facultative sexuality is common across the tree of life (Hartfield 2016; Kokko 2020), and its relative deficit among metazoans has been regarded as one of the most important problems in evolutionary biology (Burke and Bonduriansky 2017). In many facultatively sexual species, sexual reproduction is coupled with dormancy and dispersal (Cáceres and Soluk 2002; Simon et al. 2002; Ebert 2005; Schröder 2005; Hadany and Otto 2007; Gerber and Kokko 2018). Thus, the relative investment into sexual versus asexual reproduction will likely be subject to strong selection imposed by ecological features such as the duration of the growing season (Hairston and Van Brunt 1994; Innes 1997; Smith and Snell 2012) or the predictability of environmental change (Franch-Gras et al. 2017; Tarazona et al. 2017). As a consequence, reproductive investment strategies are likely subject to local and rapid adaptation (Walsh 2013). Genetic variation in reproductive investment strategies varies across broad biogeographical scales (Lynch 1984; Dedryver et al. 2001; Hörandl 2006; Tilquin and Kokko 2016) and has also been observed within populations (Vorburger, Sunnucks, et al. 2003; Barbuti et al. 2012), suggesting the action of both directional and balancing selection.

Seasonal fluctuations in habitat suitability—ephemerality—can drive the evolution of plasticity and also maintain genetic variation in reproductive investment strategies. In facultative sexuals, there is a direct trade-off between sexual and asexual reproduction, and the relative costs and benefits of these modes of reproduction vary across the season (Gerber, Kokko, et al. 2018). In highly predictable environments, theory predicts that investment into sex—for example, the switch to male production and sexual female reproduction—will be strongly dependent on environmental conditions and will occur synchronously across the population late in the growing season (Hairston and Munns 1984; Gerber, Booksmythe, et al. 2018). However, when habitat suitability varies unpredictably, investment into sexual versus asexual reproductive strategies may become decoupled from environmental cues causing investment into sex to occur throughout the growing season (Spencer et al. 2001; Gerber, Booksmythe, et al. 2018). In such a scenario, the relative investment into sex may be genetically variable and subject to spatially and temporally varying selection pressures. For instance, lineages that invest more into sex will produce more offspring that can survive the unfavorable season and thus sex promoting alleles will be at high frequency early in the growing season. Clonal competition during the growing season will select for lineages that invest less into sex (Innes and Singleton 2000; Carmona et al. 2009), and thus sex promoting alleles will decline in frequency. Temporal fluctuations in the environment can therefore promote genetic variation and can be seen as a form of balancing selection (Gillespie and Turelli 1989; Turelli and Barton 2004). However, whether alleles underlying variation in reproductive investment strategy can be stably maintained in a population (Hedrick 1976), or whether they are quickly lost and regained (Bürger and Gimelfarb 2002; Cvijović et al. 2015), remains an open question.

*Daphnia* are excellent models to address this question and thereby gain insight into the evolutionary dynamics of variation in reproductive investment. *Daphnia* are planktonic crustaceans that survive unfavorable conditions (e.g., drying, freezing, high predation) via sexually produced resting eggs (Cáceres 1997; Brendonck and De Meester 2003). Multiple species of *Daphnia* harbor genetic variation in reproductive investment strategies (Innes and Dunbrack 1993; Hebert and Finston 2001; Tessier and Cáceres 2004; Galimov et al. 2011; Roulin et al. 2013). The most extreme example is the transition from cyclic parthenogenesis to obligate parthenogenesis observed in North American *Daphnia pulex* (Innes and Hebert 1988; Paland et al. 2005; Lynch et al. 2008; Tucker et al. 2013). The loss of sex originated as a result of hybriziation between the North American *D. pulex* and *D. pulicaria*, and has been localized to regions on chromosomes 8 and 9 (Lynch et al. 2008; Tucker et al. 2013). Tucker et al. (2013) found that extant asexual lineages exhibit an average age of 22 years, much younger than the estimated age of the entire asexual clade (∼1,250 years), suggesting that recurrent turnover might be occurring. The rate of male production is also genetically variable in *Daphnia* (Innes and Dunbrack 1993; Tessier and Cáceres 2004; De Meester et al. 2006; Fitzsimmons and Innes 2006; Galimov et al. 2011; Roulin et al. 2013, 2015). QTL mapping of genetic variation of male production in North American *D. pulex* and European *D. magna* has identified multiple loci found in nonoverlapping regions of the genome suggesting a distinct genetic architecture (Roulin et al. 2013; Reisser et al. 2017; Ye et al. 2019). Taken together, the scope of genetic variation observed for reproductive investment strategies across multiple systems is consistent with a model that investment is a dynamically evolving trait within species subject to constant allelic turnover.

**Fig. 1.**
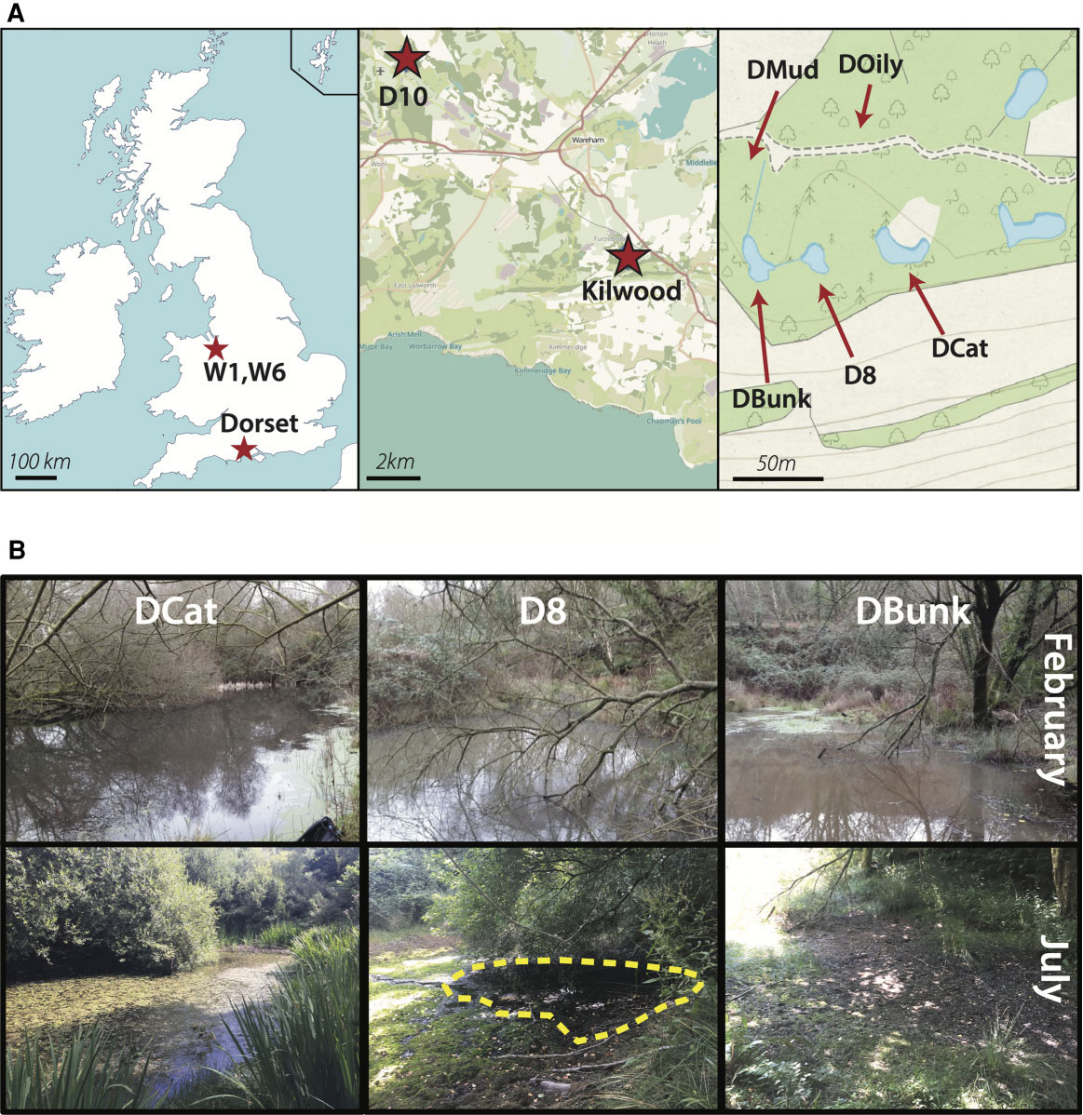
Location and features of the focal ponds. (*A*) Location of *D. pulex* sampling sites. The left panel shows the location of the focal populations, Dorset, and two distant ponds in Wales. The middle panel zooms in on the Dorset region and shows a neighboring pond, D10, along with the focal metapopulation at the Kilwood Coppice Nature Reserve, situated just north of the Purbeck hills. The right panel shows the location of the focal ponds DCat, D8, and DBunk, as well as two additional sampled ponds, DOily and DMud at the Kilwood Coppice Nature Reserve. (*B*) Pictures depicting water level in the three focal ponds in February (2020) and July (2018), illustrating their variance in ephemerality. The outlined area in the July picture of D8 shows the borders of a remnant puddle. Map credits and references shown in the data accessibility statement.

To test the basic idea that genetic variation in reproductive investment shows signals of recurrent allelic turnover and varies across a localized ephemerality gradient, we characterized the patterns of clonal diversity and the genetic basis of variation in male production of *D. pulex* across a series of small ponds in the southern region of the United Kingdom (fig. 1*A*–*C*). We focused our attention on three ponds located in Dorset (fig. 1*A*) that were found to vary in ephemerality and duration of asexual reproduction. Using whole-genome sequencing of hundreds of clones sampled across multiple years as well as lab phenotyping experiments, we show that the genetic composition of the populations varies in a manner consistent with ephemerality, that genetic variation in male production has an independent evolutionary origin from previously identified systems, and that this variation is relatively young, suggesting rapid genetic turnover of an evolutionarily unstable system.

## Results

### Focal Ponds Vary in Ephemerality

To characterize patterns of ephemerality for the three focal ponds (DBunk, DCat, and D8, fig. 1*A*), we assessed water level over the course of the growing season and the presence of various *Daphnia* species. We documented that the ponds varied in ephemerality: DBunk dried completely each summer, D8 periodically dried to a large puddle (e.g., during heatwaves in 2018 and 2019), and DCat exhibited relatively minor reductions in water level (fig. 1*B*, supplementary table S1, Supplementary Material online). The geographically distant Dorset pond (11 km from focal ponds), D10, also exhibited only minor changes in water level. The ponds rapidly filled in the fall after heavy rains (supplementary fig. S1, Supplementary Material online). Thus, the growing season for these Daphnia populations is likely from fall into late spring or early summer. Multiple Cladocera species were found in the ponds: *D. pulex* was found in all ponds, *D. obtusa* only found in the most ephemeral pond, DBunk, and *Simocephalus* spp. found in the warmer spring months.

### Clonal Diversity and Mating Dynamics Vary in a Manner Consistent with Ephemerality

**Fig. 2.**
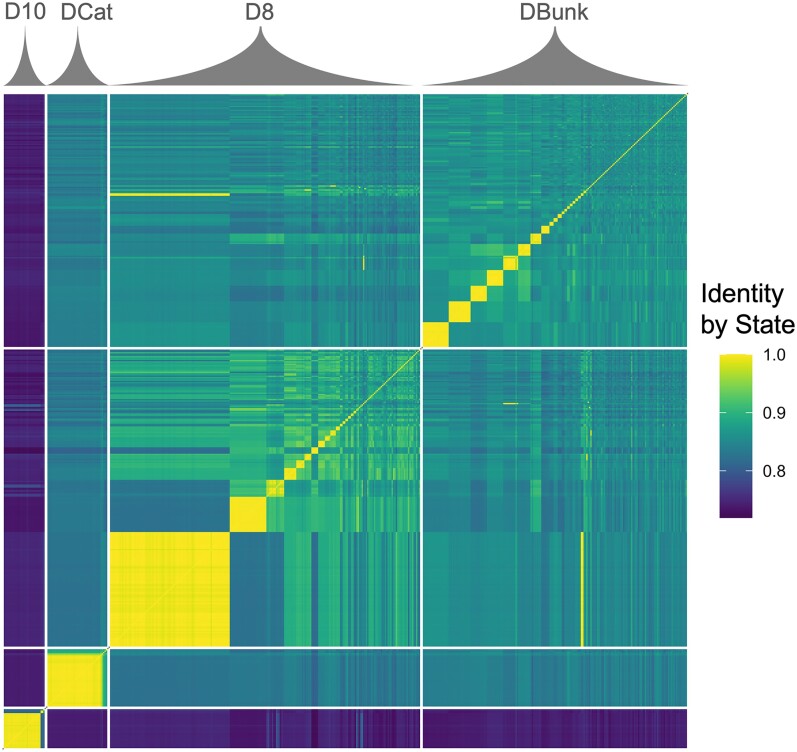
Assignment of clonal identity via IBS. Pairwise IBS matrix generated using whole-genome sequence data. Matrix includes 498 diploid genomes from D10, DCat, D8, and DBunk. The largest clonal group identified in D8 is superclone A, the second largest is superclone C.

To characterize the clonal diversity of *D. pulex* from the focal ponds, we sequenced ∼500 *D. pulex* genomes from samples collected across three consecutive years. These samples were taken between March and May. Thus, sampled populations had been exposed to clonal selection for at least several months, as ponds that went dry the previous summer refilled in the fall. These genomes consisted of 169 individuals fixed in the field and 329 lab-maintained isofemale lines (supplementary table S3, Supplementary Material online). Reads were aligned to a high-quality, chromosome-scale reference genome (L50 = 6, N50 = 10,449,493 bp, total assembly length = 127,796,161 bp, BUSCO score = 95%; supplementary fig. S2, Supplementary Material online) derived from a *D. pulex* clone previously sampled from D8 (clone D8.4A, sampled in 2012). We assigned individuals to asexually related, clonal lineages based on pairwise identity by state (IBS, fig. 2 and supplementary fig. S3*A*, Supplementary Material online), and reconstructed a pedigree using IBS_0_ and kinship estimates. We refer to clonal lineages that were sampled multiple times in the field as “superclones” (sensu Vorburger, Lancaster, et al. 2003) and individuals isolated from the field and propagated in the lab as “isofemale lines.”

**Fig. 3.**
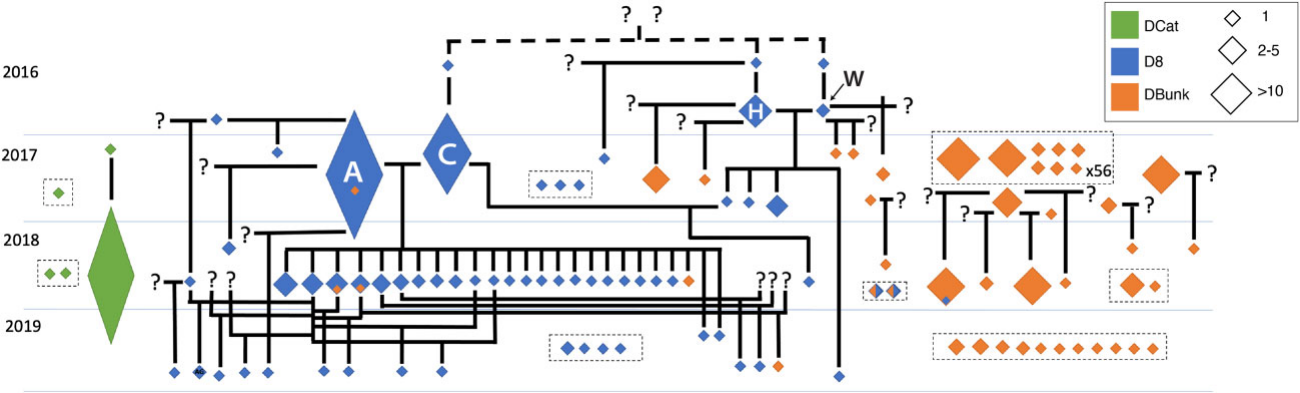
Inferred pedigree based on kinship and IBS_0_. Each diamond is a superclone, with the size of the diamond proportional to the abundance of the superclone. Diamonds present across multiple years indicate superclones sampled across multiple time points. Vertical lines indicate within-clone mating, whereas question marks indicate inferred, unsampled clones.

The relative abundance of clonal lineages varied across time and space consistent with variation in pond ephemerality (figs. 2 and 3, supplementary fig. S4, Supplementary Material online). The most permanent pond, DCat, was dominated by a single superclone, leading to low clonal diversity across 3 years (Shannon’s *H* range: 0–1.33, mean = 0.51, supplementary fig. S4, Supplementary Material online). In contrast, the most ephemeral pond, DBunk, exhibited the highest clonal diversity (*H*: 2.04–3.59, mean = 2.67) with all time points having multiple clones present. D8 was intermediate and exhibited large fluctuations in clonal diversity across years (*H*: 1.02–3.05, mean = 2.09). D8 showed low clonal diversity with only two dominant clones present after an extended period of asexual reproduction (2016–2017). D8 then showed higher levels of diversity after the pond dried to a puddle (2018, 2019) as only sexual offspring survived. The persistence of clonal lineages across years also fits with observed patterns of ephemerality, with clonal lineages observed across multiple years in both DCat and D8, but never in DBunk. In addition, patterns of mating varied with ephemerality. Consistent with reduced clonal diversity, within-clone mating and inbreeding between sexually produced siblings were observed only in the more permanent ponds (i.e., DCat and D8, but not DBunk; fig. 3).

Patterns of genetic diversity within clonal lineages were consistent with extended periods of asexual reproduction. We found that new mutations within clonal lineages had an elevated nonsynonymous-to-synonymous ratio (*p*_N_/*p*_S_) relative to mutations in the same frequency class among outcrossing, sexually related clones (supplementary fig. S5, Supplementary Material online). An elevated *p*_N_/*p*_S_ is expected during asexual reproduction as new mutations remain in the heterozygous state and are somewhat shielded from selection.

### Genetic Diversity Does Not Vary with Ephemerality

To examine patterns of genetic diversity across space and time, we estimated the average heterozygosity for each individual. Average within-individual heterozygosity among sampled lineages was ∼0.0025 (supplementary fig. S6, Supplementary Material online), about one-third the magnitude of diversity estimates from North American *D. pulex* (Lynch et al. 2017). In contrast to clonal diversity and patterns of mating, within-individual heterozygosity did not consistently vary with ephemerality (supplementary fig. S6, Supplementary Material online), however, within-individual heterozygosity diversity did fluctuate from year to year in D8. The fluctuations in genetic diversity in D8 appear to be driven by this pond’s specific clonal dynamics, mirroring fluctuations in clonal diversity. Within-individual heterozygosity was high in 2016, then dropped in 2017 as two superclones, one of which was the result of within-clone mating, came to dominate the pond. Mating between these two superclones led to an increase in heterozygosity in 2018, with inbreeding between the resulting F1 hybrids leading to another reduction in heterozygosity in 2019.

### Coexistence of Dominant Clones was Observed in the Pond with Intermediate Ephemerality

One intriguing pattern in the spatio-temporal dynamics of clonal diversity was the existence of two superclones in D8 (hereafter referred to as superclones A and C, fig. 3) that were relatively rare in 2016 (both frequency of 5%), came to dominate in 2017 (A: 68% and C: 22%), and then mated such that the majority of the D8 population in 2018 consisted of their F1 hybrids. Superclones A and C are themselves the product of sexual reproduction and are more distantly related than most individuals within the focal ponds (supplementary fig. S3*E*, Supplementary Material online). We examined the genetic relationship between superclones A and C relative to other focal pond lineages and geographically distant *D. pulex* populations using a genome-wide, sliding window analysis of IBS. Although superclones A and C are more genetically divergent than 96.6% of pairs of focal pond lineages, they are less divergent than 100% of geographically distant comparisons (supplementary fig. S7, Supplementary Material online), consistent with a structure analysis that assigns the DCat, D8, and DBunk individuals to different groups than the other samples (supplementary fig. S8, Supplementary Material online). These patterns are consistent with divergence between A and C being the result of alleles segregating within the D8 population, with no need to invoke gene flow from more distant populations.

### Coexisting Dominant Lineages Exhibit a Trade-off between Asexual and Sexual Reproduction

**Fig. 4.**
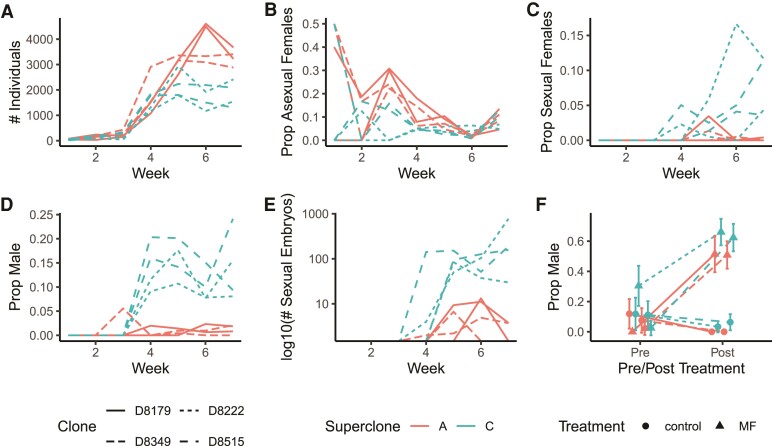
Superclones A and C invest differently in asexual and sexual reproduction. Demographic data over time for A and C isofemale lines propagated in mesocosms. Line types correspond to different isofemale lines. Two isofemale lines were used for each superclones (A: D8–179, D8–349; C: D8–222, D8–515). (*A*) Total population size, (*B*) proportion of females reproducing asexually, (*C*) proportion of females reproducing sexually (producing ephippia), (*D*) proportion of males, (*E*) number of sexually produced embryos graphed on a log 10 scale, (*F*) male production of A and C females when exposed or not exposed to MF. Error bars represent 95% confidence intervals.

**Fig. 5.**
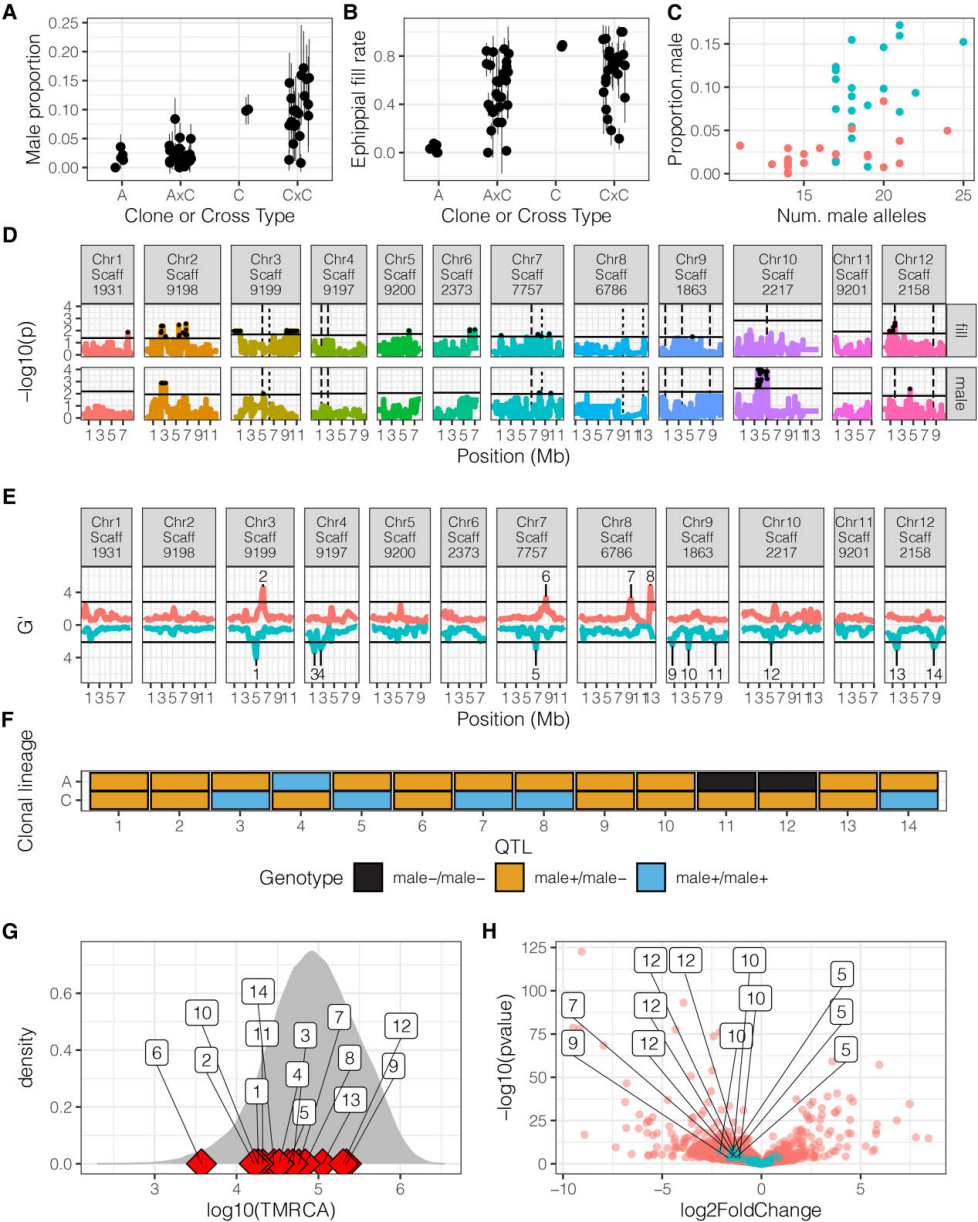
Genetic variation and mapping of variation in male production. Male production (*A*) rate and ephippial fill rate (*B*) in A, C, A×C F1s, and C×C F1s. Points represent isofemale lines and vertical lines represent 95% confidence intervals. (*C*) Male production rate versus the number of *male^+^* alleles across the 14 QTL identified via Pool-Seq (see *E*) for both the A×C cross (red) and C×C cross (blue). Each point represents an isofemale line. See text for statistics on correlation between the number of *male^+^* alleles and male production rate. (*D*) QTL mapping in A×C F1 hybrids for ephippial fill rate and male production identified multiple peaks for each trait. Black points represent QTL regions that pass chromosome level permutation threshold. (*E*) Mapping of male production using pooled field samples identified multiple peaks, with some overlap with the A×C F1 hybrid mapping. To visualize overlap, the pooled sequencing peaks are plotted on the A×C F1 hybrid mapping figure as dashed lines. Horizontal line is the 5% FDR (false discovery rate) threshold. Values above the zero line represent Pool-Seq replicate 1, and those below the zero line represent replicate 2. (*F*) Genotype for superclone A and C at each pooled sequencing QTL. (*G*) TMRCA for the 12 pooled sequencing QTL plotted against the genome-wide distribution. (*H*) RNA-seq identified many genes differentially expressed between superclones A and C, some of which are located near QTL peaks. Blue points are genes within 50 kb of QTL identified in the Pool-Seq, red points are all other genes, and genes that are near the Pool-Seq peaks and are in the top 10% of differential expression genome-wide are labeled with their corresponding QTL number. For both (*G*) and (*H*), the number in the boxes corresponds to the QTL number as identified in (*E*).

The fact that superclones A and C coexisted for some period of time (2016–2017), and then crossed to produce the majority of the population the following year (2018) with no evidence for within-clone mating (C×C or A×A offspring) suggests that either these superclones invest differently in sexual reproduction or they exhibit disassortative mating. A lack of C×C offspring in the 2018 field samples is particularly notable given that superclone C was found to produce ample viable and fertile C×C offspring when maintained in the lab (e.g., fig. 5*A*). In contrast, superclone A rarely produced viable A×A offspring in the lab (e.g., fig. 4*E*), in large part because males were limiting. Although a lack of C×C offspring in the field could be due to inbreeding depression, the observation that selfed Cs produced in the lab exhibited high survival and reproductive ability (see Crossing Experiments Describe the Genetic Architecture of Male Production) suggests differential sexual investment.

To test the hypothesis that superclones A and C have differences in sexual investment, we established mesocosms inoculated with either superclone A or C isofemale lines. We tracked population size and demographic composition over 7 weeks. Daphnia clones can invest in sexual reproduction in two different ways. First, females can continue to reproduce asexually, but produce clonal male offspring instead of females. Second, females can switch to sexual reproduction by producing an ephippium (resting egg case) and then depositing one to two embryos within if fertilized by a male. Thus, we recorded the following metrics for each mesocosm: total population size, proportion asexual females, proportion sexual females (females with ephippia), proportion males, and sexual embryo production. We then tested for a significant difference between superclones A and C using linear mixed effect models and comparing the fit of the model with or without the inclusion of superclone as a fixed effect (lme4 in R; Bates et al. 2015).

We observed a clear trade-off between asexual and sexual reproduction between superclones A and C. Superclone A isofemale lines achieved a higher total population size (fig. 4*A*, *χ*^2^ = 8.8, df = 1, *P* = 0.0030) and maintained a greater proportion of the population as asexually reproducing females for a longer period of time (fig. 4*B*, *χ*^2^ = 6.7, df = 1, *P* = 0.0096) than superclone C. In contrast, superclone C females began producing ephippia earlier (as measured as the proportion of females with ephippia), and to a greater extent, than superclone A females (fig. 4*C*, *χ*^2^ = 14.3, df = 1, *P* = 1.58 × 10^−4^). Perhaps most striking was the difference in male production. Superclone C isofemale lines produced substantially more males than superclone A and began doing so as soon as populations expanded between weeks 3 and 4 (fig. 4*D*, *χ*^2^ = 11.3, df = 1, *P* = 7.70 × 10^−4^). This difference in male production was maintained consistently throughout the rest of the experiment, and was replicable in multiple culture volumes (1L, *χ*^2^ = 14.7, df = 1, *P* = 1.27 × 10^−4^; fig. 5*A*; 250 ml, *χ*^2^ = 7.5, df = 1, *P* = 0.0063; supplementary fig. S9, Supplementary Material online). Differential investment into male production and ephippia led to a marked difference between superclones in sexual embryo production (fig. 4*E*, *χ*^2^ = 5.7, df = 1, *P* = 0.017). Maximum weekly embryo production ranged from 84 to 770 for superclone C mesocosms, but only 5–13 for superclone A mesocosms.

### Unique Mechanisms and Origins of Male Limitation

Male production was the most pronounced phenotypic difference between superclones A and C in the mesocosm experiment. Male production in *Daphnia* is triggered by exposure to methyl farnesoate (MF), the *Daphnia* innate juvenile hormone (Toyota, Miyakawa, Hiruta, et al. 2015). This chemical is produced maternally, often in response to environmental change, and when sensed by developing embryos, causes male development (LeBlanc and Medlock 2015; Toyota, Miyakawa, Yamaguchi, et al. 2015). Thus, in principle, the variation in male production that we observe between A and C could be due either to changes in the propensity to produce MF or in the ability of embryos to detect and respond to this chemical signal. Previous work has shown that nonmale-producing clones in *D. pulex* (Ye et al. 2019) and in *D. magna* (Galimov et al. 2011) fail to produce males in response to exogenous application of MF, indicating that the loss of male production in these clones is associated with a loss of the ability to detect or respond to this chemical cue.

To determine whether low-male production in superclone A is similarly due to a loss of ability to detect or respond to MF, we exposed single A and C females to MF after their first clutch and tracked male production in subsequent clutches. All clones demonstrated a strong response to MF (40–60% male-production rate) compared with controls (0–10%, fig. 4*F*, all data—treatment by time interaction: *χ*^2^ = 76.1, df = 1, *P* < 2.2 × 10^−16^; postexposure data—treatment: *χ*^2^ = 361.3, df = 1, *P* < 2.2 × 10^−16^). Superclone C females produced more males when exposed to MF than superclone A females (*χ*^2^ = 8.4, df = 1, *P* = 0.0038), consistent with C clones being higher male producers. Superclone A females also produced males when not exposed to MF, though at low frequencies (figs. 4*C*, *F* and 5*A* and supplementary fig. S9, Supplementary Material online). Thus, superclone A is more accurately described as low-male producing rather than nonmale producing (sensu Galimov et al. 2011; Ye et al. 2019). The ability of superclone A to respond to the presence of exogenous MF via increased male production suggests its low-male production may be the result of a failure to produce this hormone in response to environmental change, rather than a loss of ability to detect and respond to the chemical cue.

Previous work on variation in male production in North American *D. pulex* has shown that nonmale production can be caused by adaptive introgression from the sister species, *D. pulicaria* (Ye et al. 2019). However, the European *D. pulex* samples that we collected do not show signatures of recent hybridization (supplementary fig. S8*C* and *D*, Supplementary Material online), and the fraction of the genome estimated to be derived from British *D. pulicaria* or British *D. obtusa* is low (<1%). In particular, superclones A and C show trivial amounts of recent ancestry with these two outgroup species (∼0.001%). Altogether, these findings indicate that the genetic architecture and evolutionary dynamics influencing variation in male production in the focal ponds are likely distinct from that observed in nonmale-producing clones in previous work.

### Crossing Experiments Describe the Genetic Architecture of Male Production

Having established that genetic variation for male production exists between superclones A and C, we next examined the genetic architecture of this variation through phenotypic and genetic characterization of A×C (*n* = 22) and C×C (*n* = 20) F1s. We characterized rates of male production by maintaining A×C and C×C F1s, as well as A and C isofemale lines, in crowded conditions over multiple weeks and measured two aspects of male production. First, we tracked the number of sexual embryos per ephippium (hereafter ephippial fill rates) across multiple time points, and also directly characterized the proportion of males at the end of the experiment.

Segregation of male production in A×C F1 hybrids suggests that male production is affected by dominant male-limiting alleles, as most A×C F1 hybrids exhibited low-male production rates, similar to superclone A (fig. 5*A*). However, C×C F1s exhibited transgressive variation with male-production rate (fig. 5*A*) suggesting that C is heterozygous for multiple loci affecting male production. There was also a significant effect of clone on male production when testing the A×C (binomial glm; *χ*^2^ = 97.8, df = 21, *P* = 6.95 × 10^−12^) and C×C crosses (binomial glm; *χ*^2^ = 72.7, df = 19, *P* = 3.30 × 10^−8^) separately, further supporting a heritable component to the observed variation.

To gain insight into the genetic architecture of male production, we sequenced the genomes of F1 individuals and performed QTL mapping for male-production rate and ephippial fill rate. We identified multiple putative QTL for male production in the A×C cross, with the strongest association on chromosome 10 (fig. 5*D*). The large QTL region on chromosome 10 likely represents a single, linked QTL given the high linkage disequilibrium (LD) between associated single nucleotide polymorphism (SNPs) in the region (supplementary fig. S10, Supplementray Material online). We also identified multiple QTLs for ephippial fill rate in the A×C F1 cross that partially overlapped with those identified for male production (fig. 5*D*). Analysis of linkage between the QTL we identified demonstrates that they segregate relatively independently, especially for those QTL found on separate chromosomes (supplementary fig. S10, Supplementary Material online), suggesting that multiple QTL may be independently affecting male production. Mapping of male-production rate and ephippial fill rate in the C×C F1 offspring revealed a signal of association, although the resolution was lower than for A×C due to the inbred nature of this cross (supplementary fig. S11, Supplementary Material online). Overall, these results suggest multiple QTL influence male production in this population.

### Mapping of Male Production using Field Samples Further Supports Multiple QTL

We took a second, complementary approach to investigate the genetic architecture of male production by performing pooled sequencing of males and parthenogenetically reproducing females that were sampled and preserved in the field. We sequenced two pools of males (*N* = 35 each) and two pools of parthenogenetically reproducing females (*N* = 50 each) that were sampled from D8 in April of 2018. All males were made by parthenogenetically reproducing females and are therefore genetically identical to their male-producing mothers. Thus, when parthenogenetically reproducing females vary in their propensity for male production, male and female pools will differ in allele frequency at regions of the genome associated with male production, allowing for the identification of candidate loci.

Bulk-segregant analysis of the pooled sequencing data (Magwene et al. 2011; Schlötterer et al. 2014) identified 14 putative QTL associated with male production (fig. 5*E*). We used the identification and numbering of QTL in the pooled sequencing for the remainder of our analysis. Three of these QTL overlapped with peaks found for male production in the A×C F1 hybrid mapping (QTLs on chromosomes 2, 5, and 12), whereas one of the same QTL (chr. 2) plus one additional QTL (chr. 13) overlapped with peaks for ephippial fill rate. The overlap between the pooled sequencing and A×C F1 hybrid mapping is only significant for ephippial fill rate (*p_perm_* = 0.01, supplementary fig. S13, Supplementary Material online). However, given the relatively small sample size for the two different experiments, any overlap is reassuring and suggests that at least some peaks are likely true positives. Next, we asked whether the sign of the allelic effects at the 14 QTL identified via pooled sequencing is concordant with differences in male production between superclones A, C, and their F1 offspring. If the QTL identified via Pool-Seq are enriched for true positives, we expect a greater frequency of male-producing alleles (*male^+^*) in superclone C, and a greater frequency of male-limiting alleles (*male*^−^) in superclone A. Consistent with this prediction, we observe that superclone C contains slightly more *male^+^* alleles than superclone A (*χ*^2^ = 4.86, df = 2, *P* = 0.087, fig. 5*F*). Notably, superclone C is homozygous at five putative QTL for the *male^+^* allele, whereas superclone A is homozygous for the *male^+^* at one QTL; superclone A is homozygous for the *male*^−^ allele at two QTL, whereas superclone C is not homozygous for the male-limiting allele at any QTL.

We also examined the relationship between male-production rate in the F1s and genotype, as polarized by the allelic effect inferred from the Pool-Seq QTL. Four of the 14 QTL had a significant relationship between genotype and male production at *P* < 0.05 (two remained significant after Bonferroni correction), and of those four, three (including the two significant after correction) exhibited the expected relationship of greater male production in clones that were homozygous for the *male^+^* genotype (supplementary fig. S14, Supplementary Material online). We also observed a significant positive correlation between male-production rate and the total number of *male^+^* alleles (fig. 5*C*) combined across both A×C and C×C crosses (binomial GLM: *χ*^2^ = 113.14, df = 1, *P* = 5 × 10^−26^) and within the A×C (*χ*^2^ = 18.87, df = 1, *P* = 1 × 10^−5^) or C×C cross (*χ*^2^ = 8.2, df = 1, *P* = 0.004). Taken together, the QTL experiments provide support for multiple QTL associated with variation in male production, although the presence of a single, main-effect locus cannot be entirely discounted.

### The Ecological and Evolutionary Dynamics of Male-Production QTL

We hypothesized a positive correlation between the frequency of the *male^+^* alleles and degree of ephemerality among ponds, as we expected male production to be under stronger selection with greater ephemerality as sexual reproduction is more frequently enforced. We examined the allele frequency distribution of male-production QTL among ponds and calculated the expected frequency of the *male^+^* alleles by summing over the observed pond-level frequencies of each clonal lineage. In general, we did not observe a consistent relationship between *male^+^* allele frequency and the degree of pond ephemerality across all QTL (supplementary fig. S15, Supplementary Material online), although there are some QTL that show striking patterns of differentiation consistent with our prediction (see below, *QTL_12_ is the best…*).

We next investigated the evolutionary history of the QTL associated with male production. We asked if alleles underlying these QTL are highly divergent, perhaps representing either recent introgression or old, balanced polymorphisms. Alternatively, young alleles may represent the product of rapid allelic turnover. To estimate allele age, we calculated the time to the most recent common ancestor (TMRCA), in generations, at every polymorphism genome-wide using the individuals from the Dorset ponds (fig. 5*G*). Of the 14 QTL identified via Pool-Seq, 11 are younger than average but not exceptionally young. The relatively young age of the QTL (one-sample proportion test with null of 50%, *χ*^2^ = 3.5, df = 1, two-tailed *P* = 0.06) suggests these alleles may turn over rapidly. Only three QTL were older than the median genome-wide age, including the best candidate QTL on chromosome 10.

### RNA-seq Identifies Candidate Genes Affecting Male Production

We next sought to identify candidate genes affecting male production between superclones A and C using RNA-seq on whole, adult females. We first document abundant gene expression variation between the superclones (supplementary fig. S16, Supplementary Material online). Principal component 1 clearly separates A and C (supplementary fig. S13*A*, Supplementary Material online; *t* = −6.6, *P* = 0.0022) and explains ∼60% of the variation in gene expression genome-wide. The magnitude of differential expression between these clones is similar to levels of differential expression previously documented between clonal lineages in other studies (Chowdhury et al. 2015; Huylmans et al. 2016). There are 12 genes falling within 50 kb of five of the QTL peaks identified by Pool-Seq that are differentially expressed (fig. 5*H*; Bonferroni corrected *P-*value < 0.05), and we consider these as candidate loci.

### QTL_12_ is the Best Candidate Locus for Male Production

**Fig. 6.**
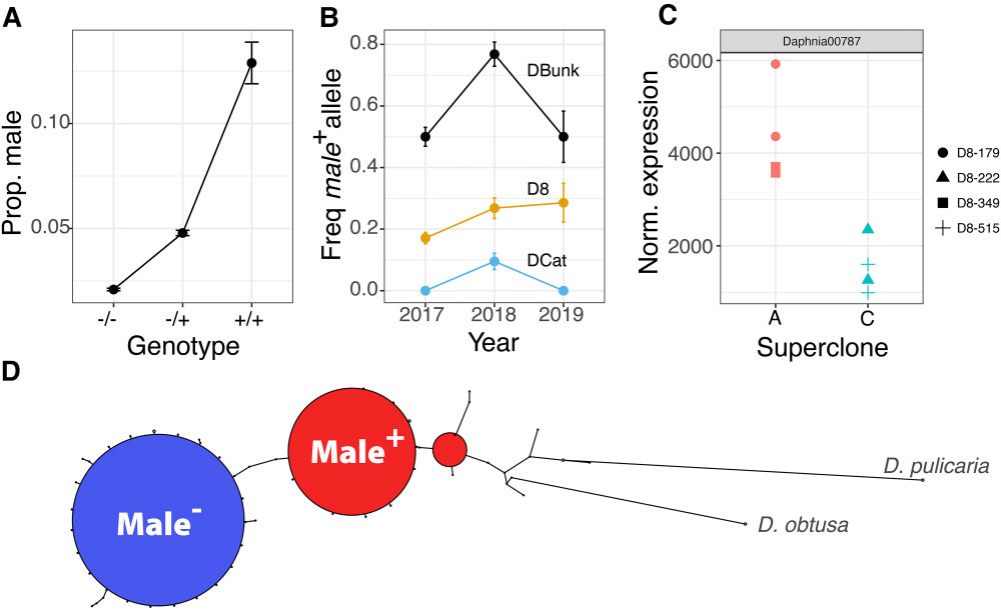
Attributes of QTL_12_. (*A*) The proportion of male offspring versus dosage of the male+ alleles for the F1 offspring of the A×C and C×C cross, with the sign of allelic effect calculated from the Pool-Seq data. (*B*) Overall frequency of the QTL_12_  *male^+^* allele among ponds across sample years. (*C*) Expression of *Daphnia00787*, one of the genes within QTL_12_ in A and C isofemale lines. The *y*-axis represents gene expression, normalized for library size. (*D*) A haplotype spanning network plot of *Daphnia00787* with *D. pulicaria* and *D. obtusa* as outgroups. The *male*^−^ allele and *male^+^* allele are labeled.

QTL_12_, located on chromosome 10 (fig. 5*E*), is of particular interest because of the confluence of multiple lines of evidence. QTL_12_ has the strongest signal in the A×C F1 hybrid mapping for male production (fig. 5*D*) and lines up with a QTL identified in the pooled sequencing mapping (fig. 5*E*). Superclone A is inferred to be homozygous for the *male*^−^ allele, and superclone C is inferred to be heterozygous *male^+^*/*male*^−^ (fig. 5*F*). Among the F1 A×C and C×C offspring, we find a strong association between genotype and male production that goes in the expected direction (binomial GLM: *χ*^2^ = 13.468, df = 2, *P* = 0.0011; fig. 6*A* and supplementary fig. S13, Supplementary Material online). QTL_12_ is strongly differentiated between DCat, D8, and DBunk in all 3 sampling years (fig. 6*B*). Notably, the frequency of the *male^+^* allele among populations varies in the expected manner, with the highest frequency in the most ephemeral pond, DBunk, and the lowest frequency in the most permanent pond, DCat (fig. 6*B*). The *male*^−^ is closely related to the *male^+^* allele (fig. 6*D*) and, consistent with our predictions, the *male*^−^ is derived.

There are four genes close to the peak of QTL_12_ that are differentially expressed (figs. 5*H* and 6*C*; supplementary fig. S16, Supplementary Material online). These four genes are among the most differentially expressed, genome-wide (top 7%). These genes are immediately adjacent to each other (supplementary fig. S16, Supplementary Material online), and are consistently down-regulated in C compared with A. It is likely that the correlated signal of differential expression among these four genes is a consequence of unannotated UTRs associated with one gene, *Daphnia00787*, overlapping with the open reading frames of the adjacent three genes (supplementary fig. S16, Supplementary Material online) and our use of unstranded RNA-seq of superclones A and C. *Daphnia00787* is the most plausible candidate gene of the four (see Discussion). *Daphnia00787* was found to be orthologous to a Tox-SGS domain-containing protein in North American *D. pulex* (blastp; coverage = 100%, identity = 89.4%).

## Discussion

Herein, we studied the genomic basis and evolutionary dynamics of reproductive investment in a facultative parthenogen, *D. pulex*. We characterized how differential investment in reproductive mode by coexisting clones affects the temporal dynamics of the genetic structure of *D. pulex* in a series of ponds that vary in ephemerality. Our work highlights the dynamic turnover of population structure through time in a facultatively sexual species and suggests that quantitative genetic variation in life-history traits can arise de novo within species and can undergo rapid evolutionary turnover.

### Diversity and Genetic Structure through Time

Our basic understanding of *Daphnia* biology suggests that clonal diversity should be strongly correlated with the degree of ephemerality, as more permanent ponds enable a longer asexual growing season and thus an increased duration of clonal selection (De Meester et al. 2006; Ortells et al. 2006). Our results are consistent with this prediction (fig. 2) in that clonal diversity was lowest in the permanent pond, DCat, and highest in the ephemeral pond, DBunk. Although previous studies have documented similar impacts of ephemerality on clonal diversity in Daphnia (Ruvinsky et al. 1986; Carvalho and Crisp 1987; De Meester and Vanoverbeke 1999; Hamrová et al. 2011), these studies used markers such as allozymes and microsatellites which cannot conclusively identify unique genotypes. Further, our results also demonstrate that reduced clonal diversity can impact the genetic structure of populations by leading to a greater occurrence of inbreeding via intraclone and full-sib mating (fig. 3).

Although clonal diversity and mating dynamics varied among the ponds, average within-individual heterozygosity did not (supplementary fig. S6, Supplementary Material online). There are several possible explanations for the consistently high levels of diversity despite the consanguineous pedigree that we observe. On the one hand, higher than expected levels of diversity could arise through neutral processes. For instance, neutral simulations that incorporated cyclical parthenogenesis showed that an excess of heterozygotes could be a consequence of drift early in the growing season (Vanoverbeke and De Meester 2010). Diversity could also be maintained at high levels through migration and the subsequent reduction of inbreeding depression (Ebert et al. 2002), although any migrant would face significant competition from the established *D. pulex* population (De Meester et al. 2002). On the other hand, high levels of genetic diversity could be maintained via selective mechanisms. Haag and Ebert (2007) found that heterozygosity increased following clonal selection in an experimental *D. magna* system, potentially due to heterosis caused by associative overdominance (Ohta 1971). Fluctuations in environmental factors over the course of the growing season via microspatial variation in selection pressures or biotic interactions (Wolinska et al. 2006; Duffy and Sivars-Becker 2007) could also contribute to the long-term persistence of variation.

We also provide clear evidence that clonal selection acts over short time periods, consistent with previous reports in Daphnia (Spaak 1996; De Meester and Vanoverbeke 1999; Vanoverbeke and De Meester 2010; Yin et al. 2010, 2012) and other facultatively sexual species (Ortells et al. 2006; Rhomberg et al. 2011). Although we do not have time-series data to directly observe clonal selection, our pedigree in D8 (fig. 3) suggests that clonal selection occurred and that the rise in frequency of a limited number of clones was rapid. Alternatively, priority effects caused by the timing of ephippial hatching are another explanation for the limited clonal diversity. Regardless, we were able to identify most of the clones involved in sexual reproduction in D8—despite sampling only a small portion of the total population (21–117 out of hundreds of thousands)—indicating an effective population size that was orders of magnitude lower than the census population size. A striking example is that D8 in 2018 was dominated by F1 hybrids between only two superclones, suggesting that by the time sexual reproduction occurred in 2017, those two superclones had almost entirely taken over the population.

### Genetic Variation and Potential Mechanisms of Male Limitation

Another striking finding was that even when clonal diversity in the intermediate pond, D8, was reduced to two dominant clones, genetic diversity for investment in sexual reproduction was maintained; in particular, variation in male production. Variation in male production is frequently found in populations of *Daphnia* (Innes and Dunbrack 1993; Tessier and Cáceres 2004; Fitzsimmons and Innes 2006; Galimov et al. 2011; Roulin et al. 2013, 2015). The loss of male production in facultatively sexual organisms likely confers a fitness advantage via higher female-production rates and is expected to quickly rise in frequency once it arises in a population (Charlesworth and Ganders 1979). Loss of male production is a common adaptive process in plants (gynodioecy) and is often affected by selfish, maternally inherited cytoplasmic elements and nuclear restorers (Taylor et al. 2001; Delph 2009). In Daphnia, loss of male production has not been linked to any cytoplasmic element, and it is generally believed that variation in male production is caused by the nuclear genome (Roulin et al. 2013; Reisser et al. 2017; Ye et al. 2019). At least for North American *D. pulex*, nonmale production is a result of adaptive introgression from the sister taxa *D. pulicaria* (Ye et al. 2019).

The evolutionary origin and genetic determinants of male limitation in the Dorset populations are distinct from loss of male production in the North American system. First, our results suggest male limitation in the Dorset populations is a quantitative trait, in contrast to a single large-effect dominant allele found in North America (Innes and Dunbrack 1993; Ye et al. 2019). The candidate gene underlying the major effect QTL identified in North American *D. pulex* (Ye et al. 2019) maps to chromosome 1 at positions 8,156,029–8,159,968 in the European *D. pulex* genome. This region is far from any putative QTL related to male production in the Dorset populations (fig. 5), supporting an independent evolutionary origin and mechanism. Further, the loss of male production in North American *D. pulex* is associated with a loss of ability to respond to juvenile hormone (Ye et al. 2019), whereas Dorset clones retain the ability to respond to this cue, regardless of male-production phenotype. Although hybridization between closely related taxa has been hypothesized to be a major driver of the evolution of reproductive modes (Lynch 1984) and is the driver of the loss of male production in North American *D. pulex* (Ye et al. 2019), we do not observe any evidence of introgression or hybridization between English *D. pulex* populations and English *D. pulicaria* or *D. obtusa* (supplementary fig. S8, Supplementary Material online). These results indicate that quantitative variation in male production in the Dorset populations is likely caused by multiple loci which are the result of alleles arising and segregating within this species and are thus inconsistent with the destabilizing hybridization hypothesis (Lynch 1984).

The best candidate gene that we identify could plausibly affect aspects of male production. This candidate gene, *Daphnia00787*, is a large (4,179 amino acids), intronless gene (supplementary fig. S16, Supplementary Material online), orthologous to a toxin-like protein (*Tox-SGS*; Zhang et al. 2012). This gene is a member of a gene family with orthologs only found in *Daphnia*, several blood-feeding insects and, notably, intracellular microbial symbionts including *Wolbachia*, *Cardinium*, and *Rickettsiella* (Baião et al. 2021). These cytoplasmically inherited symbionts influence sex ratios in a variety of arthropods (Jiggins et al. 2001; Rosenwald et al. 2020; Lv et al. 2021) and it is therefore plausible that this toxin gene could be involved in sex-allocation in the *Daphnia* system that we identified here.

### The Generation and Persistence Time of Variation in Reproductive Investment

Maintenance of genetic variation for male production in the Dorset populations may be facilitated by fluctuating selection. Low-male producing clones are likely to establish and spread during periods of favorable conditions, due to the short-term advantage of asexual reproduction of female offspring (Nunney 1989; Wolinska and Lively 2008; Serra and Snell 2009; Stelzer 2011; Gibson et al. 2017). This predicted increase in the frequency of low-male producing clones or clones with an overall lower propensity for sexual reproduction has been observed in *Daphnia* in the lab (Innes and Singleton 2000) and in rotifers in the field (Carmona et al. 2009), supporting a cost of male production. Similarly, we observed the male-producing superclone C to be at lower frequency in 2017 (22%) than the male-limited superclone A (67%) the year before the hatching of A×C F1s (fig. 3). We also did not observe any C×C offspring in 2018 despite ample selfing in experimental superclone C mesocosms in the lab, suggesting that superclone C may have been at an even lower frequency at the point in time ephippia were produced. In the summer of 2017, D8 dried and in 2018 it consisted almost entirely of A×C F1 hybrids. This pattern demonstrates that although male-producing clones may suffer a short-term fitness disadvantage during the asexual growing season, ultimately they can achieve high fitness if they are the primary source of males and dormancy remains linked to sexual reproduction. As a consequence, seasonal fluctuations in selection for asexual versus sexual reproduction in facultative sexuals may facilitate the maintenance of genetic polymorphism in sexual investment.

Maintenance of genetic polymorphism via seasonal fluctuations will also be influenced by the duration of those fluctuations. In populations where favorable conditions persist for an extended period of time, male-limited clones may come to fully dominate the population. Consistent with this idea, the dominant superclone in our permanent population, DCat, was found to be a low-male producer, similar to superclone A in D8 (supplementary fig. S16, Supplementary Material online). On the other hand, when the asexual growing season is short or unpredictable, clones with low sexual investment will not have sufficient time to reap the short-term benefits of asexual reproduction and clones with high sexual investment will be favored (Galimov et al. 2011; Smith and Snell 2012; Franch-Gras et al. 2017; Tarazona et al. 2017). Thus, we expect to see variation in selection for male production across populations, with male-producing alleles favored in more ephemeral environments. Our best candidate locus, QTL_12_, did exhibit this pattern; however, there was no consistent correlation across QTL between ephemerality and frequency of the male-producing allele. This lack of pattern could be due to multiple causes. First, our sample size is low, with only three ponds, giving us reduced power to pick up any overall pattern between ephemerality and male production. Second, two of our ponds, DBunk and D8 are intermittently connected in the late fall and early winter when the water level is highest, and thus frequent migration could prevent differential local adaptation between these two ponds (similar to Roulin et al. 2015). Additional work is needed to determine the role, if any, that variation in selection due to variation in ephemerality plays in the maintenance of genetic variation for male production in this system.

The fluctuating seasonal dynamics of these populations are also likely to influence rates of allelic turnover and thus the evolutionary dynamics of male production. Although a few of the putative QTL associated with male production were older than the genome-wide average, including QTL_12_, the majority were younger, suggesting these QTL are predominantly the product of rapid allelic turnover rather than the maintenance of old, stable polymorphisms. Rapid allelic turnover at QTL associated with variation in male production is consistent with theoretical models that predict faster rates of fixation and loss at functional polymorphisms subject to temporally varying selection (Bürger and Gimelfarb 2002; Cvijović et al. 2015).

### Conclusion

We characterized the genetic structure of a *D. pulex* metapopulation across an ephemerality gradient and identified naturally segregating genetic variants affecting a key fitness trait, male production. It is likely that substantial genetic variation in other fitness related traits also segregates among these clones, and that such variation is maintained via selective processes. Due to the fact that the natural genetic structure of these populations resembles a multiparental QTL mapping panel, we envision that future work on the genetic basis of phenotypic variation in these, and other similarly structured populations, will yield valuable insight into the evolutionary forces that maintain genetic variation within populations.

## Materials and Methods

### Characterization and Sampling of Ponds

All field work and sampling were carried out in the Dorset region of the southern United Kingdom (fig. 1*A*). The focal metapopulation consisted of three geographically close (20–30 m) small ponds located in the Kilwood Nature Reserve (50.642483, −2.091652). Two of these ponds, DBunk and D8, are intermittently connected, with DBunk downstream of D8; there are no obvious streambeds connecting DCat to either DBunk or D8. An additional pond, D10, located ∼11 km away in the Higher Hyde Heath Nature Reserve (50.709379, −2.206421) was also part of this study and serves as a more distant population reference.

To characterize changes in water level and clonal dynamics of the focal populations, we visited the four ponds at multiple time points across multiple years (2016–2019; supplementary tables S1 and S3, Supplementary Material online). The water level of ponds was noted and in 2019, time lapse cameras were installed at D8 and DBunk to monitor changes in water level. Tows were taken from each pond that still contained sufficient water to sample the *Daphnia* population. For the 2016 and 2017 time points, live individuals were shipped back to the lab and clonal lineages were established for later sequencing and phenotyping. For sampling points in 2018 and 2019, *Daphnia* were primarily fixed in ethanol in the field.

### Reference Genome

Initial reference genome assembly was carried out using 10× Chromium sequencing. The *D. pulex* clone (D8.4A) used for the reference genome was sampled in 2012 from the D8 pond (fig. 1*A*) and maintained in the lab asexually since collection. Several hundred individuals from the clone were fed Sephadex G-25 superfine (cross-linked dextran gel) beads for 48 h in order to clear their guts and minimize algal reads in downstream sequencing. After the 48 h, 70 mg (wet weight) of *Daphnia* were flash frozen in liquid nitrogen and shipped to HudsonAlpha for high molecular weight DNA extraction, 10× Chromium library preparation, and whole-genome sequencing on a single lane of HiSeqX. Assembly was carried out using Supernova-1.1.5 (supernova mkfastq, supernova run, supernova mkoutput) (Weisenfeld et al. 2017), with the input downsampled to 200 million reads and the output fasta made using the *pseudohap* style. Only resulting scaffolds over 1 kb were kept for downstream analysis. The 10× Chromium reads were deposited at NCBI’s SRA: SRR14333786.

Scaffolding of the reference genome was achieved via Chicago and Hi-C sequencing (Dovetail Genomics, Scotts Valley, CA, USA). Several hundred individuals from the same reference genome clone D8.4A were exposed to Sephadex G-25 beads and antibiotics (50 mg/l each of streptomycin, tetracycline, and ampicillin) for 48 h to minimize algal and bacterial contamination, and then flash frozen in liquid nitrogen, and sent to Dovetail for Chicago and Hi-C sequencing. The input de novo 10× assembly, Chicago library reads, and Dovetail HiC library reads were used as input data for HiRise, a software pipeline designed specifically for using proximity ligation data to scaffold genome assemblies (Putnam et al. 2016). An iterative analysis was conducted. First, Chicago library sequences were aligned to the draft input assembly using a modified SNAP read mapper (http://snap.cs.berkeley.edu). The separations of Chicago read pairs mapped within draft scaffolds were analyzed by HiRise to produce a likelihood model for genomic distance between read pairs, and the model was used to identify and break putative misjoins, to score prospective joins, and make joins above a threshold. After aligning and scaffolding Chicago data, Dovetail HiC library sequences were aligned and scaffolded following the same method. The resulting assembly consisted of 9,202 scaffolds. Due to the fact that the *Daphnia* for the initial 10× Chromium assembly were not treated with antibiotics, many of these scaffolds are likely to be microbial in origin. By blasting the scaffolds against previously published North American *D. pulex* reference genomes (TCO and PA42) (Colbourne et al. 2011; Ye et al. 2017), 768 scaffolds were determined to be *Daphnia* in origin (positive hit to both genomes). Of these 768 scaffolds, 13 were over 50 KB and 12 were over 5 MB. These 12 largest scaffolds were determined to correspond to the 12 chromosomes in the previously published *D. pulex* genomes (supplementary table S5, Supplementary Material online; Colbourne et al. 2011; Ye et al. 2017).

To improve the reference genome assembly, the reference genome clone was also sequenced on a Nanopore Minion flow cell. Around 100 individuals of the reference genome clone were exposed to antibiotics and Sephadex G-25 beads for 48 h. DNA was extracted using Beckman-Coulter’s Agencourt DNAdvance kit. The library was constructed using Nanopore’s basic Ligation Sequencing Kit (SQK-LSK-109) and run on a single Minion SpotON flow cell (R9.4) for 48 h using default parameters. Reads were basecalled using Albacore (version 2.3.3, Oxford Nanopore Technologies, Oxford, UK). The resulting MinIon reads (deposited at NCBI’s SRA: SRR14567272) were combined with the original 10× Chromium Illumina reads (filtered to only include reads that mapped to the good *Daphnia* contigs, thus excluding microbial reads) to make a hybrid genome assembly using MaSuRCA (Zimin et al. 2013). To filter the 10× Chromium reads, the reads were mapped to the HiC scaffolded genome, keeping only alignments where both read pairs overlapped with the 768 scaffolds determined to be Daphnia in origin. The paired reads were then output to two fasta files using samtools fastq. This hybrid genome assembly along with the filtered 10× Chromium paired-end Illumina reads were then used to close gaps of *N*s in the Dovetail HiC genome using GMCloser (v1.6.2, Kosugi et al. 2015) with the -c option. The final genome size was 127,796,161 bp and 98.8% of that was contained in the 12 largest contigs. The genome sequence has been deposited at DDBJ/ENA/GenBank under the accession JAHCQT000000000. Additional information regarding aspects of experimental design for this and following experiments can be found in supplementary table S2, Supplementary Material online.

Gene prediction was performed on the *D. pulex* genome using MAKER (v2.31.10; Holt and Yandell 2011) in an iterative manner. First, ab initio gene prediction was performed by the programs SNAP (v2006-07-28; Korf 2004) and Augustus (v3.2.3; Stanke and Morgenstern 2005; Stanke et al. 2006) using a multi-tissue *D. pulex* transcriptome (see *RNA-seq library preparation,* below) assembly obtained using StringTie with default parameters (v2.0; Pertea et al. 2015) and a protein database that combined the UniProt/Swiss-Prot and NCBI nonredundant database of reviewed arthropod proteins as well as the curated protein set for a related species, *D. magna* (http://arthropods.eugenes.org/EvidentialGene/daphnia/daphnia_magna_new/, accessed 5/6/2019). A total of three iterative runs of SNAP were used to refine the gene models. A final round of gene prediction was then performed using MAKER to produce the final gene set with an annotation edit distance cutoff of 0.5 (Holt and Yandell 2011). This gene set consists of 13,455 genes that encode 17,930 proteins. Genome annotation quality was assessed by BUSCO analysis (Seppey et al. 2019) using the conserved core set of arthropod genes (∼94.2% BUSCO score). Using the set of complete predicted *D. pulex* protein sequences, we used a functional annotation pipeline previously described (Suryamohan et al. 2020) to assign putative functions to each predicted protein. The functional annotation information can be found in supplementary table S4, Supplementary Material online.

Repetitive elements were identified for masking in downstream analysis using RepeatModeler (Smit and Hubley 2008) and RepeatMasker (Smit et al. 2013). First, the RepeatModeler BuildDatabase option was used to construct a database from the D8.4A reference genome file. Next, RepeatModeler was run on the newly constructed database file. The resulting repeat library was then concatenated with the repeat family library constructed from the North American *D. pulex* genome, PA42 (Ye et al. 2017). RepeatMasker was run on the D8.4A reference genome using this combined repeat family library. Both RepeatModeler and RepeatMasker were run using the *ncbi engine* option.

We generated a list of regions that are masked from future analysis. First, we identified regions of the genome with very high or very low read depth, when mapping the Illumina reads generated for 10× linked-read sequencing back to the reference genome. We identified sites with the 1.5% lowest coverage and sites with 7.5% highest coverage sites and removed these sites from future analysis. In addition, we flagged stretches of *N*s, the first and last 500 bp of sequence for each chromosome, and repetitive regions identified with RepeatMasker.

### Sequencing

Individual males, females, and pools of individuals were sequenced from samples collected across 4 years (supplementary table S3, Supplementary Material online). For the 2016 and 2017 samples, individuals were sampled from lines that had been established in the lab. For each of these lines, multiple individuals were exposed to antibiotics (streptomycin, tetracycline, and ampicillin, 50 mg/l of each) and fed Sephadex G-25 beads. Five to 10 individuals from each line were used for DNA extraction (Agencourt DNAdvance kit, Beckman-Coulter). Individuals were homogenized using metal beads and a bead beater before DNA extraction. RNA was also removed using RNase followed by an additional bead cleanup. DNA was quantified using the broad-range Quant-iT dsDNA kit (ThermoFisher Scientific) and normalized to 1 or 2 ng/μl before library construction. Full genome libraries were constructed using a scaled down Nextera protocol (Baym et al. 2015). Libraries were size-selected for fragments ranging from 450 to 550 bp using a Blue Pippin and quality checked using a BioAnalyzer. For 2018 and 2019 samples, DNA was extracted from single individuals (females with parthenogenetic eggs or males) fixed in ethanol in the field. Normalization and library construction were carried out in the same way as for 2016 and 2017 samples, except the samples were not RNAse treated due to low DNA concentration.

Pools of males and females were made up of individuals sampled from D8 on 04/29/2018 and fixed in ethanol in the field. There were two pools of 50 females and two pools of 35 males. Libraries were constructed using NEB’s NEBNext Ultra II DNA Library Prep Kit for Illumina.

Three additional individual *D. pulex* from two more northern populations in the UK (W1 and W6), 15 *D. obtusa* (14 from DBunk, and one from a nearby pond also in the Kilwood Nature Reserve, DBarb), and 5 *D. pulicaria* sampled from a single population in the UK (53.581442, −0.312748) were also sequenced to serve as geographically and taxonomically distant references.

Samples from 2016 (*N* = 52) were sequenced on two lanes of HiSeqX. Samples from 2017 were sequenced in three batches (*N* = 94, 94, and 190; 3, 3, and 6 lanes of HiSeqX respectively), with some samples repeated between batches if initial read depth from early sequencing runs was too low. Initial samples from 2018 (*N* = 94) were run on a single lane of NovaSeq 6000 S4 300. Samples from 2019 (*N* = 61), additional samples from 2018 (*N* = 33), and the male and female pooled samples from 2018 were pooled and sequenced on a single lane of NovaSeq 6000 S4 300. Fastq samples are available on NCBI’s SRA (PRJNA725506). See supplementary table S3, Supplementary Material online for accession numbers for individual samples. Pooled samples: SAMN19227056–SAMN19227059.

### Mapping, SNP Calling, and Annotation

For each of the individual samples, Nextera adaptor sequences were removed using *Trimmomatic* v0.36 (Bolger et al. 2014). Next, overlapping reads were merged using *PEAR* v0.9.11 (Zhang et al. 2014). Assembled and unassembled reads were separately mapped to the European *D. pulex* genome using *bwa mem* (Li 2013). The entire reference genome was used for mapping, but only reads that mapped to *Daphnia* scaffolds were retained for further analysis. Reads that were primary alignments and had mapping quality scores (QS) > 20 were output into bam files. For samples that were sequenced across multiple lanes, samtools (Li et al. 2009) was used to merge bam files from each lane, and then PCR duplicates were removed using *MarkDuplicates* function of *Picard* (Anon 2019). GATK (v4.0; McKenna et al. 2010; Poplin et al. 2018) was used to call SNPs. gVCFs for each sample were made using GATK’s HaplotypeCaller tool, and then a single VCF (variant call format) was made using GATK’s GenotypeGVCF tool. We performed functional annotation of the VCF file using *SnpEff* (v4.3t, Cingolani et al. 2012) utilizing the gene predictions described above.

### SNP Filtering

First, we filtered out SNPs that were within 10 base pairs of indels; indels were also removed from analysis. SNPs were hard filtered using GATK’s recommendations for organisms with no reference SNP panel. Specifically, the following filters were first set using GATK’s VariantFiltration tool (QualByDepth (QD) < 2, FisherStrand (FS) > 60, RMSMappingQuality (MQ) < 40, MappingQualityRankSumTest (MQRankSum)  < −12.5, and ReadPosRankSumTest (ReadPosRankSum) < −8), and SNPs that did not pass this filter were removed using GATK’s SelectVariants tool. Individual genotype calls with low GQ (GQ < 10) were then set as missing data. The resulting VCF consisted of 3,719,919 SNPs.

SNPs were removed if they fell in regions that were flagged as being near stretches of *N*s or the ends of chromosomes, as well as in areas of high or low read depth (described above in the Reference Genome section). Together, this filtration resulted in a loss of 651,900 SNPS, leaving 3,068,019.

SNPs were filtered from regions flagged by Repeat Masker (described above in the Reference Genome section). Filtering SNPs from flagged repeat regions resulted in a loss of an additional 320,003 SNPs. Triallelic SNPs were also removed, resulting in a further loss of 81,624 SNPs. SNPs were then filtered based on read depth across all samples, with the bottom and top 5% of SNPs being dropped, which resulted in a loss of 266,515 SNPs, leaving 2,399,653 SNPs. This set of filtered SNPs included SNPs polymorphic within *D. pulex*, as well as SNPs that are fixed within *D. pulex*, but are either different between *D. pulex* and one of the outgroups, or polymorphic within one of the outgroups. This SNP set will be referred to as the “total filtered SNP set.”

For analyses restricted to *D. pulex* samples, a second filtered SNPs set was used. For this SNP set, only SNPs that were polymorphic within the *D. pulex* samples and had been genotyped in at least half the *D. pulex* samples were retained. This SNP set, consisting of 510,805 SNPs, will be referred to as the “variable *pulex* SNP set.” Finally, this variable *pulex* SNP set was also LD pruned using the snpgdsLDpruning function in SNPRelate (Zheng et al. 2012), with a minor allele frequency (MAF) cutoff of 0.001, a missing rate of 0.15, a maximum window size of 500 bp, and an LD threshold of 0.1. This LD pruned SNP set had 150,455 SNPs and is referred to as the “LD pruned, variable pulex SNP set.”

### Clonal Assignment

Individual clones were assigned to clonal lineages using genome-wide estimates of IBS calculated using the *snpgdsIBS* function in *SNPRelate* (Zheng et al. 2012, 2017), excluding singleton SNPs (1/2*N*) and SNPs with a missing rate >15%, and using the LD pruned, variable *pulex* SNP set. Based on the distribution of pairwise IBS (supplementary fig. S3, Supplementary Material online), an initial cutoff of 0.965 IBS was chosen as a threshold, above which two individuals are considered to belong to the same clonal lineage (asexually related to one another). Note that these IBS values are calculated based on the table of polymorphic sites, and not the whole genome and thus the denominator for these statistics is the number of polymorphisms and not the genome size. Assuming a genome size of 120 Mb and that the number of polymorphisms used for this analysis is ∼500,000, maximum divergence (Dxy) between individuals of the same superclone is 1.44 × 10^−^ Clonal diversity for each pond at each time point was calculated using Shannon’s diversity index in the R package vegan (Oksanen et al. 2020).

### Genealogical Relationships Between Isolates

To further investigate patterns of relatedness, we calculated IBS_0_ and kinship coefficients using the program KING (Manichaikul et al. 2010). KING was run using the “kinship” command on the variable *pulex* SNP set (non-LD pruned), with the input data filtered to include SNPs with a MAF cutoff of 0.05. The distribution of IBS_0_ and Kinship mostly confirmed the clonal lineage assignment based on the IBS threshold. However, there were three individuals that appeared as outliers within their clonal lineages in terms of Kinship. Specifically, the Kinship values were lower than that expected for clonal relationships, but higher than expected for outcrossing parent–offspring relationships (e.g., supplementary fig. S3*C* and *D*, Supplementary Material online). In one case, based on patterns of SNP segregation, the outlier individual was identified as the parent of the lineage, with the lineage resulting from an intraclone cross. In three other examples of selfing, the chosen IBS threshold did separate the parent from the offspring lineage. In two other cases, the relationship was not as clear, and the outlier individuals were classified instead as close relatives of their respective clonal lineages. All three outlier individuals were removed from their respective clonal lineages.

The distribution of pairwise IBS_0_ and Kinship calculated in KING were also used to identify parent–offspring relationships and construct a pedigree. Similar to clonal relationships, parent–offspring pairwise comparisons (whether due to selfing or outcrossing events) are expected to have an IBS_0_ of roughly 0, with Kinship values being highest for clonal comparisons, followed by selfed parent–offspring, and then outcrossed parent–offspring. Graphing IBS_0_ and Kinship resulted in clear clustering of these three types of relationships (supplementray fig. S3*B*, Supplementary Material online), which were used to identify parent–offspring relationships and construct a pedigree for the three focal ponds across sample years. Selfed parent–offspring relationships were confirmed by checking segregation patterns of parental heterozygous sites in the offspring. Segregation patterns of parental SNPs were also used to check outcrossed parent–offspring relationships where possible. For example, A×C F1 hybrids were confirmed to be heterozygous for all SNPs that are fixed differences between A and C.

### Diversity Estimates and Runs of Homozygosity

ROHan v1.0 (Renaud et al. 2019) was used to estimate genome-wide heterozygosity for each individual and to identify runs of homozygosity. For this analysis, we used the 12 major chromosomes and a transition/transversion ratio of 0.81 (Flynn et al. 2017).

### Mutation Accumulation

To study patterns of mutation accumulation in the wild, we identified eight wild-caught individuals from four superclones (range of individuals per superclone = 9–127). We randomly selected eight individuals per superclone and estimated *p*_N_/*p*_S_ for new mutations (those segregating at <25%) and for shared variants (those segregating at ∼50%). We generated confidence intervals on *p*_N_/*p*_S_ by bootstrap resampling (*n* = 100).

### Sliding Window IBS

A sliding window analysis of IBS was used to examine patterns of divergence and relatedness between the two dominant superclones in D8 (A and C) and determine if these patterns were unique relative to patterns of divergence among other *D. pulex* clonal lineages found in the focal ponds. Pairwise IBS among all individuals (including outgroups) was calculated using the snpgdsIBS function in SNPRelate (Zheng et al. 2012) in overlapping windows of 250,000 bp with a 10,000 bp step size, using the total filtered SNP set. Singleton SNPs (1/2*N*) were removed from the analysis as were SNPs with a missing rate >15%. Windows with <1,000 SNPs were removed from the analysis. Individual clones with a median read depth of <5 were also removed from the analysis. Next, for each window, mean pairwise IBS was calculated for each pair of clonal lineages. For example, when calculating mean IBS between the A and C superclones, IBS was averaged across all pairwise comparisons between A and C individuals. For comparisons involving clonal lineages that had only a single representative, the original pairwise IBS value was used, as a mean could not be calculated for a single value. Then, for each window, IBS for A versus C was divided by the calculated IBS for each pair of comparison clonal lineages to obtain an IBS ratio. Finally, a mean, genome-wide similarity ratio was calculated by averaging across windows for each clonal lineage comparison. Values <1 indicate the comparison clones are more similar than A versus C, whereas values >1 indicate the comparison clones are more divergent than A versus C. A versus C IBS was compared (1) to IBS between other clonal lineages within D8, (2) to IBS between clonal lineages within DCat and within DBunk, (3) to IBS between clones from different focal ponds (between DCat, D8, and DBunk), and (4) to IBS between focal pond clonal lineages and clonal lineages from three geographically distant *D. pulex* ponds (D10, W1, and W6).

### Phasing and Imputation of Wild-Caught Individuals

We identified one representative per superclone among the 169 wild-caught *D. pulex* (supplementary table S3, Supplementary Material online) by selecting the clone with the highest read depth. We performed read-backed phasing using *whatshap* (v1.1; Patterson et al. 2015), and performed population based phasing and imputation using *shapeit* (v4; Delaneau et al. 2019). We generated consensus genotypes for two outgroups, *D. pulicaria* and *D. obtusa*, at sites that were polymorphic in the phased-imputed dataset.

### Hybridization and Introgression Analysis

We tested for potential hybridization and introgression between *D. pulex* and its outgroups in two ways. First, we used *Dsuite* (Malinsky et al. 2021) to calculate *f_4_* and *f_4_*-ratio statistics (Patterson et al. 2012) using the phased, imputed dataset. Next, we used the *snmf* function in *LEA* v3.12 (Frichot and François 2015) to estimate individual admixture components using the phased, imputed dataset. For the *snmf* analysis, we varied *K* from 1 to 20 and performed 30 replicate runs. We selected the value of *K* with the lowest cross-entropy score as the optimal number of clusters (*k* = 8).

### Phenotypic Differentiation between A and C

To test for phenotypic differences between the two dominant superclones in D8 (A and C), two field-collected (2017) isofemale lines of each superclone were expanded in mesocosms (A: D8–179, D8–349, C: D8–222, D8–515). We tracked population dynamics over the course of 8 weeks. First, each isofemale line was propagated in two tanks containing 15 l of artificial hard water (ASTM; Standard 2007) with seaweed extract (marinure; Wilfrid Smith Std., Northans, UK) in a Percival incubator set at 18 °C under long days (16L:8D). Sixty females just before first reproduction were isolated from the mesocosms once in the expansion phase, and used to establish the experimental mesocosms.

Eight experimental mesocosms were established, two for each isofemale line (two field isolates × two clonal lineages × two replicates = eight tanks). Each experimental mesocosm consisted of a fish tank containing 15 l of ASTM with marinure placed in the same Percival incubator set at 18 °C under long days (16L:8D). For the first 5 weeks, each tank was fed 95,000 cells/ml *Chlorella vulgaris* Monday/Wednesday/Friday (M/W/F), and then were switched to 142,000 cells/ml algae for weeks 6–7. Tanks were checked weekly after initial establishment for 7 weeks. To check the tanks, each tank was stirred up well to evenly suspend all individuals, and then a liter of media was removed from each tank and sieved to isolate the *Daphnia*. These *Daphnia* were fixed in ethanol and later sorted into the following demographic classes: females with parthenogenic eggs, females with ephippia, females with neither parthenogenic eggs or ephippia, female neonates, juvenile males, and adult males.

At each sample time point, all loose ephippia were also removed from each tank. All ephippia were counted and a subset was dissected to determine fill rate. The number of ephippia dissected varied widely depending on the total number of loose ephippia produced (*N* = 1–70, mean = 22). These fill rates were used to estimate total sexual embryo production (estimated fill rate × total number of ephippia = estimated sexual embryo production). Total population size was calculated as the total number of individuals in the sample times 15.

Linear mixed-effects models in *R* were used to test for significant differences between the two superclones regarding total population size, proportion asexual females, proportion sexual females, male production, and sexual embryo production (lme4; Bates et al. 2015). Differences in population size and sexual embryo production were tested using lmer (gaussian error model), with isofemale line and week included as random effects. To test for a significant effect of superclone, ANOVA was used to test for a significant improvement in fit when including or not including superclone as a fixed effect in the model. Differences in proportion asexual females, sexual females, and males were analyzed using glmer with a binomial error model weighted by population size and isofemale line and week included as random effects. A significant effect of superclone was again determined by using ANOVA to test for a significant improvement in fit when including or excluding superclone in the model.

The same four isofemale lines used for the mesocosm experiments (A: D8–179, D8–349, C: D8–222, D8–515) were exposed to MF to determine their response in regards to male production. Individual neonates released within 24 h were placed in 50 ml jars in ASTM with marinure. The second clutch neonates from these individuals were used for the experiment. Individual neonates were placed in 50 ml jars in ASTM with marinure and checked M/W/F. When the first clutch was observed, the female was moved to a new jar and the neonates were counted and scored as male or female. Females in the MF treatment were placed in jars containing 50 ml of ASTM plus marinure and MF at a concentration of 400 nM. Females in the control treatment were placed in jars containing 50 ml of ASTM plus marinure and ethanol to control for any side effects of the ethanol in which the MF was resuspended. Females were moved to new jars with corresponding media (control or MF) every M/W/F and checked for neonates, which were counted and scored as male or female. Jars were followed for 4–6 clutches, although in a few cases individuals died before the fourth clutch. Altogether 83 neonates were included in the experiment (*N* = 7–14 per trmt/field isolate combination), 71 of which survived to at least the third clutch (5–11 per trmt/field isolate combination). Throughout the experiment, jars were fed 200,000 cells ml^−1^  *C. vulgaris* M/W/F and were maintained under 16:8 light:dark conditions at 20 °C.

For analysis, the proportion of male neonates from the first clutch was used as a pretreatment estimate of male production. The summed proportion of males from clutches three and higher were used for posttreatment estimates of male production. Clutch 2 was not included in the analysis as this clutch was not evenly exposed to MF across jars. Since jars were checked M/W/F and females were moved into treatment after the first clutch was observed, the exposure of the second clutch to MF was variable, but the third and higher clutches were fully exposed for all females. An overall significant effect of MF exposure was tested in two ways. First, a significant interaction between treatment (MF vs. control) and pre/postexposure was tested using glmer, with treatment and pre/postexposure included as fixed effects and maternal lineage as a random effect, and comparing the fit of the model with and without the interaction between treatment and exposure. ANOVA was used to test for a significant improvement in fit when including the interaction term. Second, restricting the analysis to postexposure samples, a significant effect of MF on proportion male was tested using glmer, with maternal lineage as a random effect, and again using ANOVA to compare the fit of the model with and without the inclusion of treatment as a fixed effect. Finally, to test whether the two superclones responded differently to MF, the data were again restricted to postexposure samples. Proportion male was modeled using glmer, with treatment as a fixed effect and maternal lineage as a random effect with and without superclone as a fixed effect. ANOVA was used to test for a significant improvement in fit when including superclone in the model. All analyses used a binomial error model weighted by number of neonates.

### Generation and Phenotyping of F1 A×C and C×C Offspring

Patterns of segregation in ephippial fill rate and male production were examined in both selfed C and A×C F1 hybrid clonal lineages. Selfed C and A×C F1 clonal lineages were obtained from the mesocosms, described above. Ephippia isolated from the mesocosms were hatched and clonal lineages were maintained in the lab for several months before the experiment. Selfed C lineages (*N* = 24) came from C tanks as well as A/C mixed tanks. A×C F1 hybrid lineages (*N* = 26) came either from A/C mixed mesocosms or were isolated from the field. No selfed A or selfed C clonal lineages were isolated from the field. The four A/C superclone field isofemale lines (A: D8–179, D8–349; B: D8–222, D8–515) were also included. One liter jars were established for each clonal lineage and maintained at high densities (average of 50 reproductively mature females, 125 individuals total) and maintained for multiple weeks. The jars contained ASTM plus marinure, were fed 200,000 cells ml^−1^  *C. vulgaris* M/W/F, and were maintained under 16:8 h light:dark conditions at 20 °C. The artificial hard water was changed via pouring over every 2 weeks (leaving a small amount of original water in the jar), maintaining high population sizes, and all loose ephippia were collected, counted, and dissected to score fill rate. Most clonal lineages were maintained in two replicates, whereas a few had either one or three replicates, and the A/C isofemale lines had five replicates. Jars were checked between 2 and 16 times, with a mean of 6. At the end of the experiment, the jars were sieved and the *Daphnia* were fixed in ethanol. *Daphnia* were sorted into demographic classes and counted, and the proportion of males was calculated. An effect of clone on male production within the A×C and within the C×C hybrids was modeled using a binomial glm in *R* with proportion male as the response variable, clone as the explanatory variable, and total population size as a weighting factor. Significance was tested using the *χ*^2^ test option in the ANOVA ( ) function in *R*.

### Phasing and Imputation of A×C and C×C F1 Offspring

We performed transmission-based phasing on 50 unique lab- and field-generated F1s using *rabbit* v3.2 (Zheng et al. 2018). All field-caught individuals (*n* = 10) were inferred as A×C F1s based on patterns of segregation described above (see Genealogical Relationships Between Isolates). There were 16 lab-generated A×C and 24 lab-generated C×C genomes sequenced. First, we built consensus genotypes for the parental genomes (A and C) and selected the 5,000 informative markers between these strains. Next, we used the *magicImpute* function to impute missing data in the offspring and to phase the parental genomes. Finally, we used *magicReconstruct* to generate phased genomes of the recombinant offspring using the Viterbi decoding algorithm.

### QTL Mapping Using F1s

Using the phased and imputed genomes of recombinant individuals, we identified the 5,436 unique polymorphisms. These SNPs represent “tag” SNPs and are in perfect linkage with the remaining 115,399 informative markers identified between A and C. We used this set of markers for QTL analysis, and later propagated the signals of association at the tag-SNPs to the entirety of the linkage block.

We performed QTL mapping using *lme4qtl* (Ziyatdinov et al. 2018) and generated the additive genetic relatedness matrix (GRM) using the *A.mat* function in *rrblup* (v4.6.1, Endelman 2011). We generated a new GRM for each of the SNPs we tested in order to avoid proximal contamination (Yang et al. 2014). We performed 100 permutations to generate an empirical false discovery rate (Churchill and Doerge 1994), which we calculated for each chromosome separately.

We calculated linkage-disequilbium between tag-SNPs underlying QTL identified here, and among the remaining tag-SNPs using the *snpgdsLDMat* function in SNPRelate.

### Bioinformatics and Analysis of Pool-Seq Samples

Pool-Seq samples of males and females bearing parthenogenic offspring were generated, as described above. We first estimated allele frequencies in the Pool-Seq samples using the ASEReadCounter function of GATK (McKenna et al. 2010) at the ∼500,000 SNPs that are part of the “variable *pulex* SNP set.” Raw coverage ranged from 81 to 350 and effective coverage ranged from 45 to 76 (supplementary table S6, Supplementary Material online).

Previous sequencing of 2018 D8 clones in the lab had indicated that in 2018 the D8 population predominantly consisted of A×C F1 hybrids (fig. 3). We confirmed that the Pool-Seq samples were primarily F1 hybrids between A and C in two ways. First, we calculated the proportion of sites segregating in the Pool-Seq data that are also polymorphic between A and C. Superclones A and C are different genotypes at 98% of sites with a MAF > 5% in the Pool-Seq data. Second, we partitioned allele frequencies in the Pool-Seq samples based on the nine F1 genotypic combinations between A and C. We show that observed allele frequencies are close to the expected value (supplementary fig. S12, Supplementary Material online). Because our Pool-Seq samples are composed of genetically diverse F1s we are justified in performing bulk-segregant analysis as typically applied to experimental crosses.

We used the *G′* test (Magwene et al. 2011) as implemented in *QTLSeqR* (Mansfeld and Grumet 2018) to perform bulk-segregant analysis. First, we took the reference and alternate allele counts calculated by *ASEReadCounter* and downsampled them proportional to the effective read depth (Kolaczkowski et al. 2011; Feder et al. 2012), which factors out the double binomial sampling that occurs during Pool-Seq. We calculated the *G′* statistic filtering for sites with MAF > 15%, minimum total read depth of 20, minimum sample depth of 20, and a window size of 250,000 bp. We calculated *G′* pairing one of each replicate pool of females and males. We corrected for multiple testing using the Benjamini–Hochberg FDR (false discovery rate) method (Benjamini and Hochberg 1995).

### QTL Overlap Analysis

To test whether QTL identified from lab-based F1 mapping and QTL identified from wild-caught Pool-Seq overlap, we performed an enrichment test. First, we defined the QTL boundaries from the F1 mapping by performing one-dimensional position-based clustering of SNPs that surpassed the significance threshold. Clustering was performed using the R-package *Ckmeans.1d.dp* (Wang and Song 2011), and QTL boundaries were defined as the minimum and maximum positions of each cluster. Pool-Seq QTL boundaries were identified by the *QTLSeqR* package (Mansfeld and Grumet 2018). To test if QTL identified by each method overlap, we used the R package *regioneR* (Gel et al. 2016). We calculated the null distribution of the overlap statistic (*z*, see supplementary fig. S13, Supplementary Material online) using the permuted F1 QTL mapping analysis.RNA-seq Library Preparation

For genome annotation, we conducted RNA sequencing on a *D. pulex* clone (D8.6A) that was collected from the same pond as the reference genome (D8) in 2012 and maintained as isofemale lineages in artificial hard water (ASTM) with seaweed extract (marinure) under standard conditions in the lab. Before RNA extraction, this clone was maintained under standard conditions for three generations and fed daily. Animals were sieved, rinsed with ASTM and snap-frozen in liquid nitrogen at late embryonic stage (E4; 286 pooled embryos per sample), and early and late first instar (I1.1early, I1.1late; 143 pooled animals per sample each). For sample preparation, 896 µl diluted methanol was added to each sample, and samples were homogenized for 2 × 10 s bursts at 6,400 rpm (Precellys). Samples were placed on dry ice and 300 µl sample was transferred into an RNAse free tube and flash frozen in liquid nitrogen. RNA from samples was subsequently extracted using the RNAdvance Tissue kit (Agencourt) following manufacturer’s instructions, including the optional DNase treatment step. RNA integrity was verified using an Agilent Tapestation 2200 with High Sensitivity RNA screentapes (all RIN > 6.2). RNA libraries were produced using the Biomek F×P (Beckman-Coulter A31842) with NEBNext Ultra II Directional RNA Library Prep Kit (New England Biolab E7420L) and NEBnext Multiplex Oligos for Illumina Dual Index Primers (New England Biolabs E7600S), using provided protocols and 500 ng of total RNA. Constructed libraries were assessed for quality using the Tapestation 2200 with High Sensitivity D1000 DNA screentape. Multiplex library clustering and sequencing was performed upon the HiSeq2500 (four lanes rapid run 2× 100 bp) by BGI Copenhagen. Fastq files are available at NCBI’s SRA (PRJNA727995).

We also conducted RNA sequencing on two A clones and two C clones, using two biological replicates of each. We snap-froze 20 females for each biological replicate in liquid nitrogen and stored samples at −80 °C. We extracted RNA using the RNAdvance Tissue kit (Agencourt) following manufacturer’s instructions, including the optional DNase treatment step. RNA integrity was verified using an Agilent Bioanalyzer (all RIN (RNA integrity number) > 6.2). We isolated poly-adenylated RNA from 1 µg of total RNA using the NEBNext Poly(A) mRNA Magnetic Isolation Module (NEB #E7490) and constructed sequencing libraries with the NEBNext Ultra II RNA Library Prep kit (NEB #E7770) using dual indices (NEB #E7600). We quantified libraries by Bioanalyzer and with a Quant-iT kit (Thermo Fisher) and pooled the eight libraries in equimolar concentrations. The pooled libraries were sequenced in one lane of Illumina HiSeq X with 150 bp paired-end reads. The resulting fastq files were deposited in NCBI’s SRA: SRR14572418-SRR14572425.

### RNA-seq Bioinformatics

NA-seq reads were mapped to the reference genome using STAR v2.7.2b (Dobin et al. 2013) using the following parameters: –outFilterMatchNmin 0 –outSJfilterReads Unique –outSJfilterCountUniqueMin 20 1 1 1 –alignIntronMax 25000 –outFilterMismatchNmax 20 –outFilterIntronStrands RemoveInconsistentStrands –sjdbOverhang 100. Quality control of mapped reads and postmapping processing was performed using QoRTS v1.3.6 (Hartley and Mullikin 2015). Differential expression analysis between superclones A and C was performed using DESeq2 (Love et al. 2014).

### Haplotype Network Analysis

We estimated a haplotype network by using whole haplotype sequences extracted from the phased VCF containing one representative per superclone and including sequences from outgroup taxa. Diploid haplotypes for the *Daphnia00787* gene were extracted using *bcftools* (Li et al. 2009) (consensus function) and the reference genome of *D. pulex* sliced to the region corresponding to Scaffold “2217_HRSCAF_2652” from 5,191,562 to 5,204,101 bp. Before analysis, haplotype sequences were reversed and complemented using the string unix functions *rev* and *tr* ACGT TGCA, respectively. Individual haplotypes were concatenated into a single FASTA file and loaded into R using functions from *ape* (Paradis et al. 2004). Network analysis was done using the package *pegas* (Paradis 2010).

### Allele Age Analysis

Estimates of allele age (TMRCA) were done using the GEVA program (Albers and McVean 2020) and using the following population genetic parameters: recombination rate = 1.60e−8, mutation rate = 5.69e−09, and effective population size = 862,000. GEVA was implemented on the phased VCF containing one representative per superclone excluding all outgroups. Before analysis, we filtered SNPs with mAF < 0.01.

### Daphnia00787 Orthology

Orthology of *Daphnia00787* was assessed by blasting the reference sequence of the gene against the NCBI database using the *blastp* algorithm with default settings.

### Statistical Analysis and Plotting

Statistical analysis was performed using R version 3.5–4.0.5 (R Core Development Team). The following packages were used for general analysis and plotting: *ggplot2* (Wickham 2016), *cowplot* (Wilke 2019), *patchwork* (Pedersen 2020), *viridis* (Garnier et al. 2021), *leaflet* (Cheng et al. 2021), *data*.*table* (Dowle et al. 2021), *foreach* (Wallig, Analytics, et al. 2020), *doMC* (Wallig, Microsoft, et al. 2020), *SeqArray* (Zheng et al. 2017).

## Supplementary Material

Supplementary data are available at *Molecular Biology and Evolution* online.

## Supplementary Material

msac121_Supplementary_Data

## Data Availability

All scripts and code used for data analysis and plotting are available at https://github.com/alanbergland/DaphniaFromDorset. All sequencing reads have been deposited at NCBI’s Sequence Read Archive (Bioproject # PRJNA725506). The reference genome has been deposited at DDBJ/ENA/GenBank under the accession JAHCQT000000000. Raw phenotype data, the VCF file, and associated files used for SNP analysis, as well as supplementary table S1, Supplementary Material online with information on all individuals sequenced are available on Dryad: https://datadryad.org/stash/share/BEppQ_0gXxGRZgsuVmHwwD9T1-0XU4r2YXvpSRlblDc. Maps for figure 1*A* are available from (left panel: https://d-maps.com/carte.php?num_car=2554&lang=en; middle panel: map taken from OpenStreetMaps; right panel: © Crown copyright and database rights 2021 Ordnance Survey).

## References

[msac121-B1] Albers PK, McVean G. 2020. Dating genomic variants and shared ancestry in population-scale sequencing data. PLoS Biol. 18:e3000586. 31951611 10.1371/journal.pbio.3000586 PMC6992231

[msac121-B2] Baião GC, Janice J, Galinou M, Klasson L. 2021. Comparative genomics reveals factors associated with phenotypic expression of *Wolbachia*. Genome Biol Evol. 13:evab111. 34003269 10.1093/gbe/evab111 PMC8290115

[msac121-B3] Barbuti R, Mautner S, Carnevale G, Milazzo P, Rama A, Sturmbauer C. 2012. Population dynamics with a mixed type of sexual and asexual reproduction in a fluctuating environment. BMC Evol Biol. 12:49. 22489797 10.1186/1471-2148-12-49 PMC3353185

[msac121-B4] Bates D, Mächler M, Bolker B, Walker S. 2015. Fitting linear mixed-effects models using lme4. J Stat Softw. 67:1–48.

[msac121-B5] Baym M, Kryazhimskiy S, Lieberman TD, Chung H, Desai MM, Kishony R. 2015. Inexpensive multiplexed library preparation for megabase-sized genomes. PLoS One 10:e0128036. 26000737 10.1371/journal.pone.0128036 PMC4441430

[msac121-B6] Benjamini Y, Hochberg Y. 1995. Controlling the false discovery rate: a practical and powerful approach to multiple testing. J R Stat Soc Ser B Methodol. 57:289–300.

[msac121-B7] Bolger AM, Lohse M, Usadel B. 2014. Trimmomatic: a flexible trimmer for Illumina sequence data. Bioinformatics 30:2114–2120. 24695404 10.1093/bioinformatics/btu170 PMC4103590

[msac121-B8] Brendonck L, De Meester L. 2003. Egg banks in freshwater zooplankton: evolutionary and ecological archives in the sediment. Hydrobiologia 491:65–84.

[msac121-B9] Bürger R, Gimelfarb A. 2002. Fluctuating environments and the role of mutation in maintaining quantitative genetic variation. Genet Res. 80:31–46. 12448856 10.1017/s0016672302005682

[msac121-B10] Burke NW, Bonduriansky R. 2017. Sexual conflict, facultative asexuality, and the true paradox of sex. Trends Ecol Evol. 32:646–652. 28651895 10.1016/j.tree.2017.06.002

[msac121-B11] Cáceres CE . 1997. Dormancy in Invertebrates. Invertebr Biol. 116:371–383.

[msac121-B12] Cáceres CE, Soluk DA. 2002. Blowing in the wind: a field test of overland dispersal and colonization by aquatic invertebrates. Oecologia 131:402–408. 28547712 10.1007/s00442-002-0897-5

[msac121-B13] Carmona MJ, Dimas-Flores N, García-Roger EM, Serra M. 2009. Selection of low investment in sex in a cyclically parthenogenetic rotifer. J Evol Biol. 22:1975–1983. 19678864 10.1111/j.1420-9101.2009.01811.x

[msac121-B14] Carvalho GR, Crisp DJ. 1987. The clonal ecology of *Daphnia magna* (Crustacea:Cladocera): I. temporal changes in the clonal structure of a natural population. J Anim Ecol. 56:453–468.

[msac121-B15] Charlesworth D, Ganders FR. 1979. The population genetics of gynodioecy with cytoplasmic-genic male-sterility. Heredity 43:213–218.

[msac121-B16] Cheng J, Karambelkar B, Xie Y, Wickham H, Russell K, Johnson K, Schloerke B, jQuery Foundation and contributors, Agafonkin V, CloudMade, et al 2021. leaflet: Create Interactive Web Maps with the JavaScript “Leaflet” Library. Available from: https://CRAN.R-project.org/package=leaflet.

[msac121-B17] Chowdhury PR, Frisch D, Becker D, Lopez JA, Weider LJ, Colbourne JK, Jeyasingh PD. 2015. Differential transcriptomic responses of ancient and modern *Daphnia* genotypes to phosphorus supply. Mol Ecol. 24:123–135. 25410011 10.1111/mec.13009

[msac121-B18] Churchill GA, Doerge RW. 1994. Empirical threshold values for quantitative trait mapping. Genetics 138:963–971. 7851788 10.1093/genetics/138.3.963 PMC1206241

[msac121-B19] Cingolani P, Platts A, Wang LL, Coon M, Nguyen T, Wang L, Land SJ, Lu X, Ruden DM. 2012. A program for annotating and predicting the effects of single nucleotide polymorphisms, SnpEff: SNPs in the genome of *Drosophila melanogaster* strain w1118; iso-2; iso-3. Fly (Austin) 6:80–92. 22728672 10.4161/fly.19695 PMC3679285

[msac121-B20] Colbourne JK, Pfrender ME, Gilbert D, Thomas WK, Tucker A, Oakley TH, Tokishita S, Aerts A, Arnold GJ, Basu MK, et al 2011. The ecoresponsive genome of *Daphnia pulex*. Science 331:555–561. 21292972 10.1126/science.1197761 PMC3529199

[msac121-B21] Cvijović I, Good BH, Jerison ER, Desai MM. 2015. Fate of a mutation in a fluctuating environment. Proc Natl Acad Sci U S A. 112:E5021–E5028. 26305937 10.1073/pnas.1505406112 PMC4568713

[msac121-B22] Dedryver C-A, Hullé M, Le Gallic J-F, Caillaud MC, Simon J-C. 2001. Coexistence in space and time of sexual and asexual populations of the cereal aphid *Sitobion avenae*. Oecologia 128:379–388. 24549907 10.1007/s004420100674

[msac121-B23] Delaneau O, Zagury J-F, Robinson MR, Marchini JL, Dermitzakis ET. 2019. Accurate, scalable and integrative haplotype estimation. Nat Commun. 10:5436. 31780650 10.1038/s41467-019-13225-y PMC6882857

[msac121-B24] Delph LF . 2009. Sex allocation: evolution to and from dioecy. Curr Biol. 19:R249–R251. 19321140 10.1016/j.cub.2009.01.048

[msac121-B25] De Meester L, Gómez A, Okamura B, Schwenk K. 2002. The Monopolization Hypothesis and the dispersal–gene flow paradox in aquatic organisms. Acta Oecol. 23:121–135.

[msac121-B26] De Meester LD, Vanoverbeke J. 1999. An uncoupling of male and sexual egg production leads to reduced inbreeding in the cyclical parthenogen *Daphnia*. Proc R Soc Lond B Biol Sci. 266:2471–2477. 10.1098/rspb.1999.0948 PMC1690476 10693817

[msac121-B27] De Meester L, Vanoverbeke J, De Gelas K, Ortells R, Spaak P. 2006. Genetic structure of cyclic parthenogenetic zooplankton populations—a conceptual framework. Arch Für Hydrobiol. 167:217–244.

[msac121-B28] Dobin A, Davis CA, Schlesinger F, Drenkow J, Zaleski C, Jha S, Batut P, Chaisson M, Gingeras TR. 2013. STAR: ultrafast universal RNA-seq aligner. Bioinformatics 29:15–21. 23104886 10.1093/bioinformatics/bts635 PMC3530905

[msac121-B29] Dowle M, Srinivasan A, Gorecki J, Chirico M, Stetsenko P, Short T, Lianoglou S, Antonyan E, Bonsch M, Parsonage H, et al 2021. data.table: Extension of “data.frame.” Available from: https://CRAN.R-project.org/package=data.table.

[msac121-B30] Duffy MA, Sivars-Becker L. 2007. Rapid evolution and ecological host–parasite dynamics. Ecol Lett. 10:44–53. 17204116 10.1111/j.1461-0248.2006.00995.x

[msac121-B31] Ebert D . 2005. Ecology, epidemiology, and evolution of parasitism in Daphnia. National Center for Biotechnology Information (US).

[msac121-B32] Ebert D, Haag C, Kirkpatrick M, Riek M, Hottinger JW, Pajunen VI. 2002. A selective advantage to immigrant genes in a *Daphnia* metapopulation. Science 295:485–488. 11799241 10.1126/science.1067485

[msac121-B33] Endelman JB . 2011. Ridge regression and other kernels for genomic selection with R package rrBLUP. Plant Genome 4:250–255.

[msac121-B34] Feder AF, Petrov DA, Bergland AO. 2012. LDx: estimation of linkage disequilibrium from high-throughput pooled resequencing data. PLoS One 7:e48588. 23152785 10.1371/journal.pone.0048588 PMC3494690

[msac121-B35] Fitzsimmons JM, Innes DJ. 2006. Inter-genotype variation in reproductive response to crowding among *Daphnia pulex*. Hydrobiologia 568:187–205.

[msac121-B36] Flynn JM, Chain FJJ, Schoen DJ, Cristescu ME. 2017. Spontaneous mutation accumulation in *Daphnia pulex* in selection-free vs. competitive environments. Mol Biol Evol. 34:160–173. 27777284 10.1093/molbev/msw234

[msac121-B37] Franch-Gras L, García-Roger EM, Serra M, José Carmona M. 2017. Adaptation in response to environmental unpredictability. Proc R Soc B Biol Sci. 284:20170427. 10.1098/rspb.2017.0427 PMC5740265 29212717

[msac121-B38] Frichot E, François O. 2015. LEA: an R package for landscape and ecological association studies. Methods Ecol Evol. 6:925–929.

[msac121-B39] Galimov Y, Walser B, Haag CR. 2011. Frequency and inheritance of non-male producing clones in *Daphnia magna*: evolution towards sex specialization in a cyclical parthenogen? J Evol Biol. 24:1572–1583. 21599772 10.1111/j.1420-9101.2011.02288.x

[msac121-B40] Garnier S, Ross N, Rudis B, Sciaini M, Camargo AP, Scherer C. 2021. viridis: Default color maps from “matplotlib.” Available from: https://CRAN.R-project.org/package=viridis.

[msac121-B41] Gel B, Díez-Villanueva A, Serra E, Buschbeck M, Peinado MA, Malinverni R. 2016. regioneR: an R/Bioconductor package for the association analysis of genomic regions based on permutation tests. Bioinformatics 32:289–291. 26424858 10.1093/bioinformatics/btv562 PMC4708104

[msac121-B42] Gerber N, Booksmythe I, Kokko H. 2018. Sex allocation theory for facultatively sexual organisms inhabiting seasonal environments: the importance of bet hedging. Am Nat. 192:155–170. 30016165 10.1086/697727

[msac121-B43] Gerber N, Kokko H. 2018. Abandoning the ship using sex, dispersal or dormancy: multiple escape routes from challenging conditions. Philos Trans R Soc Lond B Biol Sci. 373:20170424. 30150222 10.1098/rstb.2017.0424 PMC6125733

[msac121-B44] Gerber N, Kokko H, Ebert D, Booksmythe I. 2018. *Daphnia* invest in sexual reproduction when its relative costs are reduced. Proc R Soc B Biol Sci. 285:20172176. 10.1098/rspb.2017.2176 PMC5805931 29343596

[msac121-B45] Gibson AK, Delph LF, Lively CM. 2017. The two-fold cost of sex: experimental evidence from a natural system. Evol Lett. 1:6–15. 30233811 10.1002/evl3.1 PMC6089407

[msac121-B46] Gillespie JH, Turelli M. 1989. Genotype–environment interactions and the maintenance of polygenic variation. Genetics 121:129–138. 17246488 10.1093/genetics/121.1.129 PMC1203595

[msac121-B47] Haag CR, Ebert D. 2007. Genotypic selection in *Daphnia* populations consisting of inbred sibships. J Evol Biol. 20:881–891. 17465899 10.1111/j.1420-9101.2007.01313.x

[msac121-B48] Hadany L, Otto SP. 2007. The evolution of condition-dependent sex in the face of high costs. Genetics 176:1713–1727. 17483405 10.1534/genetics.107.074203 PMC1931531

[msac121-B49] Hairston NG, Munns WR. 1984. The timing of copepod diapause as an evolutionarily stable strategy. Am Nat. 123:733–751.

[msac121-B50] Hairston NG, Van Brunt RA. 1994. Diapause dynamics of two diaptomid copepod species in a large lake. Hydrobiologia 292:209–218.

[msac121-B51] Hamrová E, Mergeay J, Petrusek A. 2011. Strong differences in the clonal variation of two *Daphnia* species from mountain lakes affected by overwintering strategy. BMC Evol Biol. 11:231. 21824417 10.1186/1471-2148-11-231 PMC3161014

[msac121-B52] Hartfield M . 2016. Evolutionary genetic consequences of facultative sex and outcrossing. J Evol Biol. 29:5–22. 26431643 10.1111/jeb.12770

[msac121-B53] Hartley SW, Mullikin JC. 2015. QoRTs: a comprehensive toolset for quality control and data processing of RNA-Seq experiments. BMC Bioinform. 16:224. 10.1186/s12859-015-0670-5 PMC4506620 26187896

[msac121-B54] Hebert PDN, Finston TL. 2001. Macrogeographic patterns of breeding system diversity in the *Daphnia pulex* group from the United States and Mexico. Heredity 87:153–161. 11703505 10.1046/j.1365-2540.2001.00885.x

[msac121-B55] Hedrick PW . 1976. Genetic variation in a heterogeneous environment. II. Temporal heterogeneity and directional selection. Genetics 84:145–157. 992363 10.1093/genetics/84.1.145 PMC1213561

[msac121-B56] Holt C, Yandell M. 2011. MAKER2: an annotation pipeline and genome-database management tool for second-generation genome projects. BMC Bioinform. 12:491. 10.1186/1471-2105-12-491 PMC3280279 22192575

[msac121-B57] Hörandl E . 2006. The complex causality of geographical parthenogenesis. New Phytol. 171:525–538. 16866956 10.1111/j.1469-8137.2006.01769.x

[msac121-B58] Huylmans AK, López Ezquerra A, Parsch J, Cordellier M. 2016. De novo transcriptome assembly and sex-biased gene expression in the cyclical parthenogenetic *Daphnia galeata*. Genome Biol Evol. 8:3120–3139. 27604882 10.1093/gbe/evw221 PMC5174735

[msac121-B59] Innes DJ . 1997. Sexual reproduction of *Daphnia pulex* in a temporary habitat. Oecologia 111:53–60. 28307505 10.1007/s004420050207

[msac121-B60] Innes DJ, Dunbrack RL. 1993. Sex allocation variation in *Daphnia pulex*. J Evol Biol. 6:559–575.

[msac121-B61] Innes DJ, Hebert PDN. 1988. The origin and genetic basis of obligate parthenogenesis in *Daphnia pulex*. Evolution 42:1024–1035. 28581165 10.1111/j.1558-5646.1988.tb02521.x

[msac121-B62] Innes DJ, Singleton DR. 2000. Variation in allocation to sexual and asexual reproduction among clones of cyclically parthenogenetic *Daphnia pulex* (Crustacea: Cladocera). Biol J Linn Soc. 71:771–787.

[msac121-B63] Jiggins FM, Bentley JK, Majerus MEN, Hurst GDD. 2001. How many species are infected with *Wolbachia*? Cryptic sex ratio distorters revealed to be common by intensive sampling. Proc R Soc Lond B Biol Sci. 268:1123–1126. 10.1098/rspb.2001.1632 PMC1088716 11375098

[msac121-B64] Kokko H . 2020. When synchrony makes the best of both worlds even better: how well do we really understand facultative sex? Am Nat. 195:380–392. 32017623 10.1086/706812

[msac121-B65] Kolaczkowski B, Kern AD, Holloway AK, Begun DJ. 2011. Genomic differentiation between temperate and tropical Australian populations of *Drosophila melanogaster*. Genetics 187:245–260. 21059887 10.1534/genetics.110.123059 PMC3018305

[msac121-B66] Korf I . 2004. Gene finding in novel genomes. BMC Bioinform. 5:59. 10.1186/1471-2105-5-59 PMC421630 15144565

[msac121-B67] Kosugi S, Hirakawa H, Tabata S. 2015. GMcloser: closing gaps in assemblies accurately with a likelihood-based selection of contig or long-read alignments. Bioinformatics 31:3733–3741. 26261222 10.1093/bioinformatics/btv465

[msac121-B68] LeBlanc GA, Medlock EK. 2015. Males on demand: the environmental-neuro-endocrine control of male sex determination in daphnids. FEBS J. 282:4080–4093. 26237283 10.1111/febs.13393

[msac121-B69] Li H . 2013. Aligning sequence reads, clone sequences and assembly contigs with BWA-MEM. ArXiv13033997 Q-Bio [Internet]. Available from: https://arxiv.org/abs/1303.3997.

[msac121-B70] Li H, Handsaker B, Wysoker A, Fennell T, Ruan J, Homer N, Marth G, Abecasis G, Durbin R, 1000 Genome Project Data Processing Subgroup. 2009. The sequence alignment/map format and SAMtools. Bioinforma Oxf Engl. 25:2078–2079. 10.1093/bioinformatics/btp352 PMC2723002 19505943

[msac121-B71] R Core Development Team . A language and environment for statistical computing. Vienna, Austria: R Foundation for Statistical Computing.

[msac121-B72] Love MI, Huber W, Anders S. 2014. Moderated estimation of fold change and dispersion for RNA-seq data with DESeq2. Genome Biol. 15:550. 25516281 10.1186/s13059-014-0550-8 PMC4302049

[msac121-B73] Lv N, Peng J, Chen X-Y, Guo C-F, Sang W, Wang X-M, Ahmed MZ, Xu Y-Y, Qiu B-L. 2021. Antagonistic interaction between male-killing and cytoplasmic incompatibility induced by *Cardinium* and *Wolbachia* in the whitefly, *Bemisia tabaci*. Insect Sci. 28:330–346. 32339445 10.1111/1744-7917.12793

[msac121-B74] Lynch M . 1984. Destabilizing hybridization, general-purpose genotypes and geographic parthenogenesis. Q Rev Biol. 59:257–290.

[msac121-B75] Lynch M, Gutenkunst R, Ackerman M, Spitze K, Ye Z, Maruki T, Jia Z. 2017. Population genomics of *Daphnia pulex*. Genetics 206:315–332. 27932545 10.1534/genetics.116.190611 PMC5419477

[msac121-B76] Lynch M, Seyfert A, Eads B, Williams E. 2008. Localization of the genetic determinants of meiosis suppression in *Daphnia pulex*. Genetics 180:317–327. 18689898 10.1534/genetics.107.084657 PMC2535684

[msac121-B77] Magwene PM, Willis JH, Kelly JK. 2011. The statistics of bulk segregant analysis using next generation sequencing. PLoS Comput Biol. 7:e1002255. 22072954 10.1371/journal.pcbi.1002255 PMC3207950

[msac121-B78] Malinsky M, Matschiner M, Svardal H. 2021. Dsuite – fast *D*-statistics and related admixture evidence from VCF files. Mol Ecol Resour. 21:584–595. 33012121 10.1111/1755-0998.13265 PMC7116594

[msac121-B79] Manichaikul A, Mychaleckyj JC, Rich SS, Daly K, Sale M, Chen W-M. 2010. Robust relationship inference in genome-wide association studies. Bioinformatics 26:2867–2873. 20926424 10.1093/bioinformatics/btq559 PMC3025716

[msac121-B80] Mansfeld BN, Grumet R. 2018. QTLseqr: an R package for bulk segregant analysis with next-generation sequencing. Plant Genome 11:180006. 10.3835/plantgenome2018.01.0006 PMC12810111 30025013

[msac121-B81] McKenna A, Hanna M, Banks E, Sivachenko A, Cibulskis K, Kernytsky A, Garimella K, Altshuler D, Gabriel S, Daly M, et al 2010. The Genome Analysis Toolkit: a MapReduce framework for analyzing next-generation DNA sequencing data. Genome Res. 20:1297–1303. 20644199 10.1101/gr.107524.110 PMC2928508

[msac121-B82] Meirmans S, Meirmans PG, Kirkendall LR. 2012. The costs of sex: facing real-world complexities. Q Rev Biol. 87:19–40. 22518931 10.1086/663945

[msac121-B83] Nunney L . 1989. The maintenance of sex by group selection. Evolution 43:245–257. 28568559 10.1111/j.1558-5646.1989.tb04225.x

[msac121-B84] Ohta T . 1971. Associative overdominance caused by linked detrimental mutations. Genet Res. 18:277–286. 5158298

[msac121-B85] Oksanen J, Blanchet FG, Friendly M, Kindt R, Legendre P, McGlinn D, Minchin PR, O’Hara RB, Simpson GL, Solymos P, et al 2020. vegan: Community Ecology Package. Available from: https://CRAN.R-project.org/package=vegan.

[msac121-B86] Ortells R, Gómez A, Serra M. 2006. Effects of duration of the planktonic phase on rotifer genetic diversity. Archiv fur Hydrobiologie 167:203–216.

[msac121-B87] Paland S, Colbourne JK, Lynch M. 2005. Evolutionary history of contagious asexuality in *Daphnia pulex*. Evolution 59:800–813. 15926690

[msac121-B88] Paradis E . 2010. pegas: an R package for population genetics with an integrated-modular approach. Bioinformatics 26:419–420. 20080509 10.1093/bioinformatics/btp696

[msac121-B89] Paradis E, Claude J, Strimmer K. 2004. APE: analyses of phylogenetics and evolution in R language. Bioinformatics 20:289–290. 14734327 10.1093/bioinformatics/btg412

[msac121-B90] Patterson M, Marschall T, Pisanti N, van Iersel L, Stougie L, Klau GW, Schönhuth A. 2015. WhatsHap: weighted haplotype assembly for future-generation sequencing reads. J Comput Biol. 22:498–509. 25658651 10.1089/cmb.2014.0157

[msac121-B91] Patterson N, Moorjani P, Luo Y, Mallick S, Rohland N, Zhan Y, Genschoreck T, Webster T, Reich D. 2012. Ancient admixture in human history. Genetics 192:1065–1093. 22960212 10.1534/genetics.112.145037 PMC3522152

[msac121-B92] Pedersen TL . 2020. Patchwork: the composer of plots. Available from: https://CRAN.R-project.org/package=patchwork.

[msac121-B93] Pertea M, Pertea GM, Antonescu CM, Chang T-C, Mendell JT, Salzberg SL. 2015. StringTie enables improved reconstruction of a transcriptome from RNA-seq reads. Nat Biotechnol. 33:290–295. 25690850 10.1038/nbt.3122 PMC4643835

[msac121-B94] Anon. 2019. Picard toolkit. Broad Institute Available from: http://broadinstitute.github.io/picard/.

[msac121-B95] Poplin R, Ruano-Rubio V, DePristo MA, Fennell TJ, Carneiro MO, Van der Auwera GA, Kling DE, Gauthier LD, Levy-Moonshine A, Roazen D, et al 2018. Scaling accurate genetic variant discovery to tens of thousands of samples. bioRxiv:201178.

[msac121-B96] Putnam NH, O’Connell BL, Stites JC, Rice BJ, Blanchette M, Calef R, Troll CJ, Fields A, Hartley PD, Sugnet CW, et al 2016. Chromosome-scale shotgun assembly using an in vitro method for long-range linkage. Genome Res. 26:342–350. 26848124 10.1101/gr.193474.115 PMC4772016

[msac121-B97] Reisser CMO, Fasel D, Hürlimann E, Dukič M, Haag-Liautard C, Thuillier V, Galimov Y, Haag CR. 2017. Transition from environmental to partial genetic sex determination in *Daphnia* through the evolution of a female-determining incipient W chromosome. Mol Biol Evol. 34:575–588. 28007974 10.1093/molbev/msw251 PMC6279290

[msac121-B98] Renaud G, Hanghøj K, Korneliussen TS, Willerslev E, Orlando L. 2019. Joint estimates of heterozygosity and runs of homozygosity for modern and ancient samples. Genetics 212:587–614. 31088861 10.1534/genetics.119.302057 PMC6614887

[msac121-B99] Rhomberg L, Joseph S, Singh R. 2011. Seasonal variation and clonal selection in cyclically parthenogenetic rose aphids (*Macrosiphum rosae*). Can J Genet Cytol. 27:224–232.

[msac121-B100] Rosenwald LC, Sitvarin MI, White JA. 2020. Endosymbiotic *Rickettsiella* causes cytoplasmic incompatibility in a spider host. Proc Biol Sci. 287:20201107. 32635864 10.1098/rspb.2020.1107 PMC7423472

[msac121-B101] Roulin AC, Mariadassou M, Hall MD, Walser J-C, Haag C, Ebert D. 2015. High genetic variation in resting-stage production in a metapopulation: is there evidence for local adaptation? Evolution 69:2747–2756. 26418426 10.1111/evo.12770

[msac121-B102] Roulin AC, Routtu J, Hall MD, Janicke T, Colson I, Haag CR, Ebert D. 2013. Local adaptation of sex induction in a facultative sexual crustacean: insights from QTL mapping and natural populations of *Daphnia magna*. Mol Ecol. 22:3567–3579. 23786714 10.1111/mec.12308

[msac121-B103] Ruvinsky AO, Perelygin AA, Lobkov YI, Belyaev DK. 1986. Factors organising and maintaining polymorphism in a cyclic parthenogenetic species: *Daphnia pulex*. Heredity 57:15–22.

[msac121-B104] Schlötterer C, Tobler R, Kofler R, Nolte V. 2014. Sequencing pools of individuals – mining genome-wide polymorphism data without big funding. Nat Rev Genet. 15:749–763. 25246196 10.1038/nrg3803

[msac121-B105] Schröder T . 2005. Diapause in monogonont rotifers. In: Herzig A Gulati RD Jersabek CD, May L, editors. Rotifera X: Rotifer Research: Trends, New Tools and Recent Advances, Proceedings of the Xth International Rotifer Symposium, held in Illmitz, Austria, 7–13 June 2003. Developments in Hydrobiology. Dordrecht: Springer Netherlands. p. 291–306.

[msac121-B106] Seppey M, Manni M, Zdobnov EM. 2019. BUSCO: assessing genome assembly and annotation completeness. Methods Mol Biol. 1962:227–245. 31020564 10.1007/978-1-4939-9173-0_14

[msac121-B107] Serra M, Snell TW. 2009. Sex loss in monogonont rotifers. In: Schön I Martens K, Dijk P, editors. Lost sex: the evolutionary biology of parthenogenesis. Dordrecht: Springer Netherlands. p. 281–294.

[msac121-B108] Simon J-C, Rispe C, Sunnucks P. 2002. Ecology and evolution of sex in aphids. Trends Ecol Evol. 17:34–39.

[msac121-B109] Smit A, Hubley R. 2008. RepeatModeler Open-1.0. Available from: http://www.repeatmasker.org.

[msac121-B110] Smit A, Hubley R, Green P. 2013. RepeatMasker Open-4.0. Available from: http://www.repeatmasker.org.

[msac121-B111] Smith HA, Snell TW. 2012. Rapid evolution of sex frequency and dormancy as hydroperiod adaptations. J Evol Biol. 25:2501–2510. 22994805 10.1111/j.1420-9101.2012.02614.x

[msac121-B112] Spaak P . 1996. Temporal changes in the genetic structure of the *Daphnia* species complex in Tjeukemeer, with evidence for backcrossing. Heredity 76:539–548.

[msac121-B113] Spencer M, Colegrave N, Schwartz SS. 2001. Hatching fraction and timing of resting stage production in seasonal environments: effects of density dependence and uncertain season length. J Evol Biol. 14:357–367.

[msac121-B114] Standard A . 2007. Standard guide for conducting acute toxicity tests on test materials with fishes, macroinvertebrates, and amphibians. West Conshohocken PA U. S. DOI 01520E0729-96 URL Www Atsm Org.

[msac121-B115] Stanke M, Morgenstern B. 2005. AUGUSTUS: a web server for gene prediction in eukaryotes that allows user-defined constraints. Nucleic Acids Res. 33:W465–467. 15980513 10.1093/nar/gki458 PMC1160219

[msac121-B116] Stanke M, Schöffmann O, Morgenstern B, Waack S. 2006. Gene prediction in eukaryotes with a generalized hidden Markov model that uses hints from external sources. BMC Bioinform. 7:62. 10.1186/1471-2105-7-62 PMC1409804 16469098

[msac121-B117] Stelzer C-P . 2011. The cost of sex and competition between cyclical and obligate parthenogenetic rotifers. Am Nat. 177:E43–E53. 21460550 10.1086/657685

[msac121-B118] Suryamohan K, Krishnankutty SP, Guillory J, Jevit M, Schröder MS, Wu M, Kuriakose B, Mathew OK, Perumal RC, Koludarov I, et al 2020. The Indian cobra reference genome and transcriptome enables comprehensive identification of venom toxins. Nat Genet. 52:106–117. 31907489 10.1038/s41588-019-0559-8 PMC8075977

[msac121-B119] Tarazona E, García-Roger EM, Carmona MJ. 2017. Experimental evolution of bet hedging in rotifer diapause traits as a response to environmental unpredictability. Oikos 126:1162–1172.

[msac121-B120] Taylor DR, Olson MS, McCauley DE. 2001. A quantitative genetic analysis of nuclear-cytoplasmic male sterility in structured populations of *Silene vulgaris*. Genetics 158:833–841. 11404344 10.1093/genetics/158.2.833 PMC1461698

[msac121-B121] Tessier AJ, Cáceres CE. 2004. Differentiation in sex investment by clones and populations of *Daphnia*. Ecol Lett. 7:695–703.

[msac121-B122] Tilquin A, Kokko H. 2016. What does the geography of parthenogenesis teach us about sex? Philos Trans R Soc Lond B Biol Sci. 371:20150538. 27619701 10.1098/rstb.2015.0538 PMC5031622

[msac121-B123] Toyota K, Miyakawa H, Hiruta C, Furuta K, Ogino Y, Shinoda T, Tatarazako N, Miyagawa S, Shaw JR, Iguchi T. 2015. Methyl farnesoate synthesis is necessary for the environmental sex determination in the water flea *Daphnia pulex*. J Insect Physiol. 80:22–30. 25721056 10.1016/j.jinsphys.2015.02.002

[msac121-B124] Toyota K, Miyakawa H, Yamaguchi K, Shigenobu S, Ogino Y, Tatarazako N, Miyagawa S, Iguchi T. 2015. NMDA receptor activation upstream of methyl farnesoate signaling for short day-induced male offspring production in the water flea, *Daphnia pulex*. BMC Genomics 16:186. 25867484 10.1186/s12864-015-1392-9 PMC4372037

[msac121-B125] Tucker AE, Ackerman MS, Eads BD, Xu S, Lynch M. 2013. Population-genomic insights into the evolutionary origin and fate of obligately asexual *Daphnia pulex*. Proc Natl Acad Sci U S A. 110:15740–15745. 23959868 10.1073/pnas.1313388110 PMC3785735

[msac121-B126] Turelli M, Barton NH. 2004. Polygenic variation maintained by balancing selection: pleiotropy, sex-dependent allelic effects and *G* × *E* interactions. Genetics 166:1053–1079. 15020487 10.1093/genetics/166.2.1053 PMC1470722

[msac121-B127] Vanoverbeke J, De Meester LD. 2010. Clonal erosion and genetic drift in cyclical parthenogens – the interplay between neutral and selective processes. J Evol Biol. 23:997–1012. 20345816 10.1111/j.1420-9101.2010.01970.x

[msac121-B128] Vorburger C, Lancaster M, Sunnucks P. 2003. Environmentally related patterns of reproductive modes in the aphid *Myzus persicae* and the predominance of two ‘superclones’ in Victoria, Australia. Mol Ecol. 12:3493–3504. 14629364 10.1046/j.1365-294x.2003.01998.x

[msac121-B129] Vorburger C, Sunnucks P, Ward SA. 2003. Explaining the coexistence of asexuals with their sexual progenitors: no evidence for general-purpose genotypes in obligate parthenogens of the peach-potato aphid, *Myzus persicae*. Ecol Lett. 6:1091–1098.

[msac121-B130] Wallig M, Analytics R, Weston S. 2020. doMC: Foreach Parallel Adaptor for “parallel.” Available from: https://CRAN.R-project.org/package=doMC.

[msac121-B131] Wallig M, Microsoft Weston S. 2020. foreach: Provides Foreach Looping Construct. Available from: https://CRAN.R-project.org/package=foreach.

[msac121-B132] Walsh MR . 2013. The link between environmental variation and evolutionary shifts in dormancy in zooplankton. Integr Comp Biol. 53:713–722. 23630969 10.1093/icb/ict035

[msac121-B133] Wang H, Song M. 2011. Ckmeans.1d.dp: optimal *k*-means clustering in one dimension by dynamic programming. R J 3:29–33. 27942416 PMC5148156

[msac121-B134] Weisenfeld NI, Kumar V, Shah P, Church DM, Jaffe DB. 2017. Direct determination of diploid genome sequences. Genome Res. 27:757–767. 28381613 10.1101/gr.214874.116 PMC5411770

[msac121-B135] Wickham H . 2016. ggplot2: elegant graphics for data analysis. 2nd ed. Cham: Springer International Publishing. Imprint: Springer.

[msac121-B136] Wilke C . 2019. Fundamentals of data visualization: a primer on making informative and compelling figures. 1st ed. Sebastopol. CA: O’Reilly Media.

[msac121-B137] Wolinska J, Bittner K, Ebert D, Spaak P. 2006. The coexistence of hybrid and parental *Daphnia*: the role of parasites. Proc R Soc B Biol Sci. 273:1977–1983. 10.1098/rspb.2006.3523 PMC1634769 16822760

[msac121-B138] Wolinska J, Lively CM. 2008. The cost of males in *Daphnia pulex*. Oikos 117:1637–1646.

[msac121-B139] Yang J, Zaitlen NA, Goddard ME, Visscher PM, Price AL. 2014. Advantages and pitfalls in the application of mixed-model association methods. Nat Genet. 46:100–106. 24473328 10.1038/ng.2876 PMC3989144

[msac121-B140] Ye Z, Molinier C, Zhao C, Haag CR, Lynch M. 2019. Genetic control of male production in *Daphnia pulex*. Proc Natl Acad Sci U S A. 116:15602–15609. 31320584 10.1073/pnas.1903553116 PMC6681738

[msac121-B141] Ye Z, Xu S, Spitze K, Asselman J, Jiang X, Ackerman MS, Lopez J, Harker B, Raborn RT, Thomas WK, et al 2017. A new reference genome assembly for the microcrustacean *Daphnia pulex*. Genes Genomes Genet. 7:1405–1416. 10.1534/g3.116.038638 PMC5427498 28235826

[msac121-B142] Yin M, Petrusek A, Seda J, Wolinska J. 2012. Fine-scale temporal and spatial variation of taxon and clonal structure in the *Daphnia longispina* hybrid complex in heterogeneous environments. BMC Evol Biol. 12:12. 22280487 10.1186/1471-2148-12-12 PMC3305588

[msac121-B143] Yin M, Wolinska J, Gießler S. 2010. Clonal diversity, clonal persistence and rapid taxon replacement in natural populations of species and hybrids of the *Daphnia longispina* complex. Mol Ecol. 19:4168–4178. 20819161 10.1111/j.1365-294X.2010.04807.x

[msac121-B144] Zhang D, de Souza RF, Anantharaman V, Iyer LM, Aravind L. 2012. Polymorphic toxin systems: comprehensive characterization of trafficking modes, processing, mechanisms of action, immunity and ecology using comparative genomics. Biol Direct. 7:18. 22731697 10.1186/1745-6150-7-18 PMC3482391

[msac121-B145] Zhang J, Kobert K, Flouri T, Stamatakis A. 2014. PEAR: a fast and accurate Illumina Paired-End reAd mergeR. Bioinformatics 30:614–620. 24142950 10.1093/bioinformatics/btt593 PMC3933873

[msac121-B146] Zheng C, Boer MP, van Eeuwijk FA. 2018. Accurate genotype imputation in multiparental populations from low-coverage sequence. Genetics 210:71–82. 30045858 10.1534/genetics.118.300885 PMC6116951

[msac121-B147] Zheng X, Gogarten SM, Lawrence M, Stilp A, Conomos MP, Weir BS, Laurie C, Levine D. 2017. SeqArray-a storage-efficient high-performance data format for WGS variant calls. Bioinformatics 33:2251–2257. 28334390 10.1093/bioinformatics/btx145 PMC5860110

[msac121-B148] Zheng X, Levine D, Shen J, Gogarten SM, Laurie C, Weir BS. 2012. A high-performance computing toolset for relatedness and principal component analysis of SNP data. Bioinformatics 28:3326–3328. 23060615 10.1093/bioinformatics/bts606 PMC3519454

[msac121-B149] Zimin AV, Marçais G, Puiu D, Roberts M, Salzberg SL, Yorke JA. 2013. The MaSuRCA genome assembler. Bioinformatics 29:2669–2677. 23990416 10.1093/bioinformatics/btt476 PMC3799473

[msac121-B150] Ziyatdinov A, Vázquez-Santiago M, Brunel H, Martinez-Perez A, Aschard H, Soria JM. 2018. lme4qtl: linear mixed models with flexible covariance structure for genetic studies of related individuals. BMC Bioinform. 19:68. 10.1186/s12859-018-2057-x PMC5830078 29486711

