# Supplementary material for: Genetic Variation in Reproductive Investment Across an Ephemerality Gradient in Daphnia pulex
Source: Mol Biol Evol. 2022 Jun 1;39(6):msac121. doi: 10.1093/molbev/msac121 (PMC9198359; doi:10.1093/molbev/msac121)
Supplement: msac121_Supplementary_Data [file msac121_supplementary_data.zip › SupplementalFigures.docx]

**Supplemental Figures**

**Supplemental Figure 1:** Changes in relative water level in D8 (blue) and DBunk (orange) from March of 2019 through January of 2020 along with rainfall events during the same time period. Ponds are observed to slowly dry during spring and early summer due to decreased rainfall, and then rapidly refill in the fall due to heavy rains. Relative changes in water level were measured from daily photographs taken by time lapse cameras posted at D8 and DBunk. Lowest relative water level measurements do not necessarily correspond to completely dry, but to lowest water level observed.


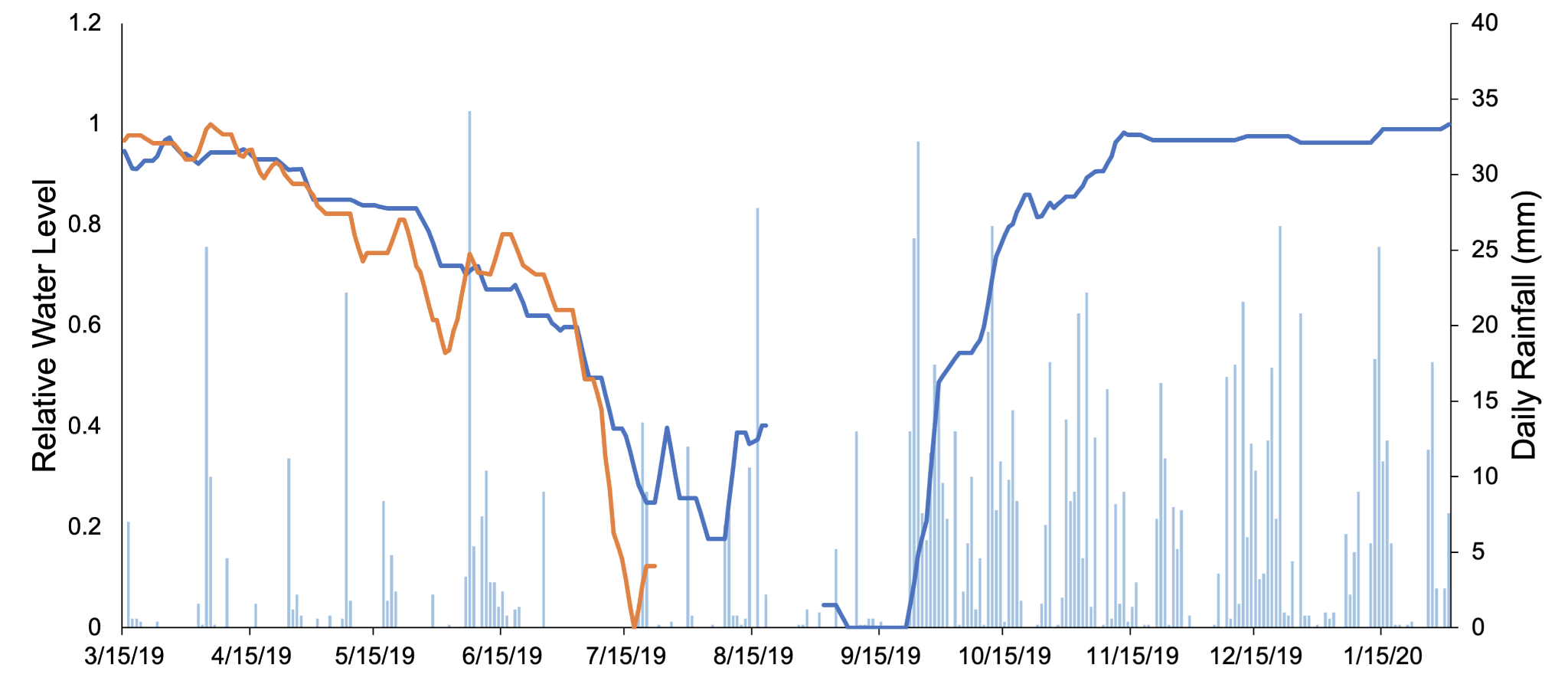


**Supplemental Figure 2: Reference genome link density histogram illustrating 12 scaffolds that correspond to the 12 Daphnia chromosomes.** Mapping positions of pairs of proximity-ligated reads are plotted along the x and y axis, with the number of read pairs within each bin indicated by color. Vertical and horizontal white bars have been added to show the borders between scaffolds. The large scaffolds that show no linkage with the remaining Daphnia scaffolds are microbial genomes present due to a lack of antibiotics being used for the initial 10X Chromium sequencing and assembly.

**
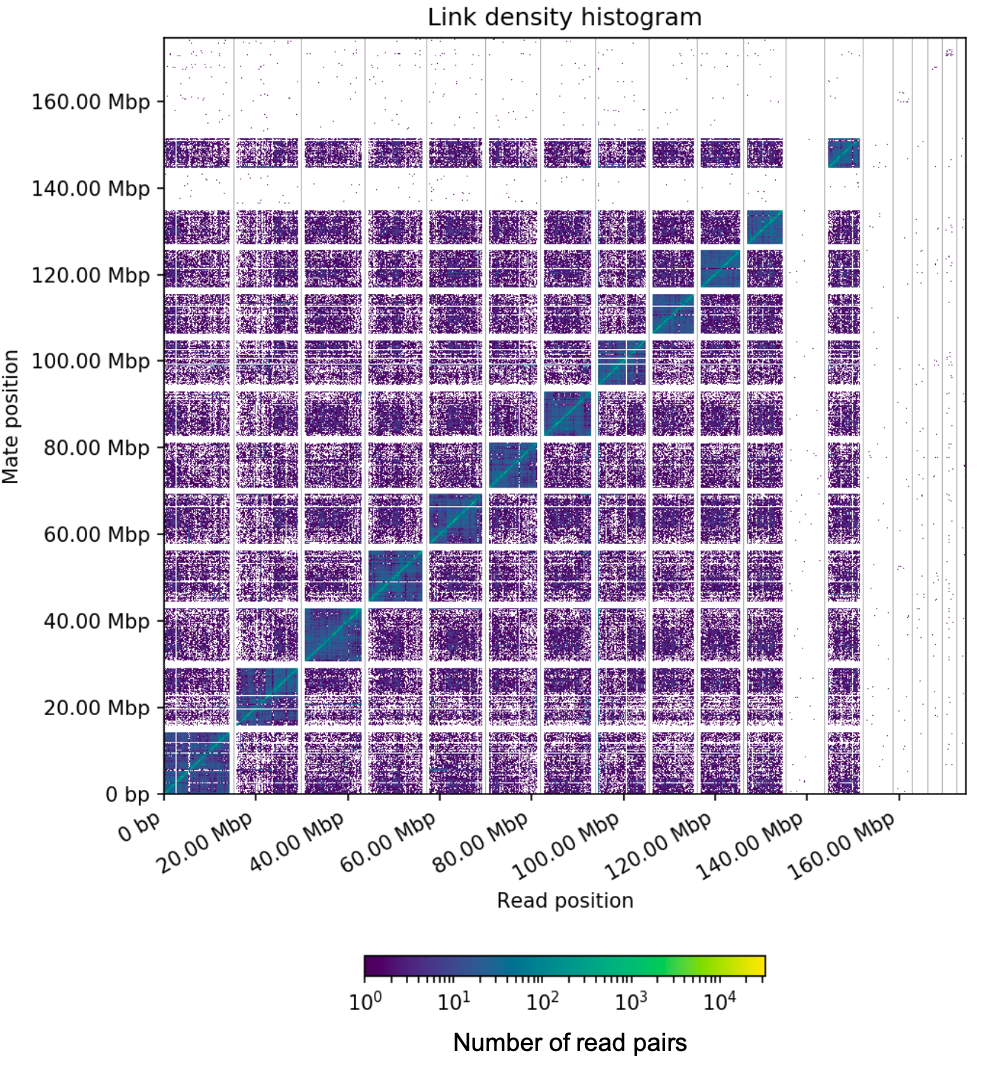
**

**Supplemental Figure 3:** A) Distribution of pairwise IBS values between all sequenced clones with horizontal red line marking the IBS cutoff above which clones were considered to belong to the same clonal lineage. B) Relationship between IBS0 and Kinship as calculated in the program King for pairwise combinations of individuals genotyped from DCat, D8, and DBunk. Only individuals with a median read depth of greater than 14 are included in this graph. Colored points represent prior determined relationships that were used to demarcate clustering and identify additional parent-offspring relationships that were used for constructing the pedigree in Figure 2. C) and D) Examples of outlier individuals within clonal lineages identified by IBS that were subsequently removed from the clonal lineages. E) Zoomed out version of panel B, with A superclone vs C superclone points (in pink) added to illustrate their relationship.

**
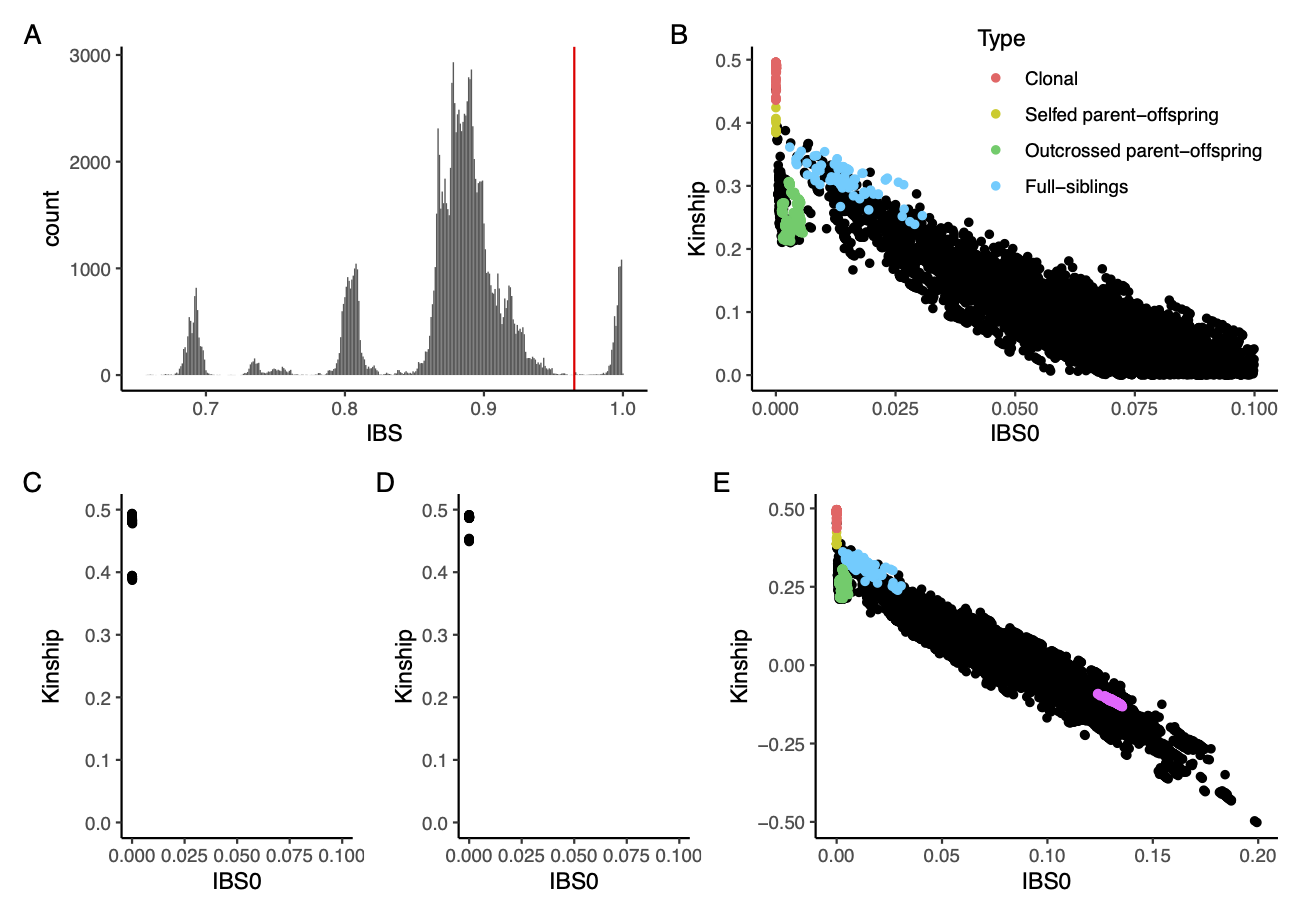
**

**Supplemental Figure 4:** Clonal diversity for each pond by year. Diversity was calculated using Shannon’s diversity index in the R package *vegan* (Oksanen et al. 2020).

**
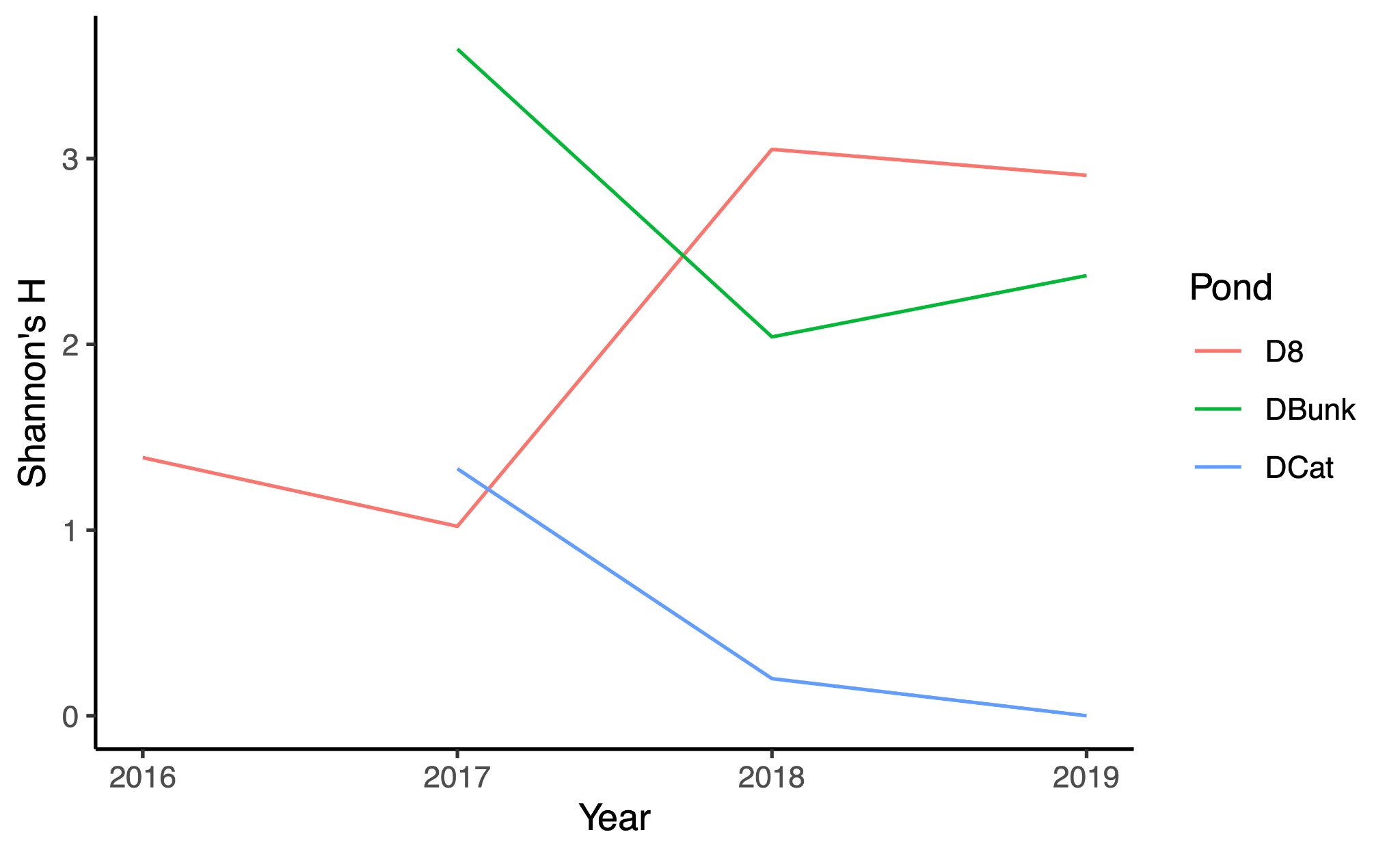
**

**Supplemental Figure 5. Mutation accumulation**. We calculated the ratio of synonymous to non-synonymous mutations at different frequency classes within the larger superclone assemblages (A-L), or among singleton isofemale lines (2016/17) or wild caught individuals (2018/2019). For each group, we randomly selected eight genomes and generated confidence intervals (95%) via bootstrap resampling. See Materials and Methods for details. We identified mutations that were segregating among the sample of genomes, e.g. polymorphism among different field isolates of superclone A. We partitioned those polymorphisms into two classes: New mutations that are segregating at “1/2N” frequency; or, mutations that were shared heterozygotes (“~0.5”). We classified shared heterozygous sites as those where at least 7 (out of 8) individuals were heterozygous. New alleles that arise in asexual lineages are expected to have a higher proportion of non-synonymous sites than older, shared polymorphism. Additionally, the sequencing libraries generated from the 2016/2017 collections were made from isofemale lines that had been propagated in the lab for 4-12 generations, and each library contained ~8-12 individuals. The 2018/2019 libraries were made from single field preserved individuals. The higher pn/ps of the 2016/2017 collections compared to the field caught individuals from 2018/2019 is explainable by these sample features.


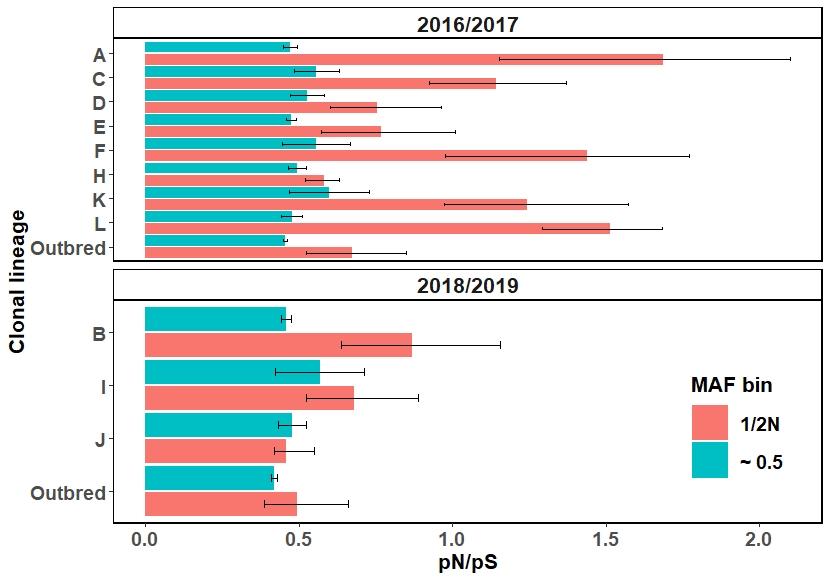


**Supplemental Figure 6:** Estimates of within-individual genome-wide average heterozygosity across the three focal ponds. Each point represents the estimated heterozygosity for an individual clone. Diversity is stable through time in DCat and DBunk, but fluctuates in D8 due to cycles of inbreeding and outbreeding. Panels are ordered by increasing ephemerality.

**
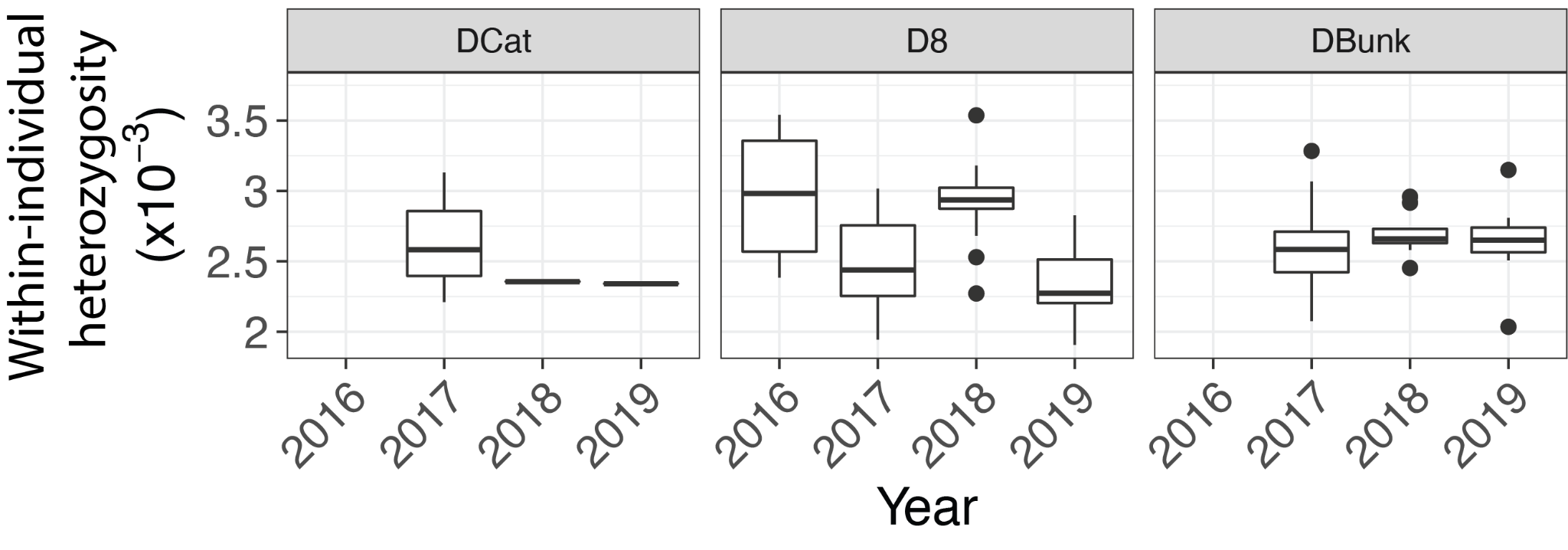
**

**Supplemental Figure 7**: Evidence based on genome-wide patterns of IBS that A and C are more genetically different than most pairwise comparisons of clonal lineages from the focal ponds, but are not as different as focal pond clonal lineages compared to more distant *D. pulex* populations. IBS was calculated between all pairs of clonal lineages in sliding windows (window size=250,000, step size = 10,000). Then, for each window, IBS for A versus C was divided by the calculated IBS for each pair of comparison clonal lineages to obtain an IBS ratio. Finally, a mean, genome-wide similarity ratio was calculated by averaging across windows for each clonal lineage comparison. Each point in the graph below represents a mean, genome-wide IBS ratio for a pair of clonal lineages, with values less than one indicating that the comparison pair of clones is on average more closely related than A and C, whereas values greater than one indicate the pair of clones is more distantly related. Within D8 are comparisons between clonal lineages found within D8. Within DCat/DBunk are comparisons between clonal lineages within DCat and within DBunk, comparing clonal lineages found within the same pond. Between DCat/D8/DBunk are comparisons of clonal lineages found in different focal ponds. Kilwood vs D10, Kilwood vs W1, and Kilwood vs W6 are comparisons of clonal lineages found in the focal ponds versus clonal lineages found in D10, W1, and W6 respectively.


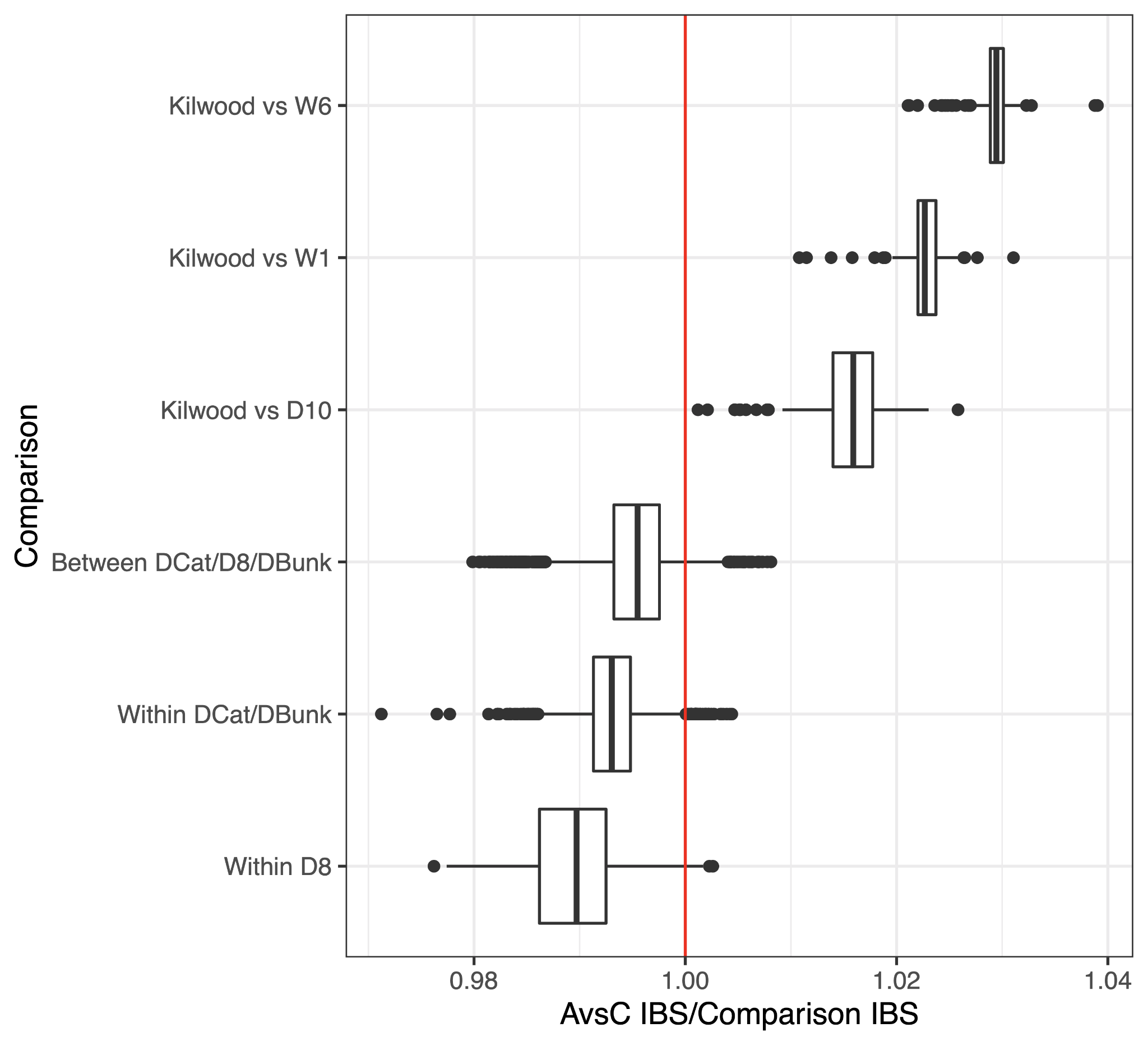


**Supplemental Figure 8. Introgression & hybridization analysis.** (A) Structure-type analysis performed using *snmf* function in LEA (see Material and Methods). Each color represents the inferred ancestry coefficient. (B) Cross-entropy analysis suggests that eight clusters is the optimal number of clusters for ancestry analysis. The two out-group species, *D. obtusa* and *D. pulicaria* were assigned to unique clusters; unique clusters were also identified for the Welsh individuals (W) and for individuals sampled from D10. The remaining four clusters were assigned to the Kilwood ponds. (C) and (D) represent the inferred proportion of *D. pulicaria* and *D. obtusa* admixture for the *D. pulex* individuals. Overall, there was a trivial amount of admixture. (E) Schematic of the tree configuration for the *f4*-ratio and D test; P1 and P2 are the population labels for *D. pulex* samples as noted in (A). (F) Several samples show values of D that are statistically different from zero (x-axis), but nonetheless show trivial amounts of ancestry (y-axis).

**
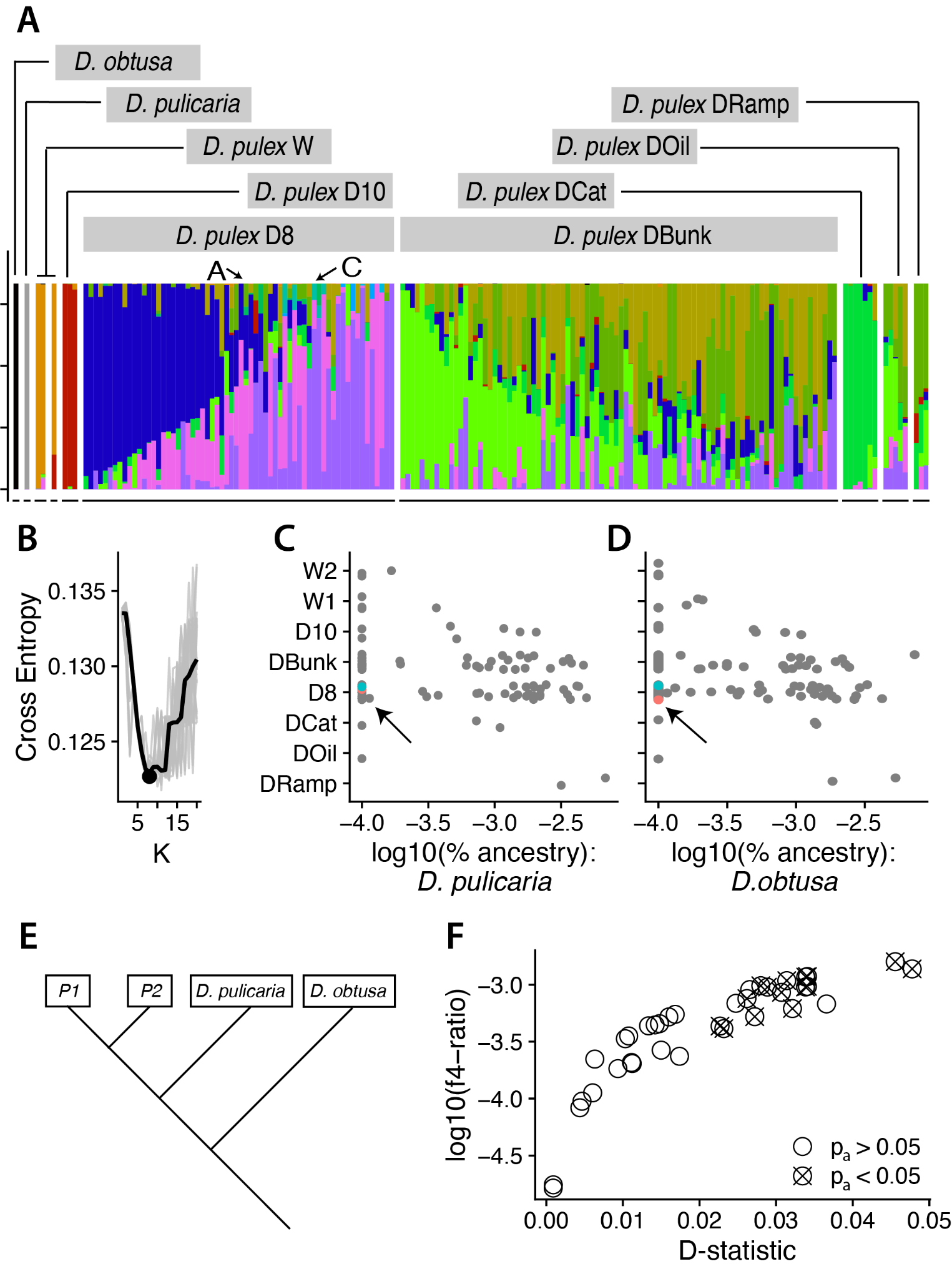
**

**Supplemental Figure 9**: Male production of A and C isofemale lines in 250 ml jars. Jars were started with individual neonates, allowed to expand, and then followed over 12 weeks. Media was changed at 5 and 8 weeks. Data shown is the estimated proportion of males in each jar at 12 weeks. (N=26, 25, 30, and 25 for D8-179, D8-349, D8-222, and D8-515 respectively). Data are plotted by isofemale line, with two isofemales lines per superclone A and C. There was a significant effect of superclone on male production (likelihood ratio test, 𝜒^2^=7.5, df=1, *p*=0.0063).

**
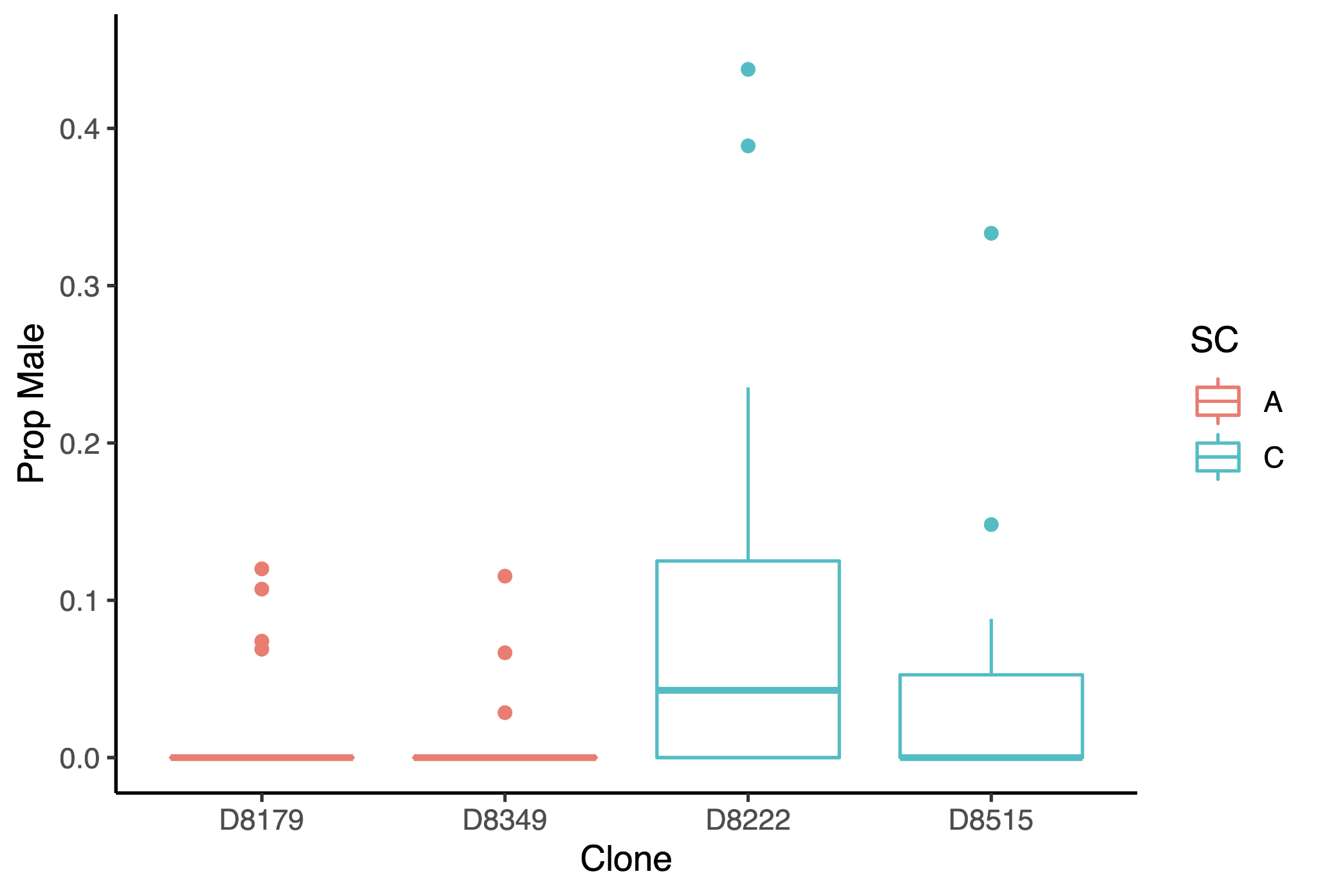
**

**Supplemental Figure 10.** Linkage disequilibrium estimated between tag-SNPs (see Materials and Methods section “QTL mapping using F1s”) associated with QTL (blue) or between the remaining SNPs (red) in the genome. Panel A shows the results for ephippial fill rate. Panel B shows the results for male production rate. As expected, linkage is higher between markers on the same chromosome than between chromosomes (*t = 557.02, df = 1451174, p-value < 2.2e-16)*. Linkage estimates between QTL tag-SNPs on the same chromosome are significantly higher than non-tag-SNPs for ephippial fille rate (t = 14.159, df = 704.54, p-value < 2.2e-16*)* and for male production rate (t = 19.243, df = 537.32, p-value < 2.2e-16). Linkage estimates between QTL tag-SNPs on different chromosome are significantly higher than non-tag-SNPs for ephippial fille rate (t = 9.0548, df = 2670, p-value < 2.2e-16*)* and for male production rate (t = 13.969, df = 1711.3, p-value < 2.2e-16). Although there is higher linkage between QTL tag-SNPs on separate chromosomes, the effects are relatively small compared to the within chromosome elevation, suggesting that at least some of these QTL are acting independently. Panel C and D show the pairwise LD between QTL tag-SNPs for [C] ephippial file rate and (D) male production rate.


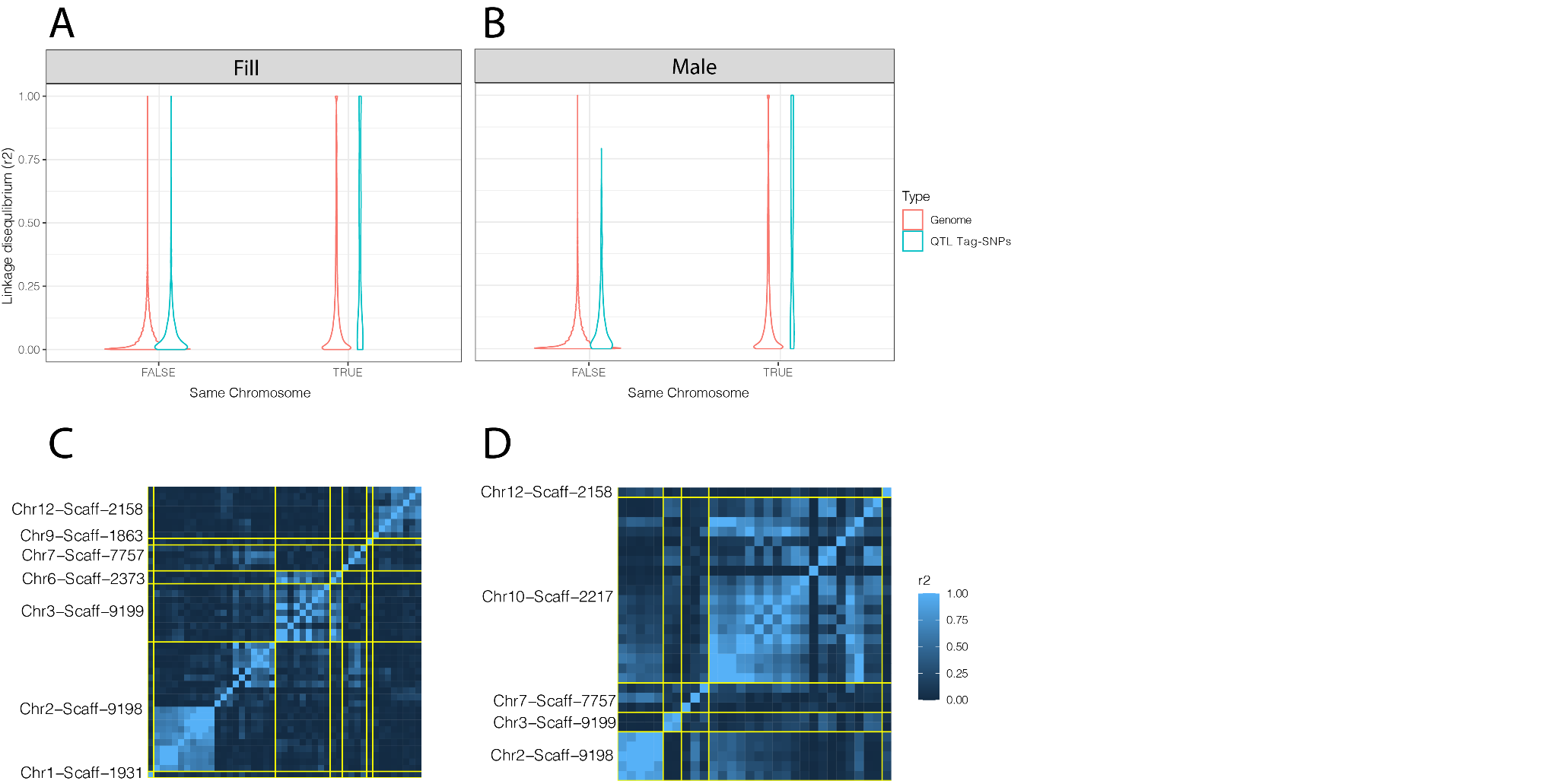


**Supplemental Figure 11**. QTL mapping results from CxC F1 cross. The top row reports the QTL mapping of ephippial fill rate and the bottom row reports the QTL mapping results of male production rate. The vertical lines are the 14 QTL identified via Pool-Seq. The horizontal lines represent the chromosome specific permutation thresholds. The black dots represent markers associated with either phenotype above the permutation threshold.

**
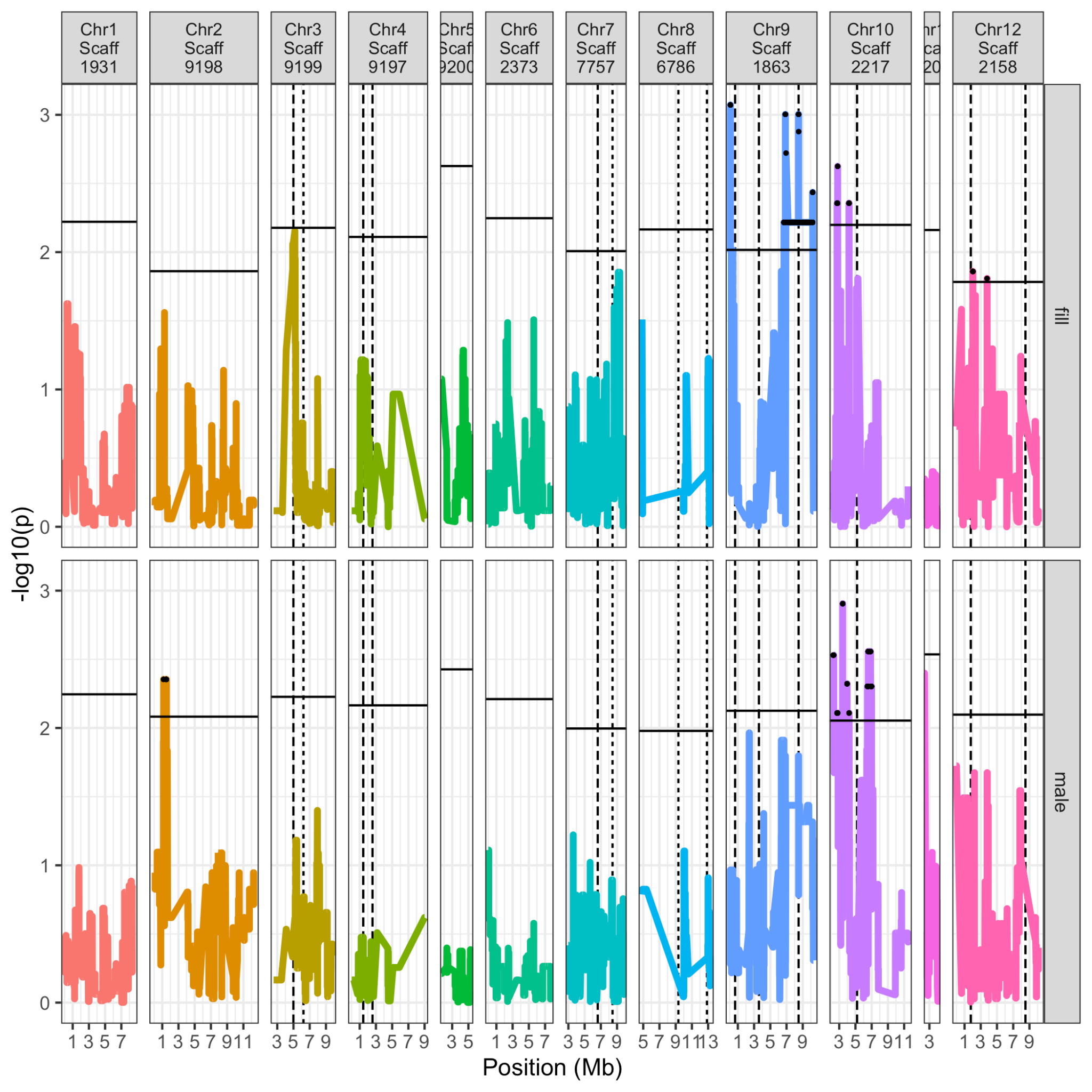
**

**Supplemental Figure 12:** Pooled sequencing individuals are predominantly AxC F1 hybrids. Each facet represents one of the nine possible genotypic states in the two parental clones. The number represents the dosage of reference alleles. The red horizontal lines are the expected frequencies of the reference alleles in F1 offspring for each combination of genotypic states. Points represent the observed average frequencies in the field collected pools. Error bars represent one standard deviation.


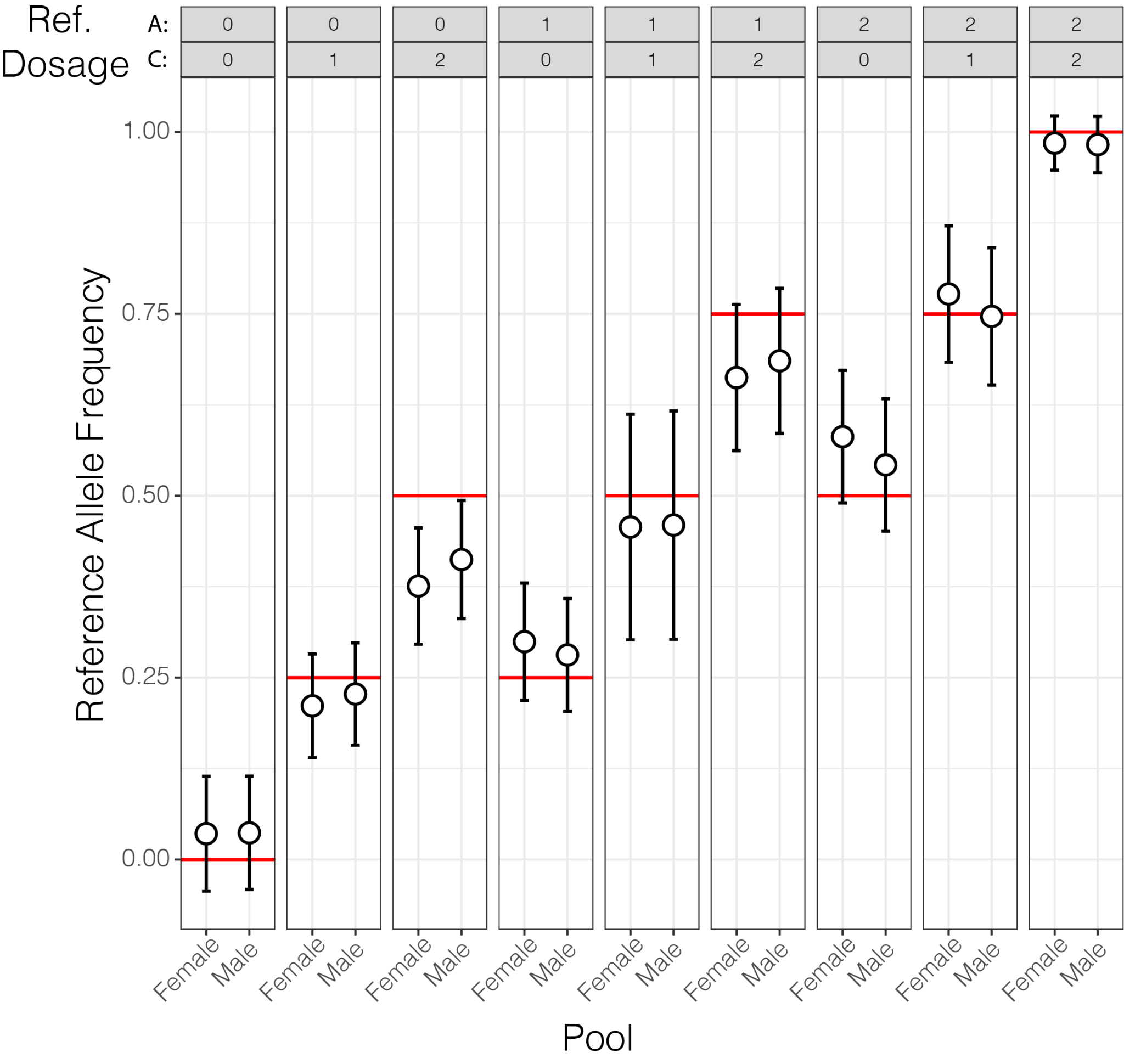


**Supplemental Figure 13. Overlap analysis between the QTL identified in F1 mapping and PoolSeq.** We generated the null distribution of overlap test statistics, defined by the regioneR package (Gel et al. 2016), using permutation (n=100). The permutations were performed at the QTL mapping stage (see Materials and Methods). The red line indicates the observed overlap statistic for the AxC cross for that particular phenotype. The observed overlap statistic for ephippial fill rate is greater than expected by chance (*p*=0.01).


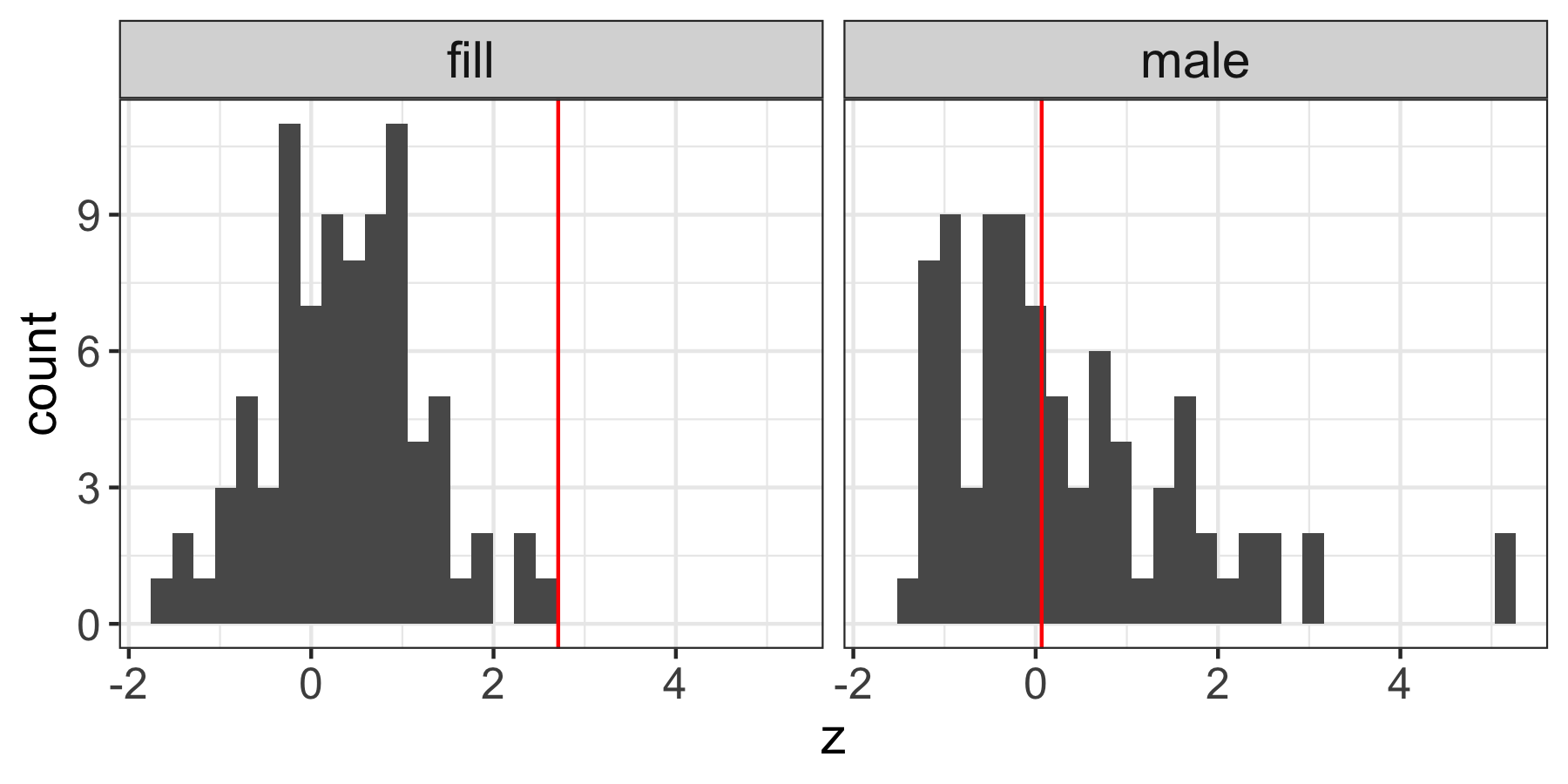


**Supplemental Figure 14:** Relationship between genotype and male production for AxC F1 and CxC clones at the 4 pool-seq QTL where genotype had a significant effect on male production (p<0.05). QTL 9 and 12 remained significant after Bonferroni correction. Blue lines represent selfed C clonal lineages, while red lines represent AxC F1 hybrid clonal lineages. Error bars are 95% confidence intervals.


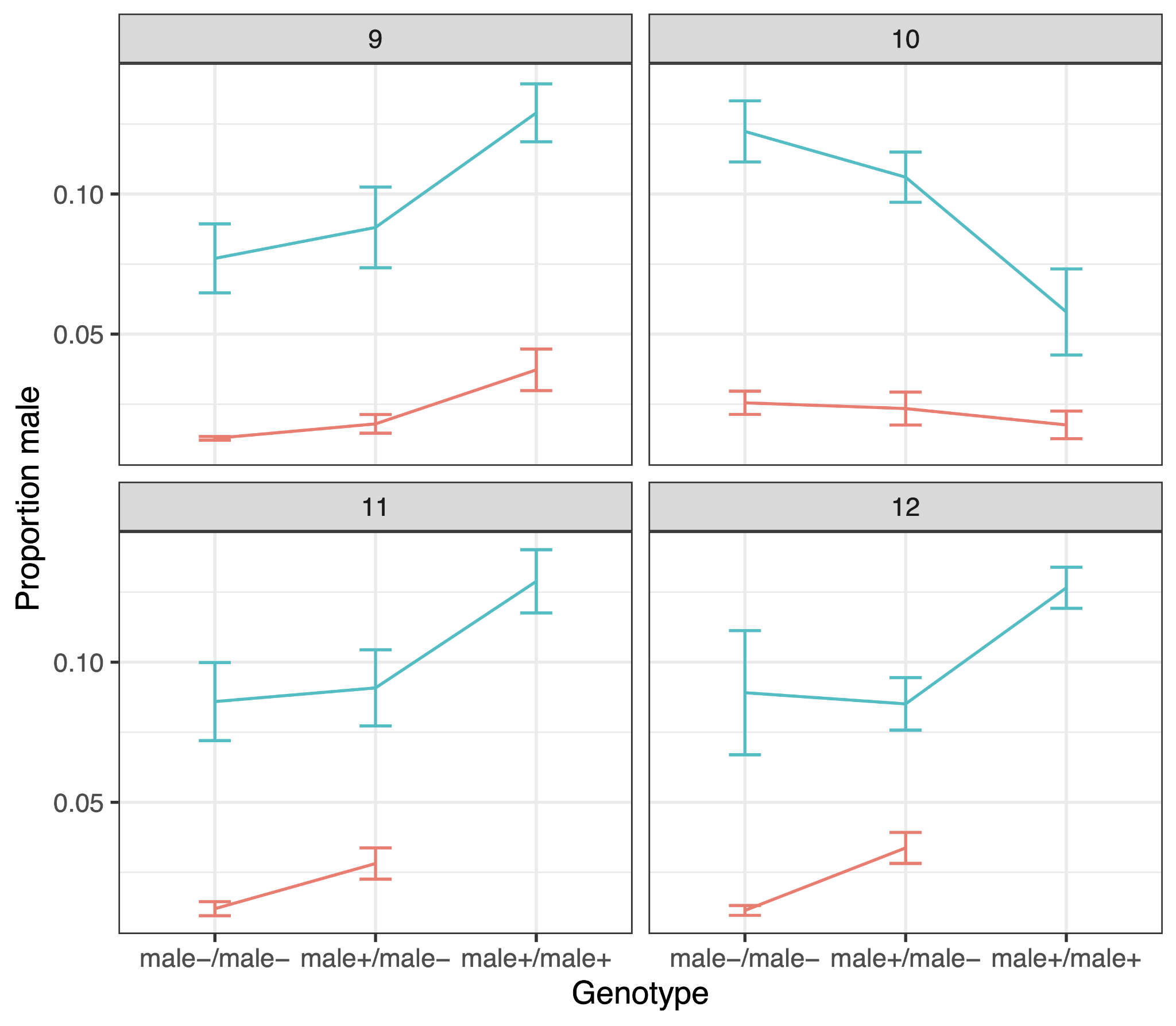


**Supplemental Figure 15:** Frequency of *male^+^* allele at each QTL in the three focal ponds across three sample years. Frequency was calculated across all individuals sampled (no subsetting of superclones). Error bars represent 95% confidence intervals.


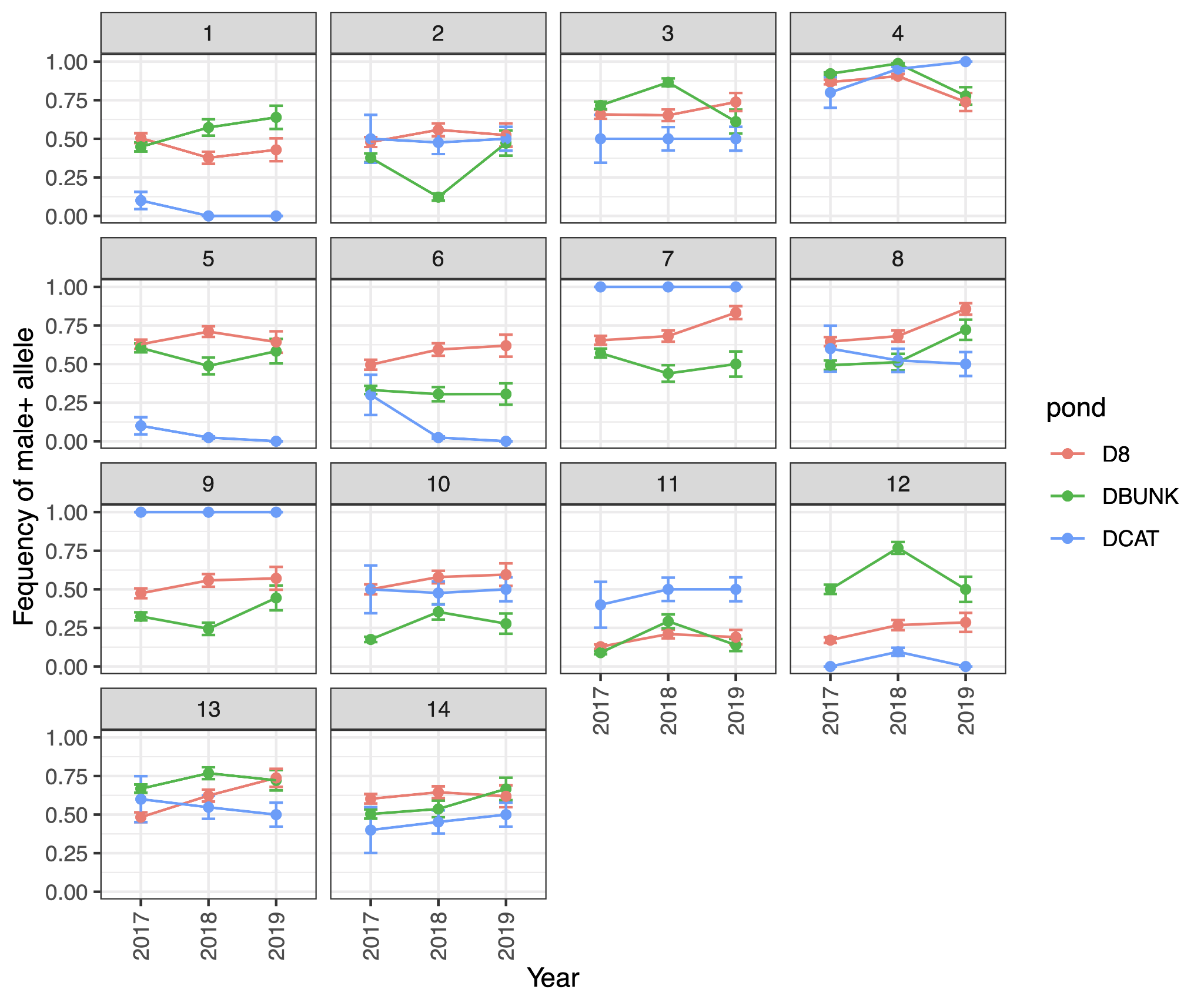


**Supplemental Figure 16**. Differential expression between superclones A and C. (A) Principal component analysis shows that PC1 clearly separates superclone A and C, and explains 60% of the variance in gene expression. Clones within a superclone also clustered together. (B) The distribution of p-values contrasting differential expression between superclones. There are many differentially expressed genes. [C] shows the gene expression differences between superclones A and C for the 4 adjacent genes which are strongly differentially expressed. (D) IGView screenshot of the four-gene region from stranded RNA-seq libraries used for gene-model prediction. This screen-shot demonstrates that *Daphnia00787* is intronless, although there are spliced antisense transcripts possibly associated with *Daphnia00788* or *Daphnia00789*.


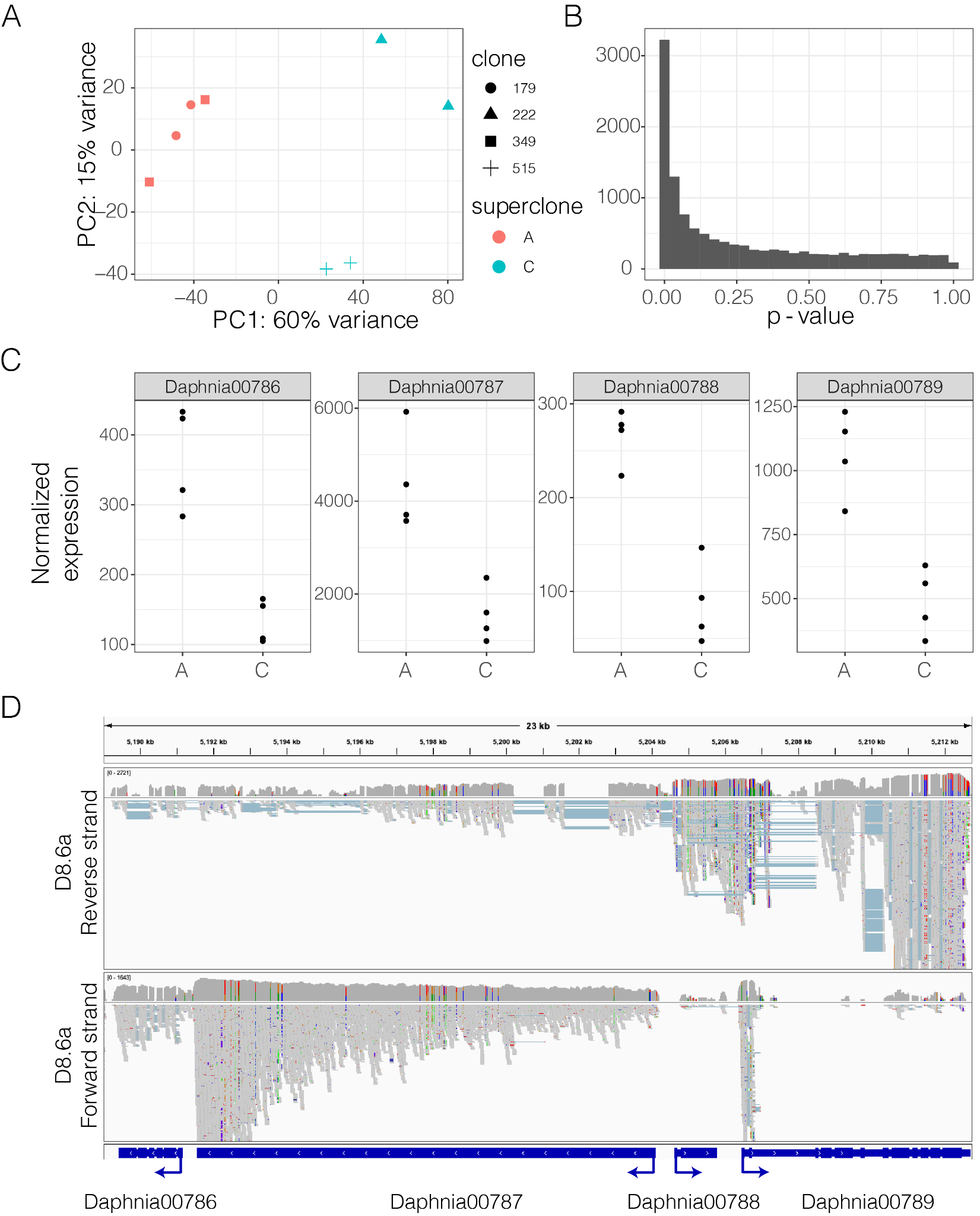


**Supplemental Figure 17:** Comparison of male production for superclone B, the dominant superclone in the permanent DCat pond, relative to male production for other genotypes (data from Figure 4). Superclone B exhibits low levels of male production, similar to superclone A. Male production was measured in one liter jars consistently maintained at high densities (see Methods: Generation and phenotyping of F1 AxC and CxC offspring). Points represent isofemale lines and vertical lines represent 95% confidence intervals.

**
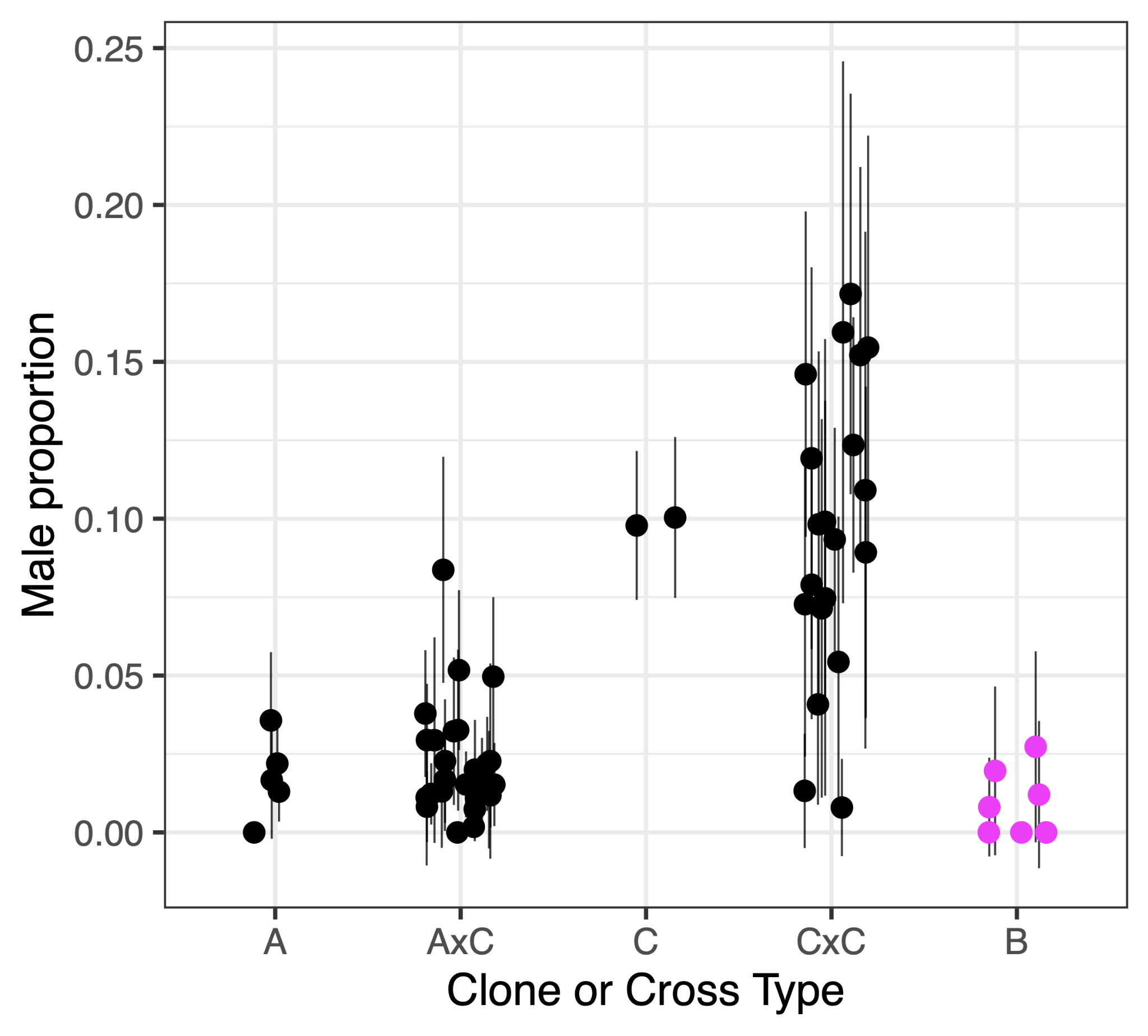
**
