# Supplementary material for: Genetic Variation in Reproductive Investment Across an Ephemerality Gradient in Daphnia pulex
Source: Mol Biol Evol. 2022 Jun 1;39(6):msac121. doi: 10.1093/molbev/msac121 (PMC9198359; doi:10.1093/molbev/msac121)
Supplement: msac121_Supplementary_Data [file msac121_supplementary_data.zip › SuppTable1.docx]

**Table S1:** Relative water level of the focal ponds across sample time points.

| Date | D10 | Dcat | D8 | DBunk |
| --- | --- | --- | --- | --- |
| 4/11/17 | high | high | high | mid |
| 3/20/18 | high | high | high | high |
| 4/5/18 | high | high | high | high |
| 6/1/18 | high | high | high | puddle |
| 6/13/18 | high | high | mid | dry |
| 6/28/18 | high | high | puddle | dry |
| 7/12/18 | high | high | puddle | dry |
| 7/25/18 | high | high | puddle | dry |
| 10/30/18 | mid | mid | puddle | dry |
| 12/20/18 | not visited | high | high | high |
| 3/15/19 | high | high | high | high |
| 3/30/19 | high | high | high | high |
| 4/29/19 | high | high | high | mid |
| 5/11/19 | high | high | high | mid |
| 6/5/19 | high | high | mid | puddle |
| 7/4/19 | high | high | mid | puddle |
| 7/17/19 | high | mid | puddle | dry |
| 2/2/20 | high | high | high | high |
