# Supplementary material for: Genetic Variation in Reproductive Investment Across an Ephemerality Gradient in Daphnia pulex
Source: Mol Biol Evol. 2022 Jun 1;39(6):msac121. doi: 10.1093/molbev/msac121 (PMC9198359; doi:10.1093/molbev/msac121)
Supplement: msac121_Supplementary_Data [file msac121_supplementary_data.zip › SuppTable2.docx]

**Table S2:** Summary information for experiments

| Experiment | Sample Size | | Replication | Figures | Tables | Data Files | Accession Numbers? |
| --- | --- | --- | --- | --- | --- | --- | --- |
| Reference genome | NA | | NA | Figure S2 | Table S3 | 10 Chromium Reads  Minion Reads  Genome Sequence  GFF file  Annotation file  BED file of repeat masker excluded regions  BED file of further excluded regions | SRR14333786  SRR14567272  JAHCQT000000000  <https://doi.org/10.5061/dryad.rr4xgxd87/Daphnia.aed.0.6.gff>  [https://doi.org/10.5061/dryad.rr4xgxd87](https://doi.org/10.5061/dryad.rr4xgxd87/Daphnia.aed.0.6.gff)/Daphnia_annotation_PANTHER.xls  https://doi.org/10.5061/dryad.rr4xgxd87/RMoutHiCGMgoodscaff.bed  <https://doi.org/10.5061/dryad.rr4xgxd87/NsandDepthandChrEnd.sorted.500merged.bed> |
| Individual sequencing of individuals from the field | D10 - 2016: 30  DCat - 2017: 5  DCat - 2018: 21  DCat - 2019: 20  D8 - 2016: 21  D8 - 2017: 117  D8 - 2018: 69  D8 - 2019: 21  DBunk - 2017: 135  DBunk - 2018: 41  DBunk - 2019: 18  W1 - 2016: 1  W6 - 2016: 2  *D. obtusa* - 15  *D. pulicaria* - 5 | | NA | Figure 2  Figure 3  Figure S3  Figure S4  Figure S5  Figure S6  Figure S7  Figure S8 | Table S1 | *D. pulex* Illumina reads  *D. obtusa* Illumina reads  *D. pulicaria* Illumina reads  VCF file of SNPs  SeqArray GDS file of SNPs  SNPRelate GDS file of SNPs  Table of "total filtered SNP set"  Table of "variable *pulex* SNP set"  Table of "LD pruned, variable pulex SNP set" | SAMN19021176-SAMN19021270  SAMN19067418-SAMN19067740  SAMN19088632-SAMN19088720  SAMN19102355-SAMN19102360  SAMN18938157-SAMN18938171  SAMN18940763-SAMN18940767  https://doi.org/10.5061/dryad.rr4xgxd87/MapJune2020_ann.vcf.gz  https://doi.org/10.5061/dryad.rr4xgxd87/MapJune2020_ann.seq.gds  https://doi.org/10.5061/dryad.rr4xgxd87/MapJune2020_ann.gds  https://doi.org/10.5061/dryad.rr4xgxd87/dpfiltsnpsdt.csv  https://doi.org/10.5061/dryad.rr4xgxd87/snpsvarpulexpresentinhalf_table_20200623  <https://doi.org/10.5061/dryad.rr4xgxd87/finalsetsnpset01pulex_table_20200623> |
| Phenotypic differentiation between A and C | 4 isofemale lines (2 A + 2 C) | | 2 per isofemale line | Figure 4 |  | Phenotypic dataset | [https://doi.org/10.5061/dryad.rr4xgxd87/Mesocosm2019Data.csv](https://doi.org/10.5061/dryad.rr4xgxd87) |
| Male production in response to methyl farnesoate | 4 isofemale lines (2 A + 2 C) | | 5-11 per isofemale line | Figure 4 |  | Methyl farnesoate data | [https://doi.org/10.5061/dryad.rr4xgxd87/FinalNeoNotAdj.csv](https://doi.org/10.5061/dryad.rr4xgxd87) |
| Male production in 250 ml jars - A and C | 4 isofemale lines (2 A + 2 C) | | 25-30 per isofemale line | Figure S9 |  | Phenotypic dataset | [https://doi.org/10.5061/dryad.rr4xgxd87/250mlMales12Weeks.csv](https://doi.org/10.5061/dryad.rr4xgxd87) |
| Sequencing and phenotyping of F1 AxC and CxC offspring for QTL mapping | CxC: 24 isofemale lines  AxC: 26 isofemale lines  A and C: 2 isofemale lines each  B (DCat): 7 isofemale lines | | CxC: 1-3 per isofemale line  AxC: 1-3 per isofemale line  A and C: 5 per isofemale line  B: 1 per isofemale line | Figure 5  Figure 6  Figure S10  Figure S11  Figure S13  Figure S17 |  | Illumina Reads  One liter ephippia data  One liter male production data | SAMN18927638-SAMN18927680  https://doi.org/10.5061/dryad.rr4xgxd87/OneLitersEphippiaFinal.csv  <https://doi.org/10.5061/dryad.rr4xgxd87/OneLitersMaleCensus.csv> |
| Pool-Seq of males and females with parthenogenic embryos from D8 for QTL mapping | | Males: 35 individuals per pool  Females: 50 individuals per pool | Males: 2 pools of 35  Females: 2 pools of 50 | Figure 5  Figure S12  Figure S13 | Table S4 | Illumina reads | SRR14559758-SRR14559761 |
| RNAseq of A and C | | 4 isofemale lines (2 A + 2 C) | 2 per isofemale line | Figure 5  Figure 6  Figure S16 |  | Illumina reads | SRR14572418-SRR14572425 |
