# Supplementary material for: Genetic Variation in Reproductive Investment Across an Ephemerality Gradient in Daphnia pulex
Source: Mol Biol Evol. 2022 Jun 1;39(6):msac121. doi: 10.1093/molbev/msac121 (PMC9198359; doi:10.1093/molbev/msac121)
Supplement: msac121_Supplementary_Data [file msac121_supplementary_data.zip › SuppTable3Legend.docx]

**Table S3:** List of all genomes sequenced with the following information: NCBI accession number; superclone assignment (OO indicates clonal lineages only sampled one time); sample population; sample year; sample month; median read depth; whether or not the individual was fixed in ethanol in the field (1) or established in the lab prior to sequencing (0, WildSequenced); sex; species; is (1) or is not (0) an AxC F1 hybrid; whether or not the clone was lab generated (the result of crossing in the lab); whether or not the clone was phenotyped in the one liter experiment; and whether or not the clone has low read depth and was excluded from most analyses.
