# Supplementary material for: Genetic Variation in Reproductive Investment Across an Ephemerality Gradient in Daphnia pulex
Source: Mol Biol Evol. 2022 Jun 1;39(6):msac121. doi: 10.1093/molbev/msac121 (PMC9198359; doi:10.1093/molbev/msac121)
Supplement: msac121_Supplementary_Data [file msac121_supplementary_data.zip › SuppTable4Legend.docx]

**Table S4:** Functional gene annotation information for the reference genome.
