# Supplementary material for: Genetic Variation in Reproductive Investment Across an Ephemerality Gradient in Daphnia pulex
Source: Mol Biol Evol. 2022 Jun 1;39(6):msac121. doi: 10.1093/molbev/msac121 (PMC9198359; doi:10.1093/molbev/msac121)
Supplement: msac121_Supplementary_Data [file msac121_supplementary_data.zip › SuppTable5.docx]

**Table S5:** List of the 12 D84.A chromosomes, their length, and which North American Daphnia pulex chromosomes they correspond to (PA42, (Ye et al. 2017b)

| Chromosome | Length | PA42_Chromosome |
| --- | --- | --- |
| Scaffold_1931_HRSCAF_2197 | 8597654 | 1 |
| Scaffold_9198_HRSCAF_10754 | 12365322 | 2 |
| Scaffold_9199_HRSCAF_10755 | 11497646 | 3 |
| Scaffold_9197_HRSCAF_10753 | 9146106 | 4 |
| Scaffold_9200_HRSCAF_10757 | 10326160 | 5 |
| Scaffold_2373_HRSCAF_2879 | 7726101 | 6 |
| Scaffold_7757_HRSCAF_8726 | 11802501 | 7 |
| Scaffold_6786_HRSCAF_7541 | 13322843 | 8 |
| Scaffold_1863_HRSCAF_2081 | 10446993 | 9 |
| Scaffold_2217_HRSCAF_2652 | 14299301 | 10 |
| Scaffold_9201_HRSCAF_10758 | 6810281 | 11 |
| Scaffold_2158_HRSCAF_2565 | 10306748 | 12 |
