# Supplementary material for: Genetic Variation in Reproductive Investment Across an Ephemerality Gradient in Daphnia pulex
Source: Mol Biol Evol. 2022 Jun 1;39(6):msac121. doi: 10.1093/molbev/msac121 (PMC9198359; doi:10.1093/molbev/msac121)
Supplement: msac121_Supplementary_Data [file msac121_supplementary_data.zip › SuppTable6.docx]

**Table S6:** Information regarding the male and female Pool-Seq samples and libraries. N= number of individuals used. Median values are listed for coverage and effective coverage. Effective coverage was calculated as (n.reads * n.chr) / (n.reads + n.chr).

| TimePoint | Pond | Name | Sex | N | Coverage | Effective Coverage | Accession Number |
| --- | --- | --- | --- | --- | --- | --- | --- |
| 4/29/18 | D8 | D8PE1 | Females with parthenogenic embryos | 50 | 81 | 45 | [SRR14559761](https://dataview.ncbi.nlm.nih.gov/object/SRR14559761) |
| 4/29/18 | D8 | D8PE2 | Females with parthenogenic embryos | 50 | 324 | 76 | [SRR14559760](https://dataview.ncbi.nlm.nih.gov/object/SRR14559760) |
| 4/29/18 | D8 | D8Male1 | Males | 35 | 350 | 58 | [SRR14559759](https://dataview.ncbi.nlm.nih.gov/object/SRR14559759) |
| 4/29/18 | D8 | D8Male2 | Males | 35 | 205 | 52 | [SRR14559758](https://dataview.ncbi.nlm.nih.gov/object/SRR14559758) |
